# Select earthquake forecasting models demonstrate consistency with prospective decadal observations in California

**DOI:** 10.1038/s41467-026-76243-7

**Published:** 2026-08-06

**Authors:** José A. Bayona, Francesco Serafini, Fábio Silva, Pablo Iturrieta, William H. Savran, Marcus Herrmann, Warner Marzocchi, Philip J. Maechling, Maximilian J. Werner

**Affiliations:** 1 School of Earth Sciences, University of Bristol, Bristol, UK; 2 Statewide California Earthquake Center, Los Angeles, CA USA; 3 Helmholtz Centre for Geosciences (GFZ Postdam), Potsdam, Germany; 4 Nevada Seismological Laboratory, University of Nevada Reno, Reno, NV USA; 5 University of Naples Federico II, Naples, Italy; 6 Present Address: Global Earthquake Model (GEM) Foundation, Pavia, Italy

**Keywords:** Seismology, Natural hazards

## Abstract

Earthquake forecasting systems are now operationalized by agencies in several countries, providing situational awareness to at-risk communities. To ensure that forecasts follow the best science available, the underlying models require rigorous testing against prospective data and benchmarking. Between August 2007 and August 2018, 27 forecasting models, including the types used for operational earthquake forecasting, were prospectively operated as part of a multi-institutional project by the Collaboratory for the Study of Earthquake Predictability, generating 51,625 next-day M_w_ ≥ 3.95 seismicity forecasts for California. Here, we comprehensively evaluate their predictive skills against 597 M_w_ ≥ 3.95 earthquakes using community-vetted statistical methods. We show that approximately 95%, two-thirds, and 95% of the models produced forecasts consistent with the number, locations, and magnitudes of observed earthquakes, respectively. Individually, select versions of well-established, operational-type models demonstrated sustained consistency with prospective observations. We provide detailed recommendations, software tools, and benchmarking data to support the development of operational earthquake forecasting systems worldwide.

## Abstract (web summary)

This paper demonstrates that next-day seismicity forecasts generated by operational-type models are statistically consistent with decadal prospective observations in California, providing compelling support for their use in public operational earthquake forecasting.

## Introduction

Seismologists are often asked “When will the next big one occur?” an answer to which requires providing precise information (and uncertainties) about the timing, location, and magnitude of future earthquakes^1^. Numerous peer-reviewed studies suggest that diagnostic seismic precursor signals could be routinely observable worldwide and that, consequently, large earthquakes might be predictable^2^. However, these studies rely heavily on retrospective observations, meaning their conclusions can only be considered robust enough after independent prospective evaluations^3^. Thus, the lack of successful deterministic predictive models of earthquake occurrence has led seismologists, including members of the Collaboratory for the Study of Earthquake Predictability (CSEP)^4^, to devote scientific efforts to forecasting earthquakes probabilistically^5,6^.

**Fig. 1:**
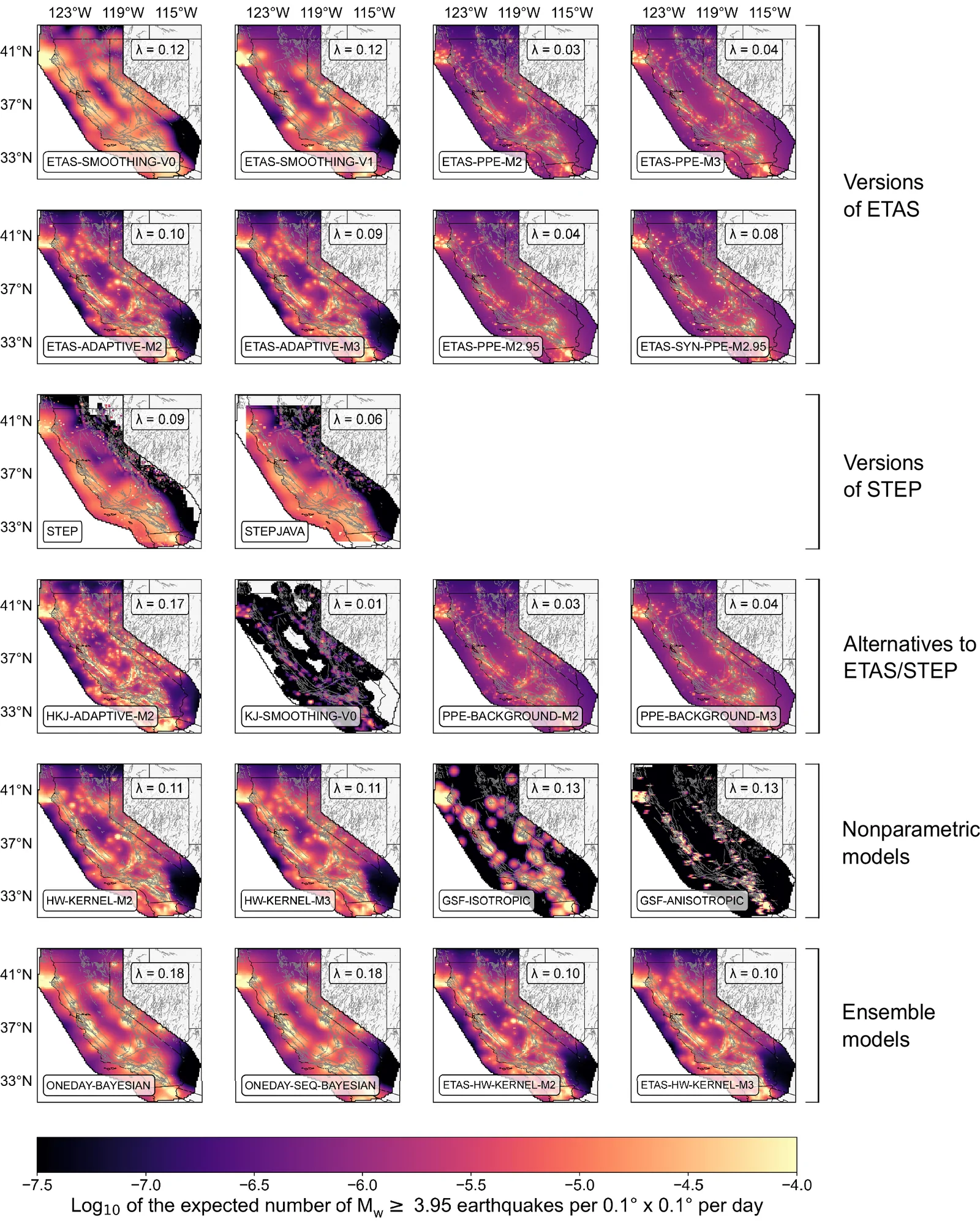
Forecast maps generated by 21 *M*_w_ ≥ 3.95 next-day seismicity models and one time-invariant model (HKJ-ADAPTIVE-M2) for four different seismically calm days in California. Earthquake rates are expressed per 0.1^∘^ × 0.1^∘^ cell per day, with bright colors denoting regions where seismic activity is comparatively high, dark colors indicating comparatively low rates, and white areas denoting regions where the models are not defined. Since the evaluated models were developed and submitted to CSEP at different times^7^, this example shows a STEP forecast issued on January 21, 2013, ONEDAY(-SEQ)-BAYESIAN forecasts issued on August 7, 2016, and ETAS-ADAPTIVE-M2, ETAS-HW-KERNEL-M2, and HW-KERNEL-M3 forecasts created on September 19, 2016. The remaining forecasts were created on August 30, 2018. Annotated *λ* values indicate the number of *M*_w_ ≥ 3.95 earthquakes expected by the models during those specific days. Fault traces (shown in gray) are obtained from the USGS Quaternary Fault and Fold Database (see the “Data Availability” section).

CSEP is a global community of scientists dedicated to advancing earthquake predictability research through the prospective evaluation of seismicity forecasts in fully transparent and reproducible experiments, in which strict definitions of the modeling parameters and evaluation datasets are agreed upon in advance to avoid common biases. Based on these criteria, models providing probabilistic estimates of seismicity in temporal, spatial, and magnitude domains are evaluated against future, yet-to-be-collected data of earthquakes^4^. One of CSEP’s major community accomplishments is the development and automation of 27 time-varying, spatially inhomogeneous seismicity models, which between August 1, 2007, and August 30, 2018, generated a fully prospective database of 51,625 next-day *M*_w_ ≥  3.95 earthquake forecasts for California^7^. Each of these forecasts was issued at midnight (UTC), covering a 24-h time horizon and using earthquakes observed before each start time as input (see Fig. 1, Supplementary Fig. 2, Supplementary Data/Tables 1 and 2, and the companion data publication^7^ to this article for more details).

This extensive set of increasingly complex earthquake forecasting models was gradually put into operation (see Supplementary Fig. 1) and included: (i) different flavors of the well-established Epidemic-Type Aftershock Sequence (ETAS)^5,8^ and Short-Term Earthquake Probability (STEP)^9^ models, which combine empirical statistical descriptions of past clustered seismicity, including the Omori–Utsu time-decay law^10^ and the Gutenberg–Richter frequency-magnitude relation^11^, to forecast background and triggered seismicity; (ii) versions of “alternative” models to ETAS and STEP, such as the Proximity to Past Earthquakes (PPE)^12^ and Every Earthquake a Precursor According to Scale (EEPAS)^13^ models (e.g., PPE-BACKGROUND-M2 and DR-EEPAS, respectively); (iii) nonparametric models or models that do not require a predetermined relationship between their parameters (e.g., GSF-ANISOTROPIC^14^ and HW-KERNEL-M2^15^) and; (iv) ensemble models or combinations of individual models (e.g., ETAS-HW-KERNEL-M2 and ONEDAY-BAYESIAN).

Currently, ETAS and STEP are the main types of clustering models used by agencies such as the National Institute of Geophysics and Volcanology (INGV) in Italy, Earth Sciences New Zealand, and the United States Geological Survey (USGS) for Operational Earthquake Forecasting (OEF) systems, which collect and disseminate authoritative information on the time dependence of seismic hazards in near-real time^16^. OEF models often forecast over different time windows (e.g., weeks, months, and years), which can be useful for various users but makes their evaluation and direct comparison with other models difficult^17^. Therefore, a comprehensive evaluation of this curated, prospective database of next-day earthquake forecasts is of paramount importance for addressing both practical and fundamental scientific questions, such as: How well can the underlying models describe the spatiotemporal evolution of earthquake sequences? Are they capable of adequately forecasting background and triggered seismicity over extended periods? What level of predictability should OEF users expect from these models?

**Fig. 2:**
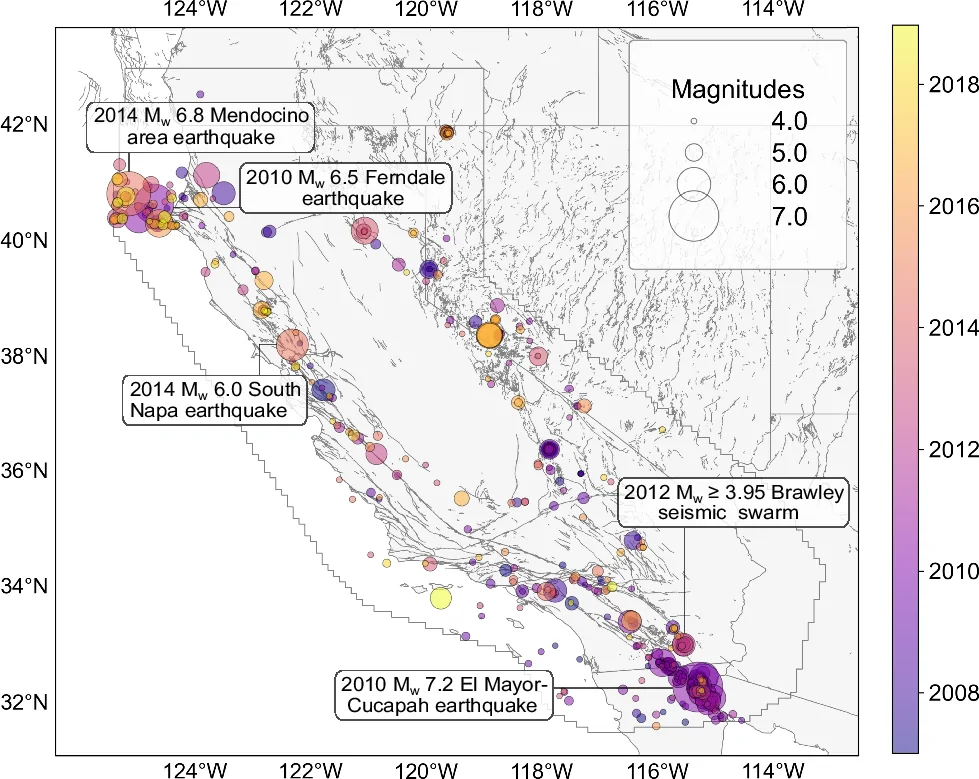
Spatiotemporal distribution of 597 earthquakes with magnitudes *M*_w_ ≥ 3.95 and depths ≤ 30 km observed in the CSEP-California testing region (gray solid polygon) between August 1, 2007, and August 30, 2018. During this period, the evaluated models prospectively generated next-day *M*_w_ ≥ 3.95 seismicity forecasts for California. This evaluation dataset includes the 2010 *M*_w_ 6.5 Ferndale, 2010 *M*_w_ 7.2 El Mayor–Cucapah, 2014 *M*_w_ 6.8 Mendocino, and 2014 *M*_w_ 6.0 Napa earthquake sequences, as well as the 2012 *M*_w_ ≥ 3.95 Brawley seismic swarm.

During the 11 years in which the models were operating prospectively, the Comprehensive Earthquake Catalog (ComCat) of the Advanced National Seismic System (ANSS)^18^ reported 597 *M*_w_ ≥ 3.95 earthquakes with hypocentral depths ≤ 30 km in the CSEP-California test region^19^, including the 2010 *M*_w_ 7.2 El Mayor–Cucapah earthquake sequence^20^, the 2012 *M*_w_ ≥ 3.95 Brawley seismic swarm^21^, and the 2014 *M*_w_ 6.0 South Napa earthquake^22^ (see Fig. 2). In this work, we prospectively evaluate, daily and cumulatively, the ability of these models to forecast the number, epicentral locations, and magnitude distribution of observed earthquakes, using statistical tests developed by CSEP and reviewed by the broader scientific community^19,23^ (see the “Methods” section). Furthermore, we quantitatively compare their long-term performance with that of a time-invariant smoothed seismicity model, hereafter referred to as HKJ-ADAPTIVE-M2^24^, which over 15 years has outperformed other time-invariant models that forecast moderate-to-large earthquakes in California^25^ and can therefore help quantify how much predictive skill is gained by time-varying models. We focus on a model group of 21 *M*_w_ ≥ 3.95 seismicity models because abundant data are available for testing and because OEF systems forecast earthquakes at this magnitude threshold. Nonetheless, prospective test results for a subset of 6 *M*_w_ ≥ 4.95 earthquake forecasting models can also be found in the Supplementary Material for this article.

We show that, overall, approximately 95%, two-thirds, and 95% of the models produced next-day forecasts consistent with the number, locations, and magnitudes of observed earthquakes, respectively. At the individual model level, select versions of ETAS and STEP demonstrated statistical agreement with prospective observations, supporting the use of this type of triggering models in public OEF.

## Results

### How good were the models at forecasting the cumulative number of observed earthquakes?

**Fig. 3:**
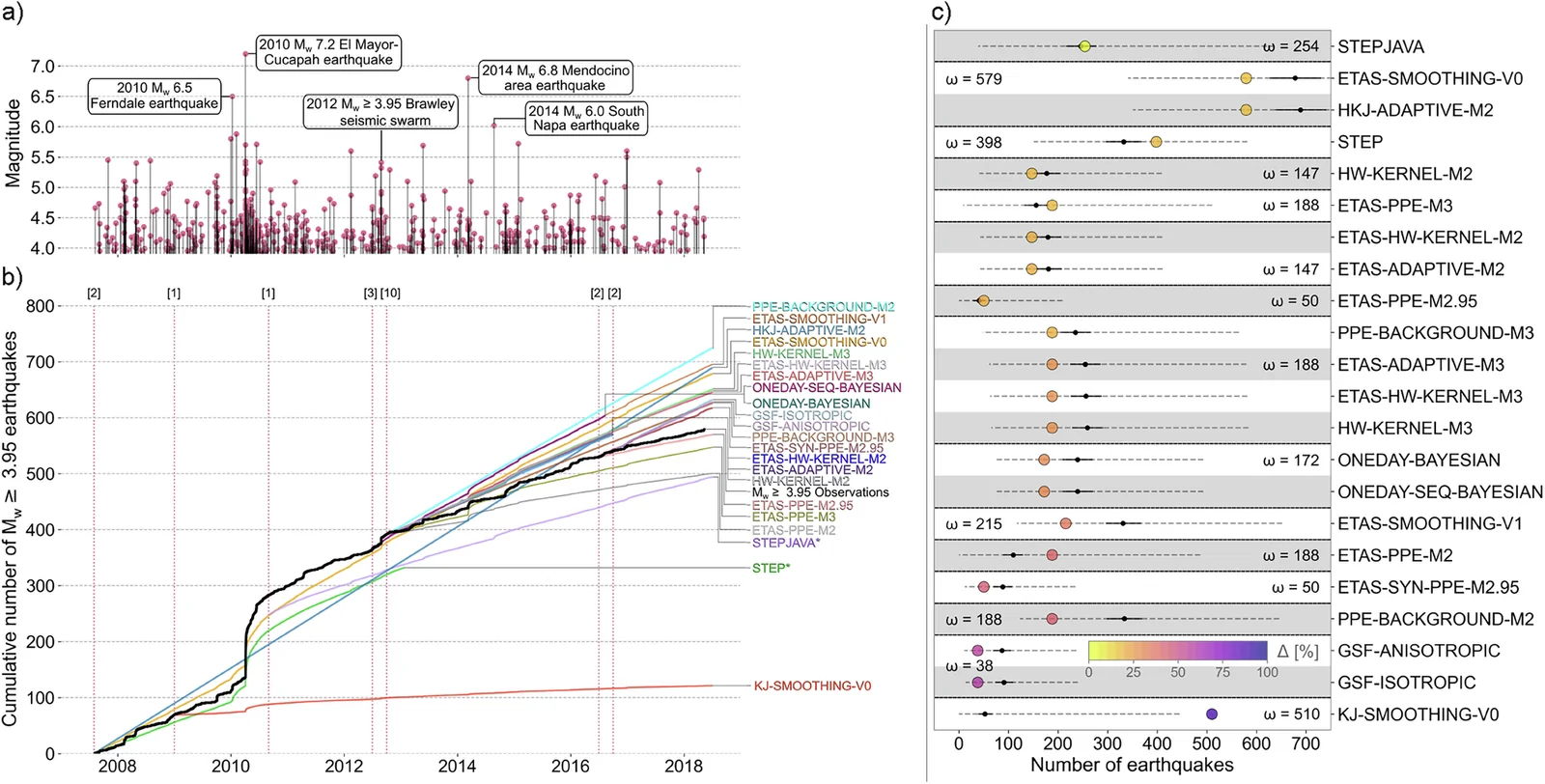
Prospective results of cumulative number tests for next-day *M*_w_ ≥ 3.95 earthquake forecasting models for California. **a** Magnitude-time series of observed *M*_w_ ≥ 3.95 seismicity in California between August 2007 and August 2018. **b** Cumulative distributions of observed and expected target earthquakes throughout the prospective evaluation period. The vertical dashed lines indicate the dates on which new models became operational (see the specific number above each line). Label asterisks highlight versions of STEP that are defined in regions smaller than the CSEP-California test region (see Fig. 1) and should therefore not be directly compared to the number of earthquakes observed in that region (black curve). **c** Prospective results of the cumulative Poisson and Negative Binomial Distribution (NBD) number tests for the next-day seismicity models. The circles represent the number of earthquakes (*ω*) observed during the different periods in which the models were prospectively generating forecasts. The colors denote (symmetric) percentage discrepancies Δ between forecasts and observations (see the colorbar). The models are ordered from lowest to highest percentage differences. Solid black bars and dashed gray bars depict the 95% confidence intervals of the Poisson and NBD number distributions of the models, respectively, using their total number of expected earthquakes (black points) as the mean of each distribution.

Quantitative assessment of the degree of (mis)match between the number of expected and observed earthquakes over extended periods can help elucidate whether the background and triggered seismicity components of models are correctly calibrated. Hence, we assess the number (in)consistencies between forecasts and observations during the different periods in which the underlying models were operational. Prospective results of the CSEP number test^26^ show that only ETAS-PPE-M2.95 and STEPJAVA forecasts were statistically consistent with the observed data (see the proximity between the circles (observations) and the black solid bars (models’ predictive intervals) in Fig. 3c). This (in)consistency test, however, relies heavily on the assumption that the number of earthquakes can be modeled using a Poisson distribution, which depends on a single parameter and is not particularly skewed, resulting in predictive intervals that do not fully capture the spatiotemporal variability of earthquake rates due to their strong clustering nature^27^.

Acknowledging this limitation, we also perform the CSEP NBD number test, whose underlying probability distribution exhibits a larger variance than the Poisson distribution and can therefore be used to better account for earthquake-rate variability^28^. In this case, we show that, with the exception of KJ-SMOOTHING-V0, all models statistically matched the observations (see how the circles fall within the models’ 95% confidence intervals, shown as gray dashed bars, in Fig. 3c). These results represent a milestone achievement in earthquake predictability research, considering in particular that (i) the models’ parameters that account for earthquake productivity were not updated throughout the evaluation period and that (ii) all next-day forecasts were automatically generated on CSEP servers over extended periods without human intervention.

For practitioners working with (symmetric) percentage differences between forecasts and observations, we further show that 9 (almost half) of the 21 time-varying *M*_w_ ≥ 3.95 seismicity models provided earthquake counts that differed by 20% or less from the total number of observed earthquakes. Among these models are versions of STEP and ETAS, such as STEPJAVA, ETAS-SMOOTHING-V0, and STEP, which over periods of approximately 8, 11, and 6 years forecasted nearly 254, 678, and 398 *M*_w_ ≥ 3.95 earthquakes, respectively, differing by 3%, 17%, and 17% from the 246, 579, and 332 events observed during such periods, respectively (Fig. 3c). We remind the reader that the models were confronted against different numbers of earthquakes *ω*, because each one has unique operationalization start and end dates (see Supplementary Fig. 1). In this sense, (in)consistency test results based on more prospective data are more indicative of their actual predictive skill.

### How good were the models at forecasting the daily number of events observed during specific earthquake scenarios?

Evaluating the models’ ability to forecast earthquakes on a daily basis provides valuable information on how closely they can track the temporal evolution of earthquake sequences, seismic swarms, and background seismicity, and is particularly useful after large events, as triggered seismicity can negatively impact emergency protocols and inflict further damage to already weakened infrastructure^29^. We therefore analyze (in)consistencies between observed and expected daily counts during (a) the *M*_w_ 6.5 Ferndale earthquake sequence, whose largest event struck off the coast of Northern California on January 10, 2010^30^; (b) the *M*_w_ 7.2 El Mayor–Cucapah earthquake on April 4, 2010, which is the largest earthquake to hit California since the 1992 *M*_w_ 7.3 Landers earthquake^20^; (c) the Brawley seismic swarm of August 26, 2012, which has been claimed to be induced by fluid pumping into a nearby geothermal field^21^; (d) the *M*_w_ 6.8 earthquake that occurred in the Mendocino area of Northern California on March 10, 2014; (e) the *M*_w_ 6.0 South Napa earthquake of August 24, 2014, which, contrary to expectations based on the Båth’s law^31^, did not trigger any *M*_w_ ≥ 3.95 earthquakes^22,32^; and (f) a randomly-selected period of relative seismic calm in California, spanning from January 1 to 20, 2018.

**Fig. 4:**
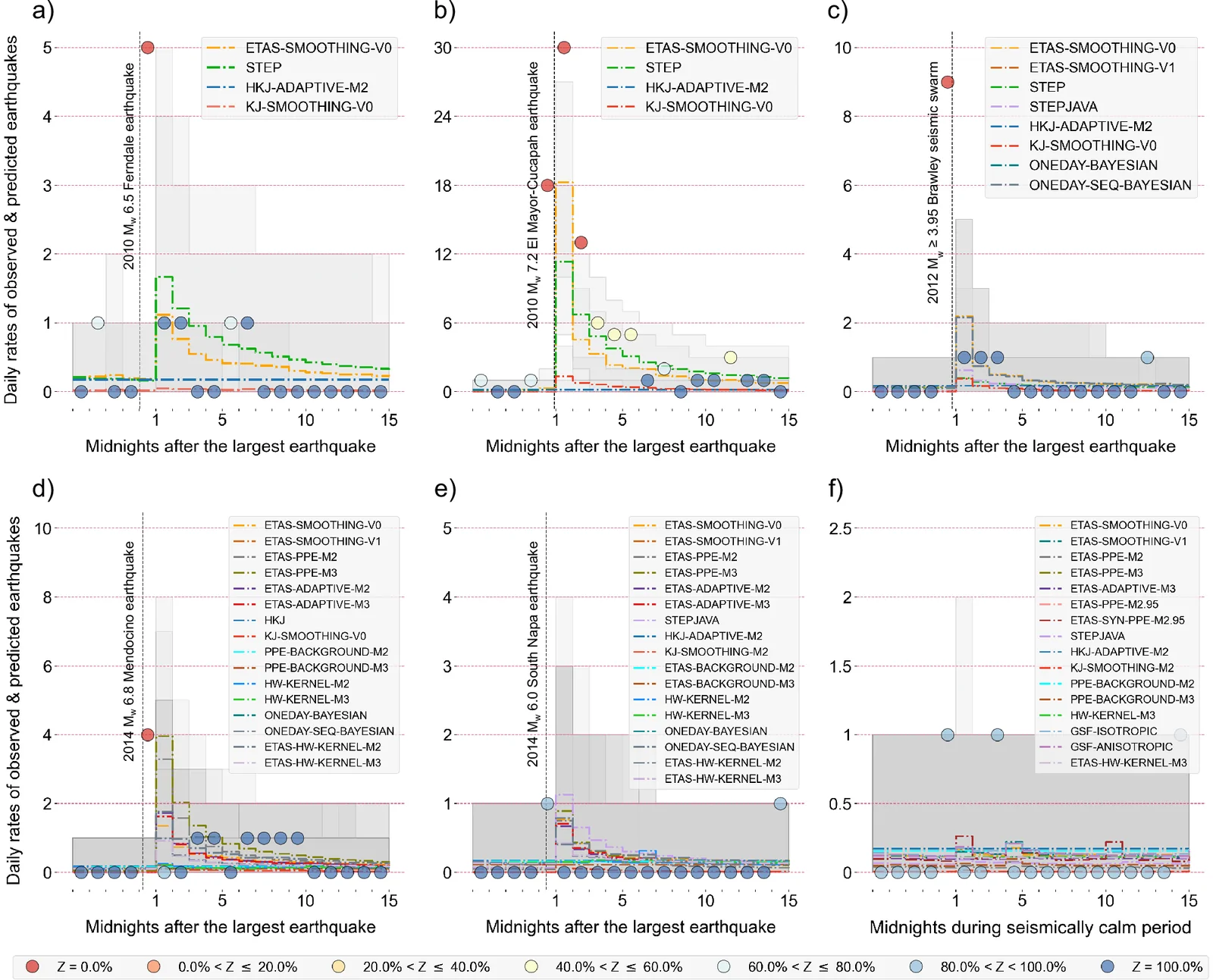
Ability of the next-day models to forecast the daily number of *M*_w_ ≥ 3.95 earthquakes (circles) observed during the first 2 weeks of specific earthquake sequences/scenarios in California. These include: **a** the 2010 *M*_w_ 6.5 Ferndale; **b** 2010 *M*_w_ 7.2 El Mayor﻿–Cucapah; **c** 2012 *M*_w_ ≥ 3.95 Brawley; **d** 2014 *M*_w_ 6.8 Mendocino; and **e** 2014 *M*_w_ 6.0 South Napa earthquake sequences, as well as **f** a randomly selected 2-week period in January 2018 of relative seismic calm in California. The vertical dashed lines denote the occurrence of the largest events in relation to specific midnights at which next-day forecasts were issued. If the daily number of observed earthquakes falls within the 95% models' confidence intervals (gray shades), observations and forecasts can be considered statistically consistent. The colors of the circles represent the percentage (*Z*) of available models that capture the observed daily counts (see the colorbar). The grayer the shades, the greater the overlap between models' confidence intervals.

All forecasts statistically underestimated the number of *M*_w_ ≥ 3.95 earthquakes observed during the days when the 2010 *M*_w_ 6.5 Ferndale, 2010 *M*_w_ 7.2 El Mayor–Cucapah, 2012 *M*_w_ ≥ 3.95 Brawley, and 2014 *M*_w_ 6.8 Mendocino earthquakes occurred (see the red circles in Fig. 4a–d). These number inconsistencies reflect the major limitations of these types of seismicity models in that their forecasts of large earthquakes (and their imminent aftershocks) will generally be of low probability during those specific days, unless the seismicity is already considerably high. In the particular case of the El Mayor–Cucapah earthquake (Fig. 4b), we note that such underestimations persisted during the first 2 days of the sequence (considering the day of the main event as day 0), until ETAS-SMOOTHING-V0 and STEP were able to adequately capture the observed daily count. Conversely, during the 2 weeks following the 2010 *M*_w_ 6.5 Ferndale earthquake, the 2012 Brawley seismic swarm, and the two 2014 *M*_w_ 6.0 and *M*_w_ 6.8 Northern California earthquakes, we find that over 80% of the models were statistically consistent with daily observations (see Fig. 4a, c, d, e).

The results for the 2012 Brawley seismic swarm (Fig. 4c) are particularly interesting because the models were not explicitly designed to forecast swarm-type seismicity, which is characterized by the lack of a single dominant earthquake and a temporal evolution that cannot be described by any simple law comparable to Omori’s law^33^. This swarm lasted one month, but produced the 9 largest (*M*_w_ ≥ 3.95) earthquakes on the first day alone, including two *M*_w_ ≥ 5.3 strike-slip events and two *M*_w_ ≥ 4.3 normal-faulting earthquakes^21^. The models, treating this swarm as a regular earthquake sequence, anticipated that the daily count would decay from a maximum of 5 to a maximum of 1 *M*_w_ ≥ 3.95 earthquake during the first 15 days of the sequence, which brought them into statistical agreement with observations. Finally, during the two randomly selected weeks of relative seismic calm in California, over 80% of the available models adequately forecasted the daily background rate, providing predictive intervals that included a number of *M*_w_ ≥ 3.95 earthquakes per day between zero and one (Fig. 4f).

### How good were the models at forecasting the spatial distribution of observed earthquakes?

Properly forecasting where earthquakes are most likely to occur can help vulnerable communities implement more targeted mitigation plans, as earthquakes, especially after major events, cluster not only temporally but also spatially. Based on this premise, we perform CSEP binary spatial tests^25^ to assess, for each model and for each of the days between August 2007 and August 2018 on which *M*_w_ ≥ 3.95 earthquakes were recorded in California, the log-likelihoods (referred to here as jbill values) of observing either no seismicity or at least one target earthquake in each of the forecasts’ spatial grid cells (see the “Methods” section). This test is summarized by the observed spatial quantile *ζ*, which is the fraction of simulated catalogs of target activated cells with spatial log-likelihoods smaller than the jbill observed on a seismically active day. If *ζ* > 0.05, then the spatial distributions of observed and expected earthquakes can be considered statistically indistinguishable.

**Fig. 5:**
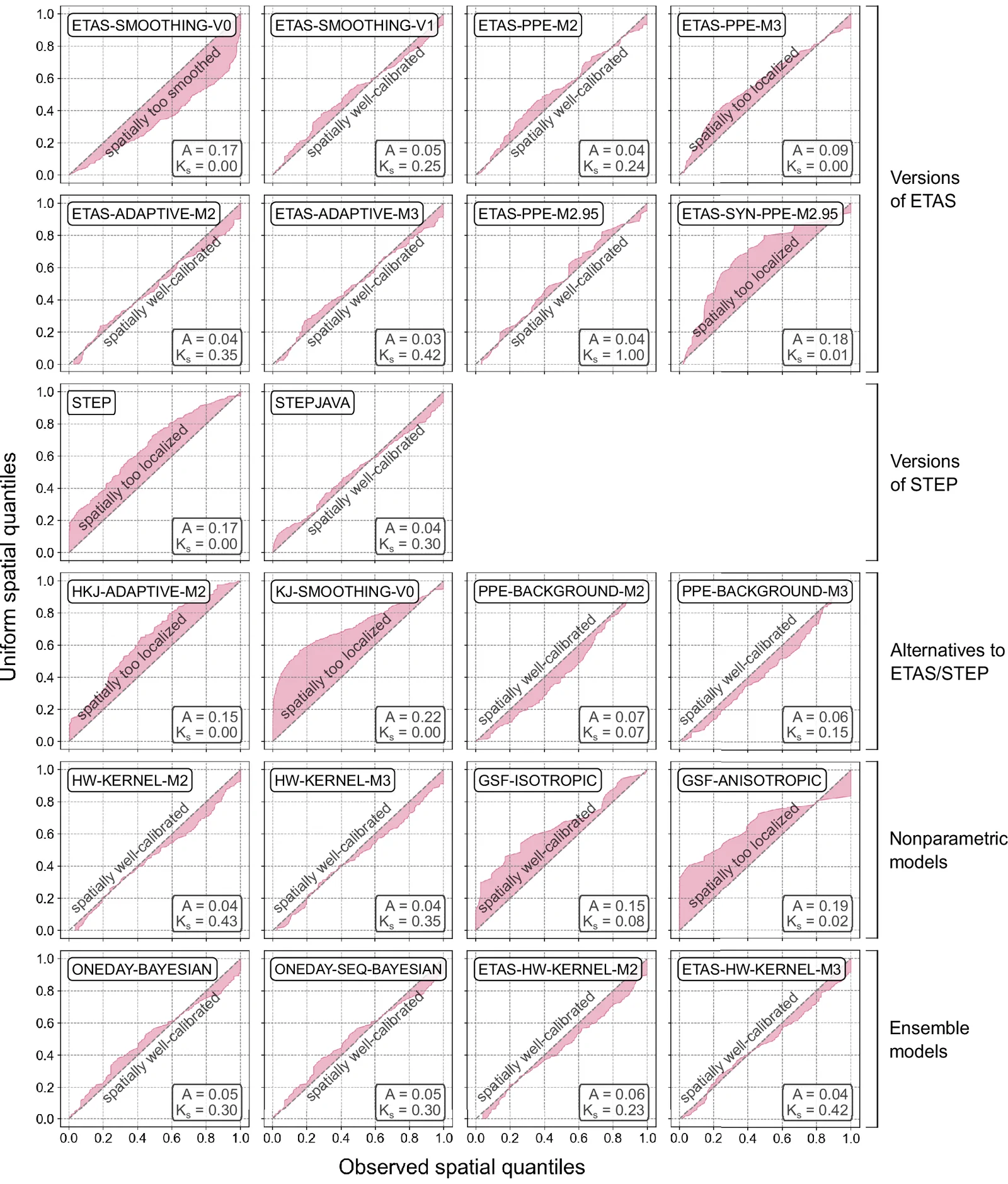
Quantile-vs-quantile plots, or so-called calibration plots, comparing the distribution of observed spatial quantiles (in ascending order) with spatial quantiles drawn from a uniform distribution. If the models were the data generator, the simulated and observed spatial quantiles should maintain a one-to-one relationship, i.e., the solid pink curves (the observed quantile distributions) should lie along the dashed gray diagonals (the uniform quantile distributions). Deviations indicate inconsistencies between forecasts and observations (see the annotated explanations). The pink shades are the annotated areas (*A*) between the diagonals and the curves. The larger the area, the greater the discrepancies between forecasts and observations. Annotated *K*_s_ values are the *p*-values of nonparametric Kolmogorov–Smirnov tests, which assess whether the (continuous) observed quantile distributions are drawn from uniform quantile distributions. If *K*_s_ > 0.05, this indicates that models generate spatial forecasts that are statistically consistent with the spatial distribution of observed earthquakes.

By sorting for each model all the observed spatial quantiles *ζ* in ascending order (pink curves in Fig. 5) and comparing these distributions with spatial quantiles drawn from uniform distributions (gray dashed diagonals), we show that 15 (~71%) of the 21 time-varying *M*_w_ ≥ 3.95 seismicity models generated next-day earthquake forecasts statistically consistent with the spatial distribution of observed epicenters (see the pink areas [*A*] between the curves and the diagonals; the smaller the area, the greater the agreement between forecasts and observations). Among the most consistent models are different flavors of ETAS and STEP, including ETAS-ADAPTIVE-M3 (*A* = 0.03), ETAS-PPE-M2 (*A*  = 0.04) and STEPJAVA (*A* = 0.04), the HW-KERNEL-M2 and HW-KERNEL-M3 nonparametric models (*A* = 0.04 in both cases), and the ensemble models resulting from combining HW-KERNEL-M3 with the ETAS-ADAPTIVE-M* sibling models, namely ETAS-HW-KERNEL-M2 (*A* = 0.06) and ETAS-ETAS-HW-KERNEL-M3 (*A* = 0.04).

**Fig. 6:**
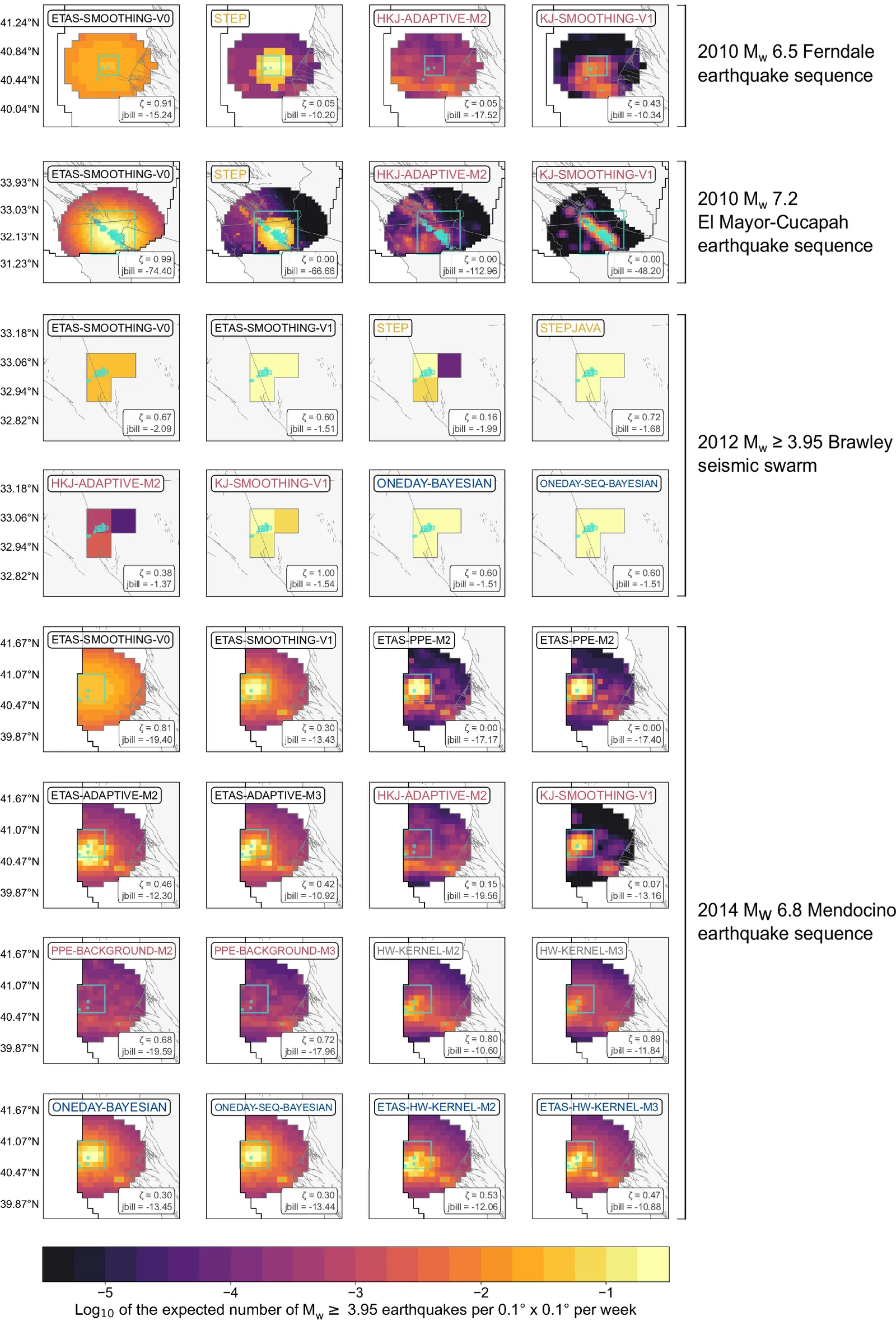
Earthquake and aftershock forecasts generated by the time-invariant HKJ-ADAPTIVE-M2 model and 17 *M*_w_ ≥ 3.95 seismicity models available during the first 7 days of four major earthquake sequences in California. These sequences are those of the 2010 *M*_w_ 6.5 Ferndale, *M*_w_ 7.2 El Mayor–Cucapah, and 2014 *M*_w_ 6.8 Mendocino earthquakes, and the 2012 *M*_w_ ≥ 3.95 Brawley seismic swarm. The spatial forecasts result from stacking 7 consecutive next-day earthquake forecasts, starting on the specific dates of the largest events. Observed earthquakes are shown as turquoise squares and expected seismicity within the aftershock zones of the larger events, defined as circular regions with radii equal to three times their average fault lengths^70^, is color-coded. Annotated “jbill” values are binary log-likelihoods of observing the data under the models. Greater jbill values indicate greater consistency with observations. If the annotated spatial quantile *ζ* is greater than 0.05, the spatial forecasts are statistically consistent with the spatial distribution of observed seismicity. The name of each model is colored according to its type: black: ETAS versions; gold: STEP versions; pink: alternative models to ETAS and STEP; blue: ensemble models; and gray: nonparametric models.

The shape and position of the pink curves relative to the gray diagonals in Fig. 5 provide additional information on the level of spatial precision that the models can offer. In particular, if the pink curves lie below the diagonals, this indicates that models generated spatially overly smoothed forecasts compared to observations, whereas if the pink curves lie above the diagonals, this denotes that models provided spatial forecasts that are too localized or inaccurate (see the “Methods” section). To illustrate this further, and to specifically evaluate spatial forecasts during major periods of activity, Fig. 6 shows *M*_w_ ≥ 3.95 seismicity forecasts generated by models available during the first week of the 2010 *M*_w_ 6.5 Ferndale, 2010 *M*_w_ 7.2 El Mayor–Cucapah, 2012 *M*_w_ ≥ 3.95 Brawley, and 2014 *M*_w_ 6.8 Mendocino earthquake sequences.

During these sequences, ETAS-SMOOTHING-V0 obtained relatively high spatial quantiles *ζ* of 0.91, 0.99, 0.67, and 0.81, respectively, mainly because the model produced overly smoothed spatial forecasts, assigning similar earthquake probabilities to wide forecast regions, while the observed seismicity was spatially more concentrated. Conversely, during the Ferndale, El Mayor–Cucapah, and Brawley sequences, STEP obtained relatively low spatial quantiles (0.05, 0.00, and 0.16, respectively), primarily because the model exhibited spatial overconfidence in forecasting that earthquakes would occur only in a few yellow spatial cells, assigning much lower probabilities elsewhere (note that the colorbar scale in Fig. 6 is logarithmic). In contrast, during the 2014 Mendocino sequence, models such as ETAS-ADAPTIVE-M2, HW-KERNEL-M2, and ETAS-HW-KERNEL-M2 obtained spatial quantiles of 0.38, 0.80, and 0.53, respectively, presumably because they adopted nonparametric components and adaptive smoothing kernels whose bandwidths vary with the level of observed seismicity, thus achieving a comparatively more effective balance between spatial precision and accuracy.

### How good were the models at forecasting the magnitudes of observed earthquakes?

**Fig. 7:**
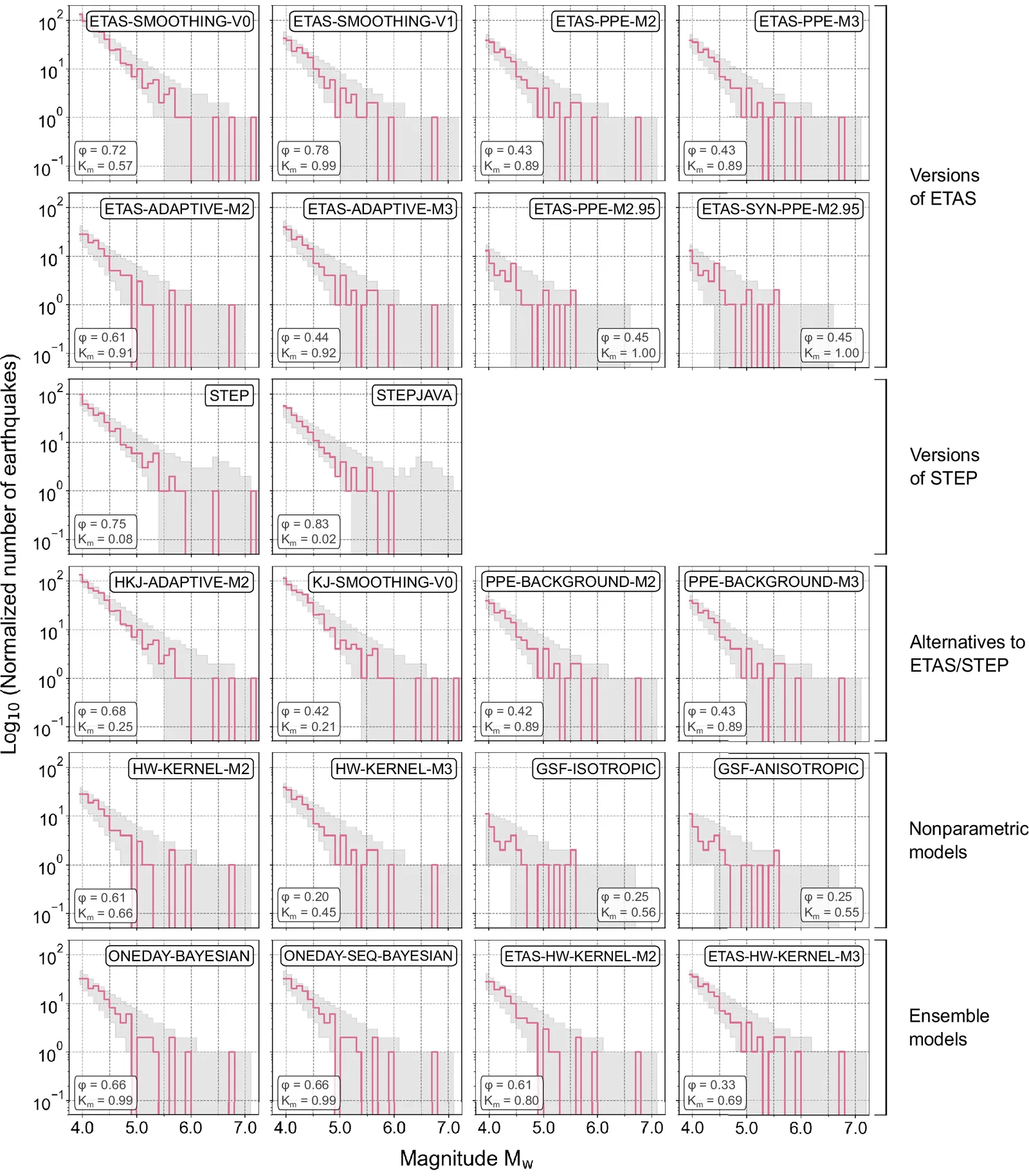
Comparisons between observed and expected frequency-magnitude distributions of *M*_w_ ≥ 3.95 earthquakes in California during the prospective evaluation periods in which the models were operational. For each pink curve, the *x*-axis shows the earthquake magnitude range divided into 0.1 unit intervals, while the *y*-axis shows the number of earthquakes observed within each magnitude bin. The models' 95% confidence intervals (gray shading) are obtained by normalizing the expected earthquake rates to the total number of observations and assuming a Poisson number distribution. Annotated *φ* and *K*_m_ values are *p*-values of the CSEP magnitude test^26^ and two-sample Kolmogorov--Smirnov tests, respectively. Models obtaining *p*-values larger than 0.05 are considered statistically consistent with the magnitude distribution of observed earthquakes.

This question is relevant from a societal and scientific point of view due to the growing interest in expected ground shaking following a large earthquake. To answer it, we quantitatively compare the incremental frequency-magnitude distribution of observed *M*_w_ ≥ 3.95 earthquakes with that predicted by models during the periods in which they were available. Prospective results of the CSEP magnitude test^26^ indicate that all models provided magnitude distributions statistically consistent with observations (see the *φ* > 0.05 values, which are the *p*-values of the test, in Fig. 7). This magnitude test, however, is limited in its ability to identify whether two data samples come from the same distribution, so we complement this analysis with nonparametric Kolmogorov–Smirnov tests (see the “Methods” section). We find that, with the exception of STEPJAVA, all time-varying models provided magnitude forecasts statistically consistent with observations, obtaining *K*_m_ values (*p*-values) greater than 0.05. Among the most consistent models are versions of ETAS, such as ETAS-SMOOTHING-V1 [*K*_m_ = 0.99], ETAS-ADAPTIVE-M3 [*K*_m_ = 0.92], and ETAS-PPE-M2 [*K*_s_ = 0.89], the ETAS-HW-KERNEL-M* ensemble models [*K*_m_ = 0.80 and *K*_m_ = 0.69, respectively], and the HW-KERNEL-M* nonparametric models [*K*_m_ = 0.66 and *K*_m_ = 0.45, respectively].

### How did each next-day model compare to the benchmark model?

In addition to quantifying the level of (dis)agreement between forecasts and observations, we compare the predictive skill of their underlying models with that of the time-invariant HKJ-ADAPTIVE-M2 model, using a measure from information theory called cumulative binary information gain per activated spatial cell (IGPA^25^; see the “Methods” section). This binary metric reduces the sensitivity of the results to the underlying test assumption that earthquakes in individual forecast cells are independent to each other and that their expected rates follow a Poisson distribution^26^.

**Fig. 8:**
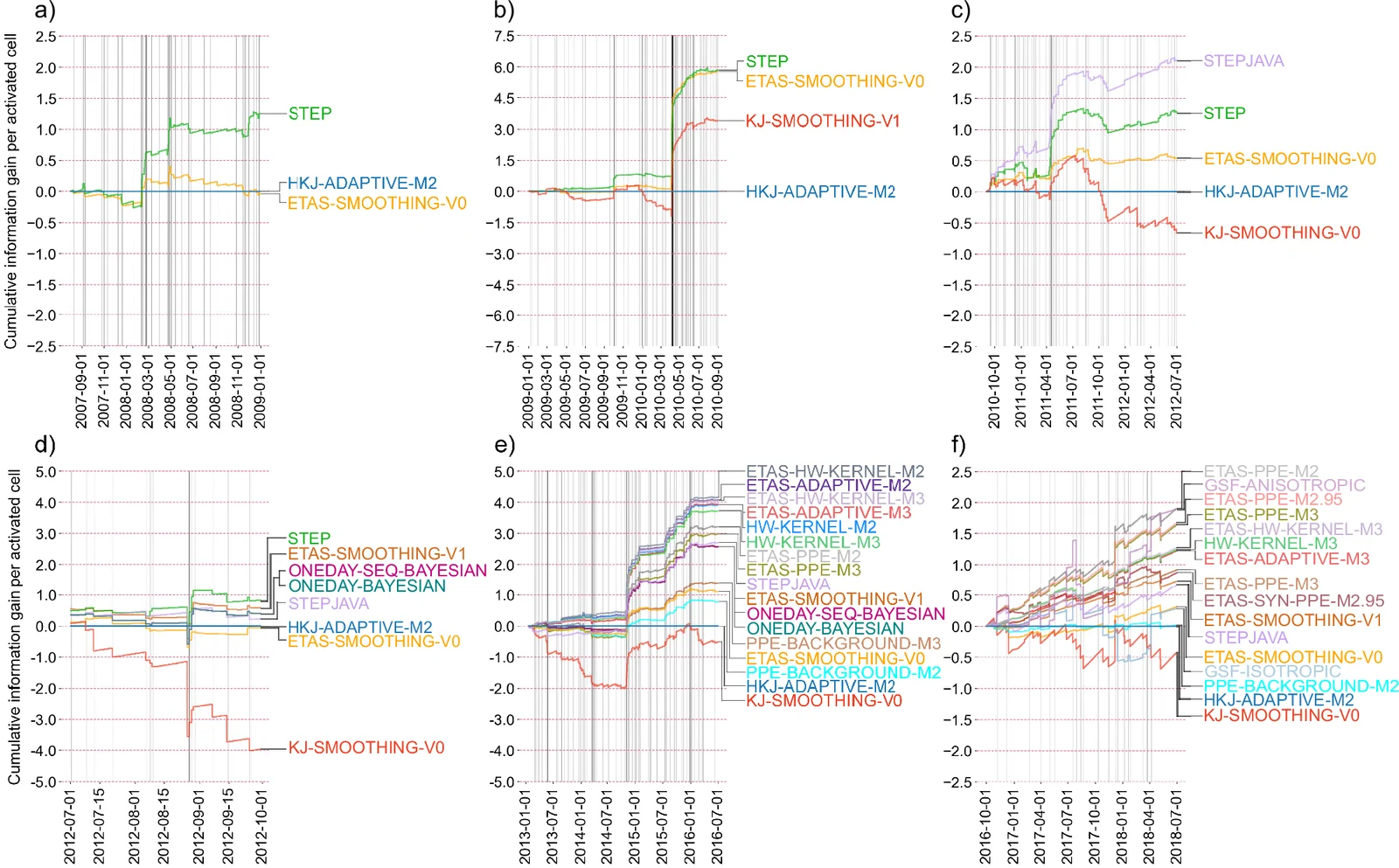
Cumulative binary information gains per activated spatial forecast cell (IGPA) obtained by each *M*_w_ ≥ 3.95 time-varying seismicity model over the time-invariant HKJ-ADAPTIVE-M2 model. Given the staggered availability of models over time, information gains are calculated across six continuous, non-overlapping time windows spanning from: **a** August 2007 to January 2009 (69 earthquakes in 47 cells); **b** January 2009 to September 2010 (214 earthquakes in 89 cells); **c** September 2010 to July 2012 (81 earthquakes in 58 cells); **d** July 2012 to October 2012 (27 earthquakes in 15 cells); **e** January 2013 to July 2016 (131 earthquakes in 81 cells); and **f** October 2016 to August 2018 (38 earthquakes in 33 cells). Within the comparatively smaller STEPJAVA test region (Fig. 1), the corresponding numbers of observed earthquakes and activated cells during the (**c**–**f**) periods are 72 and 49, 24 and 12, 108 and 65, and 33 and 28, respectively. Larger cumulative IGPA values indicate a more informative next-day model relative to HKJ-ADAPTIVE-M2. Gray vertical lines indicate the occurrence of target earthquakes within each evaluation period.

Because the forecast database was implemented gradually over time (Supplementary Fig. 1), we perform comparative analyses across six non-overlapping periods, each containing a different subset of available models. This staggered availability could influence comparisons between models, particularly with older ones, meaning that comparisons based on more prospective data better reflect the relative performance of each model. Acknowledging this limitation, we report that, between August 2007 and January 2009, when only HKJ-ADAPTIVE-M2, ETAS-SMOOTHING-V0, and STEP were available, STEP statistically outperformed the two other existing models, obtaining an IGPA of approximately 1.25 over HKJ-ADAPTIVE-M2 (see Fig. 8a). Subsequently, between January 2009 and September 2010, when the 2010 *M*_w_ 6.5 Ferndale and *M*_w_ 7.2 El Mayor–Cucapah earthquakes occurred and the KJ-SMOOTHING-V0 model came into operation, STEP and ETAS-SMOOTHING-V0 were the most informative, as both obtained IGPAs of nearly 5.85 over the HKJ-ADAPTIVE-M2 model (Fig. 8b). Note that, while STEP was more skilled than ETAS-SMOOTHING-V0 at forecasting the January 2010 *M*_w_ 6.5 Ferndale earthquake and its triggered events, ETAS-SMOOTHING-V0 slightly outperformed STEP during the El Mayor–Cucapah sequence in April of that year.

Later, between September 2010 and July 2012, STEPJAVA, which is an updated version of STEP written in Java, defined on a region smaller than the entire CSEP-California test region, and which had just been included in the pool of predictive models, ranked first by obtaining an IGPA of nearly 2.12 over the HKJ-ADAPTIVE-M2 model (see Fig. 8c). Conversely, KJ-SMOOTHING-V0 was the least informative model, obtaining a cumulative IGPA (or indeed loss) of about -0.66 over HKJ-ADAPTIVE-M2. Moving forward, between July and October 2012 (Fig. 8d), when the Brawley seismic swarm struck Southern California and the ETAS-SMOOTHING-V1 and ONEDAY(-SEQ)-BAYESIAN models joined the forecast experiment, STEP and ETAS-SMOOTHING-V1 obtained the largest IGPAs (approximately 0.81 and 0.58, respectively) over HKJ-ADAPTIVE-M2, while KJ-SMOOTHING-V0, in contrast, obtained the lowest gain (approximately −3.96). The IGPAs obtained by STEP and ETAS-SMOOTHING-V1 over the benchmark model are, however, statistically insignificant (see Supplementary Fig. 9d), indicating that HKJ-ADAPTIVE-M2 can be considered as informative as the time-varying models during this period.

Between January 2013 and July 2016 (Fig. 8e), when the 2014 *M*_w_ 6.8 Mendocino and *M*_w_ 6.0 South Napa earthquakes occurred, STEP was discontinued, and 10 new models were submitted to CSEP for prospective evaluation, the ETAS-ADAPTIVE-M* and HW-KERNEL-M* models, as well as their ensembles, ETAS-HW-KERNEL-M2 and ETAS-HW-KERNEL-M3, were the most informative models, with the latter two obtaining IGPAs of about 4.16 and 4.05 over HKJ-ADAPTIVE-M2, respectively. The statistically significant outperformance of ETAS-SMOOTHING-V1 over ETAS-SMOOTHING-V0 during this 3.5-year period (Supplementary Fig. 9e) is particularly interesting, because these models only differ in their spatial smoothing parameters, demonstrating that the comparatively finer smoothing procedure implemented in ETAS-SMOOTHING-V1 resulted in better earthquake forecasting. During this period, we also note a clear distinction in models’ performance following the *M*_w_ 6.8 Mendocino earthquake on March 10, 2014, with some of the newer models obtaining the largest IGPAs and some of the older ones tending to be the least informative (e.g., KJ-SMOOTHING-V0, ETAS-SMOOTHING-V0, and ONEDAY-BAYESIAN).

Finally, between October 2016 and August 2018 (Fig. 8f), when four additional models underwent prospective testing (albeit another five were discontinued, as seen in Supplementary Fig. 1), we find that two of them, ETAS-PPE-M2.95 and GSF-ANISOTROPIC, along with ETAS-PPE-M2, were the most informative, obtaining IGPAs of about 1.67, 1.90, and 1.91 over HKJ-ADAPTIVE-M2, respectively. The KJ-SMOOTHING-V0 model was once again the least informative, obtaining an IGPA of approximately −0.42 over HKJ-ADAPTIVE-M2. Despite the limited number of forecast cells activated during this period [33], there were a total of 15 different *M*_w_ ≥ 3.95 earthquake forecasting models available, making comparisons between them meaningful, particularly around their relative abilities to forecast background seismicity. As in the case of the 2013–2016 period (Fig. 8e), we find that the most recent models obtained the largest IGPAs, while older ones tended to do the opposite. Furthermore, we observe that, during the e, f periods, i.e., between January 2013 and August 2018, model versions calibrated on small earthquakes (e.g., ETAS-PPE-M2) obtained slightly larger IGPAs than their analogous models based on larger events (e.g., ETAS-PPE-M3; see Supplementary Fig. 12), suggesting that small(er) earthquakes may add predictive skill^24^.

## Discussion

### Lessons learned for the next generation of earthquake forecasting models

Prospective evaluations reveal that the 21 time-varying *M*_w_ ≥ 3.95 seismicity models, as a whole, were most effective at forecasting the number and magnitude distributions of observed earthquakes, followed by their epicentral locations. Individually, select versions of ETAS and STEP, such as ETAS-PPE-M*, ETAS-HW-KERNEL-M*, and STEPJAVA, were among the most consistent with prospective observations. Regarding the number components, we show that, based on the CSEP NBD number test^28^, 20 models statistically captured the cumulative number of observed earthquakes (see Fig. 3c). Some of the most consistent models, such as STEP(JAVA) and ETAS-SMOOTHING-V0, were calibrated on historical *M*_w_ ≥ 3.95 earthquake catalogs dating back to 1932 and 1898, respectively, while other models, such as HW-KERNEL-M*, ETAS-HW-KERNEL-M*, and ETAS-ADAPTIVE-M*, used a more recent instrumental catalog containing *M*_w_ ≥ 2.0 earthquakes (see Supplementary Table 2). In the particular cases of ETAS-PPE-M2(.95) and ETAS-PPE-M3, the models also used a time-varying (PPE) background component^13^ (see Supplementary Table 1). Taken together, these results suggest that integrating historical earthquake records with higher-resolution seismicity catalogs and assuming time-varying background components are key elements to consider when developing seismicity models consistent with the total number of observed earthquakes in California.

Over shorter timescales, we show that more than 80% of the available models properly forecasted the daily count of earthquakes observed during the 15 days following the 2010 *M*_w_ 6.5 Ferndale, 2014 *M*_w_ 6.8 Mendocino, and 2014 *M*_w_ 6.0 South Napa sequences (see Fig. 4a, d, e). However, during the first 2 days of the 2010 El Mayor–Cucapah earthquake sequence, ETAS-SMOOTHING-V0, STEP, and KJ-SMOOTHING-V0 statistically underestimated the number of observations (Fig. 4b). This could be because the models were calibrated with spatiotemporally uniform completeness magnitudes (CM column in Supplementary Table 2), which, after major earthquakes, are known to increase dramatically and bias the expected productivity^34,35^. More importantly, these results could be due to the fact that most models used mean aftershock productivity parameters (AP in Supplementary Table 2), calibrated on past earthquakes observed throughout the CSEP-California test region, which may not entirely represent the diversity of productivity patterns observed more locally across the region^32,36^. Nevertheless, a counterargument is that STEP, despite including a model component that estimates sequence-specific earthquake productivity as time progresses and data accumulate^9^, also had difficulty forecasting triggered seismicity. Hence, we conclude that the sequence was indeed more productive than expected (see Supplementary Fig. 4) and, therefore, suggest that future improvements to the number components of these models could be achieved by: (i) considering spatially variable productivities^36^; (ii) accounting for short-term catalog incompleteness issues^34^; (iii) updating daily forecasts as soon as moderate-to-large earthquakes occur^37^; and/or (iv) updating productivity parameters more regularly. Furthermore, we recommend investigating the use of productivity parameters that depend on measurable properties, such as tectonic setting, Gutenberg–Richter *b*-values, hypocentral depths, seismogenic crustal thickness, afterslip, and heat flow, as suggested by different studies^38–42^. Clearly, models incorporating these covariates will require prospective testing.

In the particular case of KJ-SMOOTHING-V0 (and its sibling model KJ-SMOOTHING-V1), we also advocate for technical reviews of the codes that generate their seismicity forecasts, as both models presented notable difficulties in statistically capturing the number of observed earthquakes. While KJ-SMOOTHING-V0 significantly underestimated the 510 *M*_w_ ≥ 3.95 earthquakes observed over a period of almost 10 years (Fig. 3), KJ-SMOOTHING-V1 significantly overestimated the number of observations, providing a long-term rate of approximately 1.6E-3 *M*_w_ ≥ 4.95 earthquakes per day per km^2^ (estimated from forecast files) that differed substantially from the rate of approximately 3.5E-7 *M*_w_ ≥  4.95 earthquakes per day per km^2^ reported for the model^43^ (see Supplementary Fig. 3). Revised versions of these models would undoubtedly honor the unparalleled contributions, legacy, and impact of Prof. Yan Kagan and Prof. Dave Jackson (KJ in KJ-SMOOTHING-V*) within and beyond the field of seismology^44^.

In the context of background rates, we encourage further research (and prospective testing) on the optimal combination/use of PPE-BACKGROUND-M2, the time-dependent background component of ETAS-PPE-M2, as the model overestimated the long-term rate of observed seismicity by ~44% (Fig. 3c). This could be because PPE-BACKGROUND-M2 was calibrated on earthquakes recorded in periods of comparatively high seismic activity in California, including the 1989 *M*_w_ 6.9 Loma Prieta, 1992 *M*_w_ 6.1, 7.3, 6.2 Joshua Tree-Landers-Big Bear, and 1999 *M*_w_ 7.1 Hector Mine earthquakes^18^ (see Supplementary Table 2, CP column). We also highlight the remarkable performance of HKJ-ADAPTIVE-M2 during the 2012 Brawley seismic swarm, as this model, like the time-varying models, was not explicitly designed to account for variations in background rates that arise from features not contained in seismicity catalogs, such as aseismic processes^45^. This swarm lasted a total of one month, although its peak activity occurred on August 22, producing 9 *M*_w_ ≥ 3.95 shocks^21^. In response, over 80% of the models adequately forecasted the (lack of) observed earthquakes during the 15 days following these events. In fact, during the period between July and October 2012, HKJ-ADAPTIVE-M2 was found to be statistically as informative as most of the next-day models (Figs. 8d and S9d), providing support for the use of these models, as a first approximation, to describe the temporal evolution of swarm-type seismicity. Undoubtedly, adding more specific swarm-type model components could improve OEF^45,46^.

Moving into the spatial domain of the models, we find that 15 of the 21 evaluated models generated earthquake forecasts consistent with the spatial distribution of observed epicenters (see Fig. 5). This is a remarkable achievement considering that none of the different spatial parameters/methods was updated throughout the evaluation period. Broadly speaking, models that incorporated nonparametric components and “intermediate” smoothing procedures, such as adaptive smoothing, produced the most consistent spatial forecasts. E.g., the HW-KERNEL-M* models^15^, which employ a power-law kernel with variable bandwidth, along with the ETAS-ADAPTIVE-M* models^47^, which assume a power-law kernel for *M* < 5.5 triggering earthquakes and an anisotropic one for *M* ≥ 5.5 events, were among the most consistent with observations. In contrast, models such as ETAS-SMOOTHING-V0 and STEP, which predominantly use fixed isotropic smoothing functions, exhibited the poorest spatial performances, producing oversmoothed and overlocalized seismicity forecasts, respectively. The spatial limitations of ETAS-SMOOTHING-V0, nonetheless, appear to have been addressed by its successor model, ETAS-SMOOTHING-V1, which uses a comparatively smaller kernel bandwidth (see Fig. 1), resulting in improved spatial forecasting (see Figs. 8e and S9e).

The use of anisotropic smoothing kernels that assign higher earthquake probabilities to regions along the strike angle of main faults is another ingredient that appears to produce spatially consistent seismicity forecasts. The ETAS-ADAPTIVE-M* models mimic this by smoothing the locations of early aftershocks (observed within 3 days) to then forecast subsequent events. Similarly, the STEP(JAVA) models are formulated to switch to a spatially anisotropic kernel once sufficient triggered seismicity is observed^9^. However, these model components require more data than were available even after the 2010 *M*_w_ 7.2 El Mayor–Cucapah earthquake, which is the largest event in the evaluation database (see the largely isotropic earthquake forecasts generated by STEP in Fig. 6). Thus, we recommend investigating new methods to improve the spatial triggering kernel in STEP, which, in addition to early triggered seismicity, could also rely on early USGS products such as ShakeMaps^48^ and finite source models^49^, geodetic information^50^ and/or preexisting fault maps^41^.

Regarding the magnitude component of the models, we show that all of them provided earthquake frequency-magnitude distributions consistent with observations, based on the CSEP magnitude test^26^ (see Fig. 7). Similarly, by performing nonparametric Kolmogorov–Smirnov tests, we demonstrate that, with the exception of STEPJAVA, all models provided magnitude distributions statistically indistinguishable from the magnitude distribution of observed earthquakes. The results for STEPJAVA are most likely due to its predicted distribution deviating from a typical Gutenberg–Richter distribution towards higher values within the 6.0–7.0 magnitude range (similar to a characteristic-type distribution) and the fact that no *M*_w_ ≥ 6.0 earthquakes occurred in the STEPJAVA region during the time the model was operational. In general, these test results suggest using tapered Gutenberg–Richter distributions with mean *b* = 1.0 and corner magnitudes = 8.0 to forecast earthquake magnitudes in California (see Supplementary Table 1 and the references therein).

In terms of the amount of information that each competing model can provide relative to the reference model, we note that model pairs calibrated on small(er) earthquakes outperformed their sibling models based on larger events (see, e.g., the performance of ETAS-ADAPTIVE-M2 [IGPA = 0.30] over ETAS-ADAPTIVE-M3 in Fig S12). Although the improvements are modest, these findings are encouraging, considering that the next generation of high-resolution seismicity catalogs, enhanced with machine learning, template matching, and other techniques, are expected to improve probabilistic earthquake forecasting, as they contain many more small earthquakes than current instrumental catalogs^51^.

The fact that some of the most recent next-day models obtained significantly larger information gains over HKJ-ADAPTIVE-M2 than older models indicates that seismologists are already producing increasingly informative earthquake forecasts. For instance, while STEP(JAVA) were the most informative models between August 2007 and October 2012 (Fig. 8a–d), more recent flavors of ETAS, such as ETAS-ADAPTIVE-M* and ETAS-PPE-M*, along with newer nonparametric models, such as HW-KERNEL-M* and GSF-ANISOTROPIC, took over between January 2013 and August 2018 (see Figs. 8e, f, and S9). This provides compelling evidence that models, whether using higher resolution data, better spatial smoothing techniques, or more sophisticated nonparametric forecasting methods, are improving over time. In terms of data acquisition, newer models could have benefited from comparatively better constraints on earthquake locations and magnitude estimates, given the modernization and expansion of the ANSS seismic network since the early 2010s^52^.

Another promising indication of increasing predictive skill over time is that the newer ETAS-HW-KERNEL-M* ensembles were among the most informative models during the 2012–2018 evaluation period, outperforming, although not always statistically significantly, their parent forecast models (see Figs. 8d–f and S9). Looking ahead, we recommend combining next-day California models by assigning weights that maximize the skill of an ensemble as a whole rather than weighting models based on their individual skills^53^, as ensembles, such as ONEDAY-(SEQ-)BAYESIAN, which use the latter Bayesian technique, were unable to outperform their individual model components (compare them against ETAS-SMOOTHING-V1 in Fig. 8d, e).

Indeed, time-invariant ensemble models have been shown to underperform their parent models in fully prospective evaluations in California^25^. However, on a global scale, contrasting prospective test results have also been reported, with ensemble models, such as the Global Earthquake Activity Rate (GEAR1)^54^ model, outperforming its two individual model components based separately on smoothed seismicity and interseismic strain data^55,56^. In this context, we also recommend combining (and prospectively testing) next-day models for California that use a broader range of geophysical datasets, including fault-kinematic data, geodetic information, and historical/paleoseismological earthquake records^57^. Furthermore, we encourage prospective comparisons between statistical models and models that incorporate alternative (physical) descriptions of earthquake nucleation, including those based on elasto-static stress transfer estimates and rate-and-state friction laws^58^, multicycle earthquake simulations^59^, and neural point processes^60^.

### Implications for operational earthquake forecasting

When discussing the implications of this model evaluation for OEF, it is worth reminding the reader that ETAS and STEP are the main types of clustering models used for this purpose in countries such as Italy, New Zealand, and the United States. The OEF system at INGV in Italy issues probabilistic aftershock forecasts based on an ensemble model that combines, in a Bayesian fashion, local versions of ETAS, STEP, and the Epidemic-Type Earthquake Sequence (ETES) model^17^. To our knowledge, this is the only OEF system in the world that, since 2013, has been producing probabilistic local magnitude *M*_L_ ≥ 4.0 earthquake forecasts every day at midnight for the week following and immediately after the occurrence of *M*_L_ ≥ 3.5 earthquakes^37^. Prospective evaluations of daily and weekly forecasts generated by these models over the past decade have shown good overall agreement between observed and forecasted seismicity, with the exceptions of the 2012 *M*_w_ 6.1 Emilia and 2016–2017 Amatrice-Norcia *M*_w_ 6.6 earthquake sequences.

During the Emilia sequence, STEP had difficulty forecasting the spatial distribution of observed earthquakes, mainly because the model, as also noted in this work, provided spatial forecasts that were too localized compared to observations^61^. In the case of the Amatrice–Norcia sequence, the OEF-Italy models underestimated the number of observed earthquakes, presumably due to short-term catalog incompleteness issues arising during the first days after the largest events^35^. Nonetheless, in practice, this aspect was immediately mitigated by applying an empirical correction to the forecasts produced by these models^62^. Thus, based on these number and spatial test results and those presented here for California, we argue that future improvements to the OEF models in Italy could be achieved by: (i) developing and prospectively testing new versions of STEP that use larger smoothing distances and/or assign greater weights to their anisotropic triggering kernel components; (ii) developing and validating new flavors of ETAS that assume adaptive spatial kernels and time-dependent background rates; and (iii) updating model parameters more regularly, as these were last updated in April 2009^37^.

The OEF system at Earth Sciences New Zealand has been making time-varying seismicity forecasts available to the public since 2010 following major events, such as the 2010 *M*_w_ 7.1 Darfield, 2011 *M*_w_ 6.2 Christchurch, and 2016 *M*_w_ 7.8 Kaikōura earthquakes^63^. The underlying OEF model is an ensemble composed of: (i) short-term ETAS and STEP models (ranging from hours to weeks); (ii) a medium-term EEPAS^13^ model (ranging from months to years) and (iii) long-term models like PPE^12^ (spanning decades). Prospective evaluations of the forecasts generated by these models show that, during the 6 months following the 2010 Darfield earthquake, when the devastating *M*_w_ 6.2 Christchurch earthquake occurred, ETAS was statistically consistent with the total number of observations, while a next-day version of PPE significantly underestimated the observed earthquake count. Conversely, during the 3 months following the Kaikōura earthquake, STEP and ETAS significantly overestimated the observed earthquake rate, while PPE once again significantly underestimated the total number of observations^64^.

ETAS overforecasting following the Kaikōura earthquake has been partially attributed to magnitude recalibration discrepancies between local data acquisition systems, which biased the model parameters^17^. However, based on our test results for California, we recommend considering that the use of a time-invariant background component in this version of ETAS may also explain these number inconsistencies. Regarding the PPE model, our test results differ from those reported for New Zealand, as the ETAS-BACKGROUND-M2 model, a version of PPE for California, overestimated the cumulative number of observed earthquakes by 44% (Fig. 3). Thus, we recommend further prospective evaluations of all the New Zealand OEF models using the pyCSEP toolkit^19,23^ and the newly developed floatCSEP platform^65^.

Lastly, OEF in the United States is provided by the USGS and consists of an automated system for earthquakes in U.S. states and territories and a manual system for particularly complex domestic sequences and international earthquakes with estimated fatalities exceeding 100^17^. The automated system is based on the Reasenberg and Jones model, which combines the Omori–Utsu law with the Gutenberg–Richter relation and treats all aftershocks as “offsprings” of the same mainshock^6^. One limitation of this model is that it does not consider that aftershocks can also trigger other earthquakes, thereby neglecting the contribution of higher-order triggered seismicity to the total earthquake rate. Therefore, the USGS is currently developing a new version of ETAS based on regionalized earthquake parameters to underpin this automated system. The manual system, in contrast, relies on a global version of ETAS that accounts for higher-order aftershock activity, in addition to aleatory variability in earthquake productivity and time-dependent catalog incompleteness issues. This model, however, lacks a background rate component, which could lead to an underestimation of long-term earthquake hazard, particularly in regions of high seismic activity^17^.

In the particular case of California, the USGS also operates the UCERF3-ETAS model, which combines ETAS with a long-term model component that provides earthquake probabilities on faults based on elastic rebound statistics^66^. UCERF3-ETAS can be run with or without modeled faults, but given its high computational cost, the model has only been used on demand following notable earthquakes in California, such as the 2019 *M*_w_ 7.1 Ridgecrest sequence^67^. To support ongoing OEF developments in the U.S., we recommend: (i) calibrating the new ETAS model for the automated system on increasingly small seismicity; (ii) considering adaptive/nonparametric spatial methods and time-dependent background components; (iii) quantitatively comparing the performance of the new ETAS model with that of an existing ETAS version whose parameters are regularly updated (for the sake of computational efficiency); (iv) comparing the global ETAS model with other global forecast models^54^; and (v) pseudoprospectively comparing the performance of UCERF3-ETAS with that of the next-day forecast models.

The prospective test results and recommendations discussed in this work are intended to be of practical use in improving future probabilistic earthquake forecasts and supporting OEF initiatives worldwide^17^. To contribute to these efforts, we accompany this manuscript with a data publication that describes in detail the underlying assumptions and features of all the forecasting models evaluated, as well as a comprehensive tutorial on how to download and access the database of over 50,000 next-day forecasts produced by these models^7^. This extensive database, available in a standardized format, could serve as a reference for developing and rigorously testing new earthquake forecasting models. E.g., users could quantitatively compare new forecasts with this database, develop and rank novel model combinations, and design new statistical metrics to evaluate specific aspects of the models. Furthermore, we provide a peer-reviewed reproducibility software package, containing code, documentation, and data, to transparently replicate the figures, calculations, and results discussed in this work (see the “Code Availability” section).

We acknowledge that the evaluated statistical models are to some extent limited in their ability to describe seismicity, since the space-magnitude bins that compose them provide only mean values of expected earthquake distributions, which might not fully account for forecast uncertainties^27^. Therefore, we used multiple statistical metrics that minimize the resulting bias, finding that: (i) 95% of the models statistically captured the cumulative number of observed prospective earthquakes; (ii) over two-thirds of the models provided spatially consistent forecasts with observations; and (iii) 95% of the models provided incremental frequency-magnitude distributions statistically consistent with the magnitude distribution of observed seismicity. These findings mark an significant milestone in earthquake predictability research, providing compelling support for the use of this type of seismicity models in public OEF. Thus, we envision that these prospective test results, benchmarking data, and open-source software tools will help foster an earthquake culture where vulnerable communities are not necessarily concerned with “When will the next big one occur?” but rather with “How reliable will their earthquake forecasts be?”.

## Methods

### CSEP (in)consistency tests

Most next-day earthquake forecasts are defined within the CSEP-California testing region^19^ and are represented as a regular grid of 7682 cells with a spatial resolution of 0.1^∘^, each of which is subdivided into 0.1 magnitude intervals ranging from 3.95 (or 4.95 in some cases) to 8.05+. Each of the resulting 391,782 space-magnitude bins provides an average number of expected earthquakes that depends on parameters feeding each model, and earthquakes observed up to 23:59:59 of the previous day. The parameters of most of these models remained unchanged throughout the evaluation period; however, their generated forecasts updated every 24 h as data accumulated. We use the CSEP number test^26^, implemented in the pyCSEP software toolkit^19^, to quantitatively evaluate the degree of (dis)agreement between the daily and cumulative number of observed and expected earthquakes. This test assumes that forecast cells are spatially independent of each other and that their expected daily rates follow a Poisson distribution^26^. Thus, the test calculates the probability *P* of observing *ω* earthquakes, given the expected number (or rate) of earthquakes *λ*:

1 $$P(\omega | \lambda )=\frac{{\lambda }^{\omega }}{\omega !}\exp (-\lambda ).$$

Given that the sum of Poisson variables is a Poisson variable with a rate equal to the sum of rates of individual variables, the number test considers that, for each model, the daily expected forecast rate, i.e., the sum over all space-magnitude bins, is a Poisson process with expectation *N*_fore_. The same applies to the overall expected forecast rate, which is simply the sum of the daily expected number of earthquakes over all days. Based on this, the test assesses whether the observed earthquake rate, *N*_obs_, falls within the 95% confidence interval of the (two-sided) forecast distribution, using *N*_fore_ as the mean.

The underlying Poisson number distribution assumed in this (in)consistency test is known to not always be able to fully account for the observed spatiotemporal variability of earthquakes^27^. Therefore, we complement this test with the CSEP NBD number test^28^, whose underlying probability distribution exhibits a larger variance than the Poisson distribution, thereby more effectively accounting for earthquake clustering.

The probability mass function of a NBD is defined as:

2 $$p(\omega | \tau,\nu )=\frac{\Gamma (\tau+\omega )}{\Gamma (\tau )\omega !}{\nu }^{\omega }{(1-\nu )}^{\omega },$$

with *ω* = 0, 1, 2, .., being the number of observed earthquakes, *τ* > 0 and 0 ≤ *ν* ≤ 1 the shape parameters and *Γ* the Gamma function. The mean *μ* and variance *σ*^2^ of a NBD can be expressed as:

3 $$\mu=\tau \frac{1-\nu }{\nu };{\sigma }^{2}=\frac{1-\nu }{{\nu }^{2}}.$$

Following previously established CSEP testing methods^25,28^, we use the total number of earthquakes provided by the models during the time they were available as the mean and estimated the variance from the ANSS historical catalog^18^ in non-overlapping periods consistent with the time windows in which the models were in operation. The estimated NBD variances resulted in comparatively wider 95% confidence intervals.

For forecast users working with (symmetric) percentage discrepancies between forecasts and observations, Δ, we also calculate and report these in the main text as:

4 $$\Delta=\{| {{{\rm{N}}}}_{{{\rm{fore}}}}-{{{\rm{N}}}}_{{{\rm{obs}}}}| /\max ({{{\rm{N}}}}_{{{\rm{fore}}}},{{{\rm{N}}}}_{{{\rm{obs}}}})\} \,*\,100$$

To assess (in)consistencies between the observed and forecasted spatial distributions of earthquakes, we first isolate the spatial component of the forecasts by summing, in each cell and for each model, the expected daily earthquake rate over all magnitude bins and normalizing that rate to match the number of “activated” cells, i.e., cells containing at least one earthquake. Then, we use the CSEP binary spatial test, which, compared to the CSEP Poisson spatial test^26^, is based on a Bernoulli likelihood function that calculates the probabilities of observing either 0 or ≥1 earthquakes in each forecast cell, under a model^25^. More explicitly, the probability *P*_0_ of observing *ω* = 0 earthquakes, given an expected earthquake rate *λ* and assuming a Poisson distribution (Eq. 1), is ${P}_{{{\rm{0}}}}=\exp (-\lambda )$, while the probability of observing *ω* ≥ 1 events, *P*_1_, is ${{{\rm{P}}}}_{{{\rm{1}}}}=1-{{{\rm{P}}}}_{{{\rm{0}}}}=1-\exp (-\lambda )$. For each seismically active day, i.e., when at least one target earthquake is observed, we compute the natural logarithm of these probabilities, or the binary log-likelihood score (hereafter BILL), as:

5 $${{\rm{BILL}}}={{\rm{c}}}(i)\ln (1-\exp (-\lambda (i)))+(1-{{\rm{c}}}(i))\ln (\exp (-\lambda (i))),$$

where *c*(*i*) is the indicator function *c*(*i*) = 1 if cell *i* is activated or *c*(*i*) = 0 otherwise, so that the first term represents the contribution to the log-likelihood if at least one target earthquake falls in a given forecast cell *i* with expected rate *λ* and the second term represents the contribution to the BILL if no target earthquakes are observed there, i.e., *c*(*i*) = 0. Then, we sum the calculated BILLs across all cells *i* to obtain the observed (spatial) joint log-likelihood score, jBILL, as:

6 $${{\mathrm{jBILL}}}=\mathop{\sum}\limits_{i=1}^{n}c(i){{\rm{ln}}}(1-\exp (-\lambda (i)))+(1-c(i)){{\rm{ln}}}(\exp (-\lambda (i))),$$

The greater the log-score, the more consistent the forecast will be with the prospective data. To account for forecast uncertainties, we simulate 10,000 earthquake catalogs consistent with the number of activated cells on each day. The binary spatial test is then summarized by the quantile score *ζ*, which is the fraction of simulated catalogs of the target activated cell with spatial log-likelihoods smaller than the observed jBILL. Assuming a one-sided quantile distribution, if *ζ* > 0.05, then the spatial forecast can be considered statistically consistent with the spatial distribution of earthquakes^61^.

Given that we evaluate the models over several seismically active days, we assume that, if the models were the data generators, then the distributions of observed and simulated spatial quantiles over multiple periods should be uniform. Consequently, deviations from a uniform distribution will reflect discrepancies between forecasts and observations^68^. Particularly, if the observed quantile distributions (pink curves in Fig. 5) lie above the one-to-one relationship (i.e., the uniform distribution shown in gray), this indicates a high proportion of low quantiles compared with a uniform distribution, suggesting overly localized or inaccurate spatial forecasts. Conversely, if the general trend of each curve lies below the one-to-one line, this indicates a low proportion of low quantiles compared with a uniform distribution, suggesting overly dispersed spatial forecasts (see Fig. 5). We complement these quantile-vs-quantile analyses by calculating the area (*A*) between observed and uniform quantile distributions, via numerical integration (see the links to the two versions of the reproducibility software package in the “Code Availability” section). The smaller the area, the more consistent the forecasts and observations will be. Moreover, we perform nonparametric Kolmogorov–Smirnov tests to determine whether we can reject the null hypothesis that each observed spatial quantile distribution is drawn from a uniform quantile distribution. In this case, if the *p*-value of the test, *K*_s_, is smaller than 0.05, we can conclude that a significant difference between both distributions exists.

We then use the CSEP magnitude test^26^ to evaluate the ability of the models to forecast the overall magnitude distribution of observed earthquakes. To do so, for each day, we isolate the magnitude component of the forecasts by summing their forecast rates per magnitude bin over all spatial cells and normalizing the rate to the number of observed earthquakes. Then, we estimate the overall expected magnitude distribution by summing the expected daily magnitude distributions for all the days that each model was in operation. Thus, the magnitude test evaluates whether the observed magnitude distribution falls within the 95% predictive interval of the expected distribution, which is obtained from 10,000 simulated catalogs that are consistent with the cumulative number of observations. This test is summarized by the quantile score *φ*, which is the fraction of simulated earthquake catalogs with magnitude log-likelihoods smaller than the observed magnitude statistic. We again use a significance level of 0.05 to identify (dis)agreements between forecasts and observations.

We complement these magnitude test results by calculating 95% confidence intervals of predicted earthquake rates within each magnitude bin, assuming a Poisson distribution (gray shades in Fig. 7). If the observed incremental frequency-magnitude distributions (pink curves) fall within these intervals, forecasts and observations can be considered statistically indistinguishable. Furthermore, we perform two-sample Kolmogorov–Smirnov tests to assess whether the expected and observed magnitude distributions of earthquakes come from the same underlying discrete distribution. To asymptotically calculate the *p*-value of this test, annotated as *K*_m_ in Fig. 7, we first normalize the observed and expected incremental magnitude distributions to the total number of observations *N*_obs_, as:

7 $${{\rm{Po}}}=\frac{{\rm{No}}_{{j}}}{{{\rm{N}}}_{{\mathrm{obs}}}};\quad {{\rm{P}}}{{\rm{p}}}=\frac{{{\rm{N}}}{{\rm{p}}}_{{\rm{j}}}}{{{\rm{N}}}_{{\mathrm{obs}}}},$$

where *No*_*j*_ and *Np*_*j*_ are the number of observed and predicted earthquakes within the magnitude bin *j*, given the catalog and the model, respectively. Then, we calculate the empirical cumulative distribution functions of *Po* and *Pp*, *Fo* and *Fp*, respectively, so we can compute the Kolmogorov–Smirnov statistic *D* as:

8 $$D=\max | {{\rm{Fo}}}-{{\rm{Fp}}}|$$

Based on *D*, we then calculate *K*_m_ as a series approximation given by:

9 $${{{\rm{K}}}}_{{{\rm{m}}}}\approx 2 \mathop{\sum}\limits_{k=1}^{\infty }{(-1)}^{k-1}\exp (-2{k}^{2}\cdot {z}^{2}),$$

where *z* =  $D\cdot \sqrt{{N}_{{\mathrm{obs}}}/2}$ and *k* takes values from 1 to 1000.

### CSEP comparative tests

In addition to (in)consistency analyses between forecasts and observations, our model evaluation includes quantitative comparisons between models. We assess the performance of each next-day forecasting model relative to the HKJ-ADAPTIVE-M2 benchmark by calculating, daily and cumulatively, binary information gains per activated spatial forecast cell (IGPA), which are measures of the relative amount of information about future activated cells that two competing models can provide^25^. To estimate these, we first compute the daily probabilities of observing either 0 or 1 (or more) target earthquakes in their actual space-magnitude (*i*, *j*) bins, under two competing models M*x* (with *x* = 1, 2) as:

10 $${{{\rm{BILL}}}}_{{{\rm{Mx}}}}={{\rm{c}}}(i,j)\ln (1-\exp (-{\lambda }_{{{\rm{Mx}}}}(i,j)))+(1-c(i,j))\ln (\exp (-{\lambda }_{{{\rm{Mx}}}}(i,j))).$$

Similar to the binary spatial test, *λ*_M*x*_ has previously been normalized to the number of activated space-magnitude bins. M_1_ and M_2_ denote the HKJ-ADAPTIVE-M2 benchmark and the competing next-day model, respectively. We then sum over all space-magnitude bins to obtain joint binary likelihood scores per day, jBILL_M1_ and jBILL_M2_, which we then subtract as ${{{\rm{jBILL}}}}_{{{{\rm{M}}}}_{2}}$ − jBILL_M1_ to obtain the IGPA of the competing model over the benchmark:

11 $${{\rm{IGPA}}}=\frac{1}{{M}_{{{\rm{ac}}}}}\mathop{\sum}\limits_{i=1}^{d}{{{\rm{jBILL}}}}_{{{{\rm{M}}}}_{{{\rm{2}}}}}-{{{\rm{jBILL}}}}_{{{{\rm{M}}}}_{{{\rm{1}}}}},$$

where *M*_ac_ is the total number of spatial cells activated over different evaluation periods (see Fig. 8). We then sum these gains over all days, *d*, on which forecasts provided by the two competing models are available (note that forecasts are missing for some days)^7^, regardless whether seismicity is observed or not, so that the resulting value is the cumulative binary information gain (or loss) of a competing model over HKJ-ADAPTIVE-M2. The more positive the cumulative IGPA, the better the relative performance of the competing model will be.

To assess whether IGPAs are statistically significant, we perform one-sided paired Student’s *t*-tests^69^. This comparative test evaluates whether we can reject, at a 0.05 significant level, the null hypothesis that, for each pair of models, the mean of the differences between their cumulative jBILLs is zero. To do this, we first calculate *t*-values, which are given by the ratio between the mean of the differences in jBILLs and the standard deviation of the mean (assuming a normal distribution). Thus, based on *t*-values and *n* − 1 degrees of freedom, with *n* being the number of analyzed days in each cumulative curve in Fig. 8, we finally calculate the *p*-values of the comparative test. *p* > 0.05 indicates that the competing model is statistically significantly more informative than the benchmark model.

## Supplementary information

## Data Availability

All forecast files in HDF5 format^7^, along with fully documented code and a tutorial on how to download and access the data, are available on Zenodo under a Creative Commons open-access license at https://zenodo.org/records/15076187. Earthquake forecasts generated by individual models can also be downloaded directly from the CSEP website at https://cseptesting.org/grid-based-forecasts/. The earthquake catalog used for this prospective evaluation is freely available at https://earthquake.usgs.gov/earthquakes/search/ (last accessed June 2026), while the USGS Quaternary Fault database can be found at https://www.usgs.gov/programs/earthquake-hazards/faults.

## Code availability

Both the code and tutorial for accessing the forecast database, as well as the test results discussed in this study, rely heavily on the pyCSEP software toolkit^19,23^, which is open access and features comprehensive documentation on GitHub at https://github.com/SCECcode/pycsep. The peer-reviewed version of the reproducibility software package accompanying this manuscript can be found on Ocean Code at https://codeocean.com/capsule/3685334/tree, while the non-peer-reviewed version is freely available on GitHub at https://github.com/bayonato89/reproducibility_next_day_california. If the user employs the peer-reviewed version of this software package, please cite it as: Bayona, J.A., Serafini, F., Silva, F., Iturrieta, P., Savran, W.H., Herrmann, M., Marzocchi, W., Maechling, P.J., and Werner, M.J., Select earthquake forecasting models demonstrate consistency with prospective decadal observations in California, Ocean Code, https://doi.org/10.24433/CO.0698383.v1, 2026.
