# Supplementary material for: Select earthquake forecasting models demonstrate consistency with prospective decadal observations in California
Source: Nat Commun. 2026 Aug 6;17:9444. doi: 10.1038/s41467-026-76243-7 (PMC13538450; doi:10.1038/s41467-026-76243-7)
Supplement: Supplementary file 1 [file 41467_2026_76243_MOESM1_ESM.pdf]

Supplementary material to "Select earthquake forecasting models demonstrate consistency with decadal prospective observations in California"

José A. Bayona[1,*], Francesco Serafini[1,+], Fábio Silva[2], Pablo Iturrieta[3], William H. Savran[4], Marcus Herrmann[5], Warner Marzocchi[5], Philip J. Maechling[2], and Maximilian J. Werner[1]

[1]School of Earth Sciences, University of Bristol, United Kingdom.

[2]Statewide California Earthquake Center, United States of America.

[3]Helmholtz Centre for Geosciences (GFZ Postdam), Germany.

[4]Nevada Seismological Laboratory, University of Nevada Reno, United States of America.

[5]University of Naples Federico II, Italy.

[*]Corresponding author: José A. Bayona (Email: jose.bayona@bristol.ac.uk)

[+]Now at: Global Earthquake Model (GEM) Foundation

Forecasting Model	Main Features
ETAS-SMOOTHING-V0[1]	ETAS version based on a spatially varying background rate, the Omori–Utsu law, and an isotropic power-law smoothing kernel with a fixed bandwidth proportional to the magnitude of triggering earthquakes. The model uses a standard Gutenberg–Richter law with cut-off and corner magnitudes (m_c) of 3.95 and 8.0, respectively, as well as a historical $M \geq 3.95$ earthquake catalog dating back to 1898.
ETAS-SMOOTHING-V1[1]	Updated version of ETAS-SMOOTHING-V0 based on a smaller kernel bandwidth that provides comparatively less smoothed spatial forecasts.
ETAS-PPE-M2[2]	ETAS version based on a linear combination of a Proximity to Past Earthquakes (PPE)[3] background component and an epidemic-type aftershock component. Triggered seismicity is assumed to have the same magnitude distribution as background earthquakes, and the spatial extent of each aftershock is assumed to follow a circular normal distribution with variance that increases with the magnitude of its triggering event. The model was fitted to an input catalog of earthquakes with $M \geq 3.95$ from 1932 to 1987 and $M \geq 1.95$ from 1988 to 2006, and was optimized for the period from July 1, 1997, to June 30, 2006.
ETAS-PPE-M3[2]	Model variant of ETAS-PPE-M2 that was fitted to an instrumental $M \geq 3.0$ earthquake catalog.
ETAS-PPE-M2.95[2]	Updated version of ETAS-PPE-M3, initiated upon realizing that the input catalog of the latter model was being truncated at 3.0, instead of 2.95 as originally intended.
ETAS-SYN-PPE-M2.95[2]	Model variant of ETAS-PPE-M2.95 designed to forecast all generations of aftershocks by adding the average number of second- and higher-generation aftershocks, obtained from more than 5000 synthetic catalogs, to the expected rate of first-generation aftershocks.
ETAS-ADAPTIVE-M2[4]	ETAS version that employs a time-independent, spatially variable background rate based on an adaptive smoothing kernel, the Omori–Utsu law as its temporal triggering function, an isotropic spatial triggering function for earthquakes with $M < 5.5$, and an anisotropic function for events $M \geq 5.5$ obtained by smoothing the locations of early aftershocks. The assumed magnitude distribution is a tapered Gutenberg–Richter law with a cutoff magnitude of 2.0, $m_c = 8.0$, and a *b*-value of 1.0. The model was calibrated on $M_w \geq 2.0$ earthquakes recorded between January 1, 1981 and March, 1, 2012.
ETAS-ADAPTIVE-M3[4]	Model variant of ETAS-ADAPTIVE-M2 that uses an instrumental $M_w \geq 3.0$ earthquake catalog.

STEP[5]	STEP version composed of background and aftershock components that are not combined by summation, but rather by taking the maximum between the two. The Frankel et al. model[6] is used as the background component, while the model by Reasenberg and Jones[7] is used as the aftershock temporal component. The model uses cluster-specific parameters for the space-time-magnitude distribution of aftershocks, allows these parameters to be adjusted as soon as new earthquakes occur, and was calibrated on M $\geq$ 3.95 seismicity recorded from January 1, 1932, to November 1, 2006.
STEPJAVA[5]	Variant of STEP written in Java. This is because the original version of STEP, implemented in MATLAB, became obsolete, so the Java version was installed as a replacement.
HKJ-ADAPTIVE-M2[8]	Time-invariant model that adaptively smooths the locations of M $\geq$ 2.0 earthquakes recorded from January 1, 1981, to August, 8, 2005. The model uses an isotropic, power-law smoothing kernel whose bandwidth increases or decreases if the density of seismicity at a specified location decreases or increases, respectively, thus providing high spatial resolution in seismically active regions. Furthermore, it assumes a Gutenberg–Richter magnitude distribution with m_c = 8.0 and b = 0.95, and takes into account catalog incompleteness issues.
KJ-SMOOTHING-V0[9]	Seismicity model that uses the Omori–Utsu law, an isotropic spatial triggering function, and a spatially varying background rate obtained by smoothing the locations of observed earthquake with an isotropic kernel. The assumed magnitude distribution is a standard Gutenberg–Richter law with b = 0.975 and m_c = 8.05. The model was calibrated using a historical M $\geq$ 3.95 earthquake catalog spanning from January 1, 1932 to October 2, 2008.
PPE-BACKGROUND-M2[2]	PPE background component of ETAS-PPE-M2 that smooths the locations of past earthquakes and updates daily. The model assumes a Gutenberg–Richter frequency-magnitude distribution with b = 1.0 and m_c = 8.0, a uniform background rate, and an isotropic smoothing kernel. Comparisons between ETAS-PPE-M2 and PPE-BACKGROUND-M2 will help elucidate how much information is provided by the aftershock model component of ETAS-PPE-M2.
PPE-BACKGROUND-M3[2]	PPE background component of ETAS-PPE-M3. Comparisons between ETAS-PPE-M3 and PPE-BACKGROUND-M3 will help elucidate how much information is provided by the aftershock model component of ETAS-PPE-M3.
HW-KERNEL-M2[10]	Nonparametric model that employs adaptive smoothing kernels, or kernels of variable bandwidths, to estimate the space-time-magnitude distribution of earthquakes. Unlike ETAS and STEP, this model does not assume a Omori–Utsu law or a tapered Gutenberg–Richter distribution. The model also uses spatially heterogeneous background rates and was calibrated on M $\geq$ 2.0 earthquakes recorded from January 1, 1981 to March 3, 2012.

HW-KERNEL-M3[10]	Model variant of HW-KERNEL-M2 that uses $M \geq 3.0$ earthquakes recorded between January 1, 1985, and March 3, 2012.
GSF-ISOTROPIC[11]	Seismicity model that formalizes the hypotheses that aftershocks are more likely to occur along the strike angle of the main rupture fault (estimated using prior microseismicity) and that the components of ETAS can more accurately be estimated nonparametrically. The model assumes an isotropic spatial triggering function and was calibrated with $M \geq 2.5$ earthquakes recorded between January 1, 1981, and August 23, 2005.
GSF-ANISOTROPIC[11]	Model variant of GSF-ISOTROPIC that uses an anisotropic spatial kernel function.
ETAS-HW-KERNEL-M2[10]	Ensemble model resulting from averaging space-time-magnitude earthquake rates provided by the ETAS-ADAPTIVE-M2 and HW-KERNEL-M2 models.
ETAS-HW-KERNEL-M3[10]	Ensemble model resulting from averaging space-time-magnitude earthquake rates provided by the ETAS-ADAPTIVE-M3 and HW-KERNEL-M3 models.
ONEDAY-BAYESIAN	Ensemble model that results from combining, in a Bayesian fashion, the ETAS-SMOOTHING-V0, ETAS-SMOOTHING-V1, and KJ-SMOOTHING-V0 models.
ONEDAY-SEQ-BAYESIAN	Version of ONEDAY-BAYESIAN that uses sequential updating and takes into account temporal correlations between forecasts.
KJ-SMOOTHING-V1*[9]	Long-term version of the KJ-SMOOTHING-V0 model, which can be updated when additional earthquake data become available. Like KJ-SMOOTHING-V0, this model uses a radially symmetric spatial smoothing kernel that decreases approximately as the reciprocal of the square of epicentral distance, weighted according to the magnitude of each past earthquake.
DR-EEPAS*[3]	Medium-term model that posits that smaller earthquakes act as precursors to subsequent larger events. As such, the model is based on a scaling relationship between the magnitude of precursory events and the time, area, and magnitude of future earthquakes. This model was fitted to a $M \geq 2.5$ earthquake catalog from 1932 to 1984, while its parameters were optimized using earthquakes recorded between 1985 and 2004.
DR-PPE*[2]	Long-term model that operates on the principle that future earthquakes are most likely to occur near the epicenters of past ones, adapting over time to account for clustered seismicity. The model uses an inverse power law to calculate the decrease of seismic activity with distance, a spatially uniform background rate, and a Gutenberg–Richter distribution with $b = 1.0$ and $m_c = 8.05$.
DR-ETAS*[2]	ETAS version that uses a bivariate normal distribution with circular symmetry, rather than an inverse power law, to model the spatial distributions of aftershocks.

DR-ETAS-EEPAS-PPE*[2]	Ensemble model resulting from a linear combination of the DR-PPE background model, the DR-PPE medium-term model, and the DR-ETAS short-term model. The combination assigns weights of 0.045, 0.255 and 0.7 to these models, respectively.
STEP-EEPAS*[12]	Ensemble model resulting from the optimized linear combination of STEP and DR-EEPAS. This combination, calibrated on $M \geq 5.0$ earthquakes recorded during the period 1984–2004, assigns weights of 0.58 and 0.42 to STEP and DR-EEPAS, respectively.

Supplementary Table 1. Summary of key features, parameters, and calibration datasets used by next-day $M_w \geq 3.95$ earthquake forecasting models for California. The asterisks highlight the subset of models that forecast $M_w \geq 4.95$ earthquakes.

Forecasting Model	Original Name	BR	AP	SD	BV	MM	CM	CP
ETAS-SMOOTHING-V0	ETAS	0.098	–	–	–	3.95	8.05	1898–1984
ETAS-SMOOTHING-V1	ETASV1.1	0.130	–	–	–	3.95	8.05	1898–1984
ETAS-PPE-M2	ETAS-DROneDayMd2	TD	0.057	1.0	1.0	2.0	8.0	1932–2006
ETAS-PPE-M3	ETAS-DROneDayMd3	TD	0.087	1.0	1.0	3.0	8.0	1932–2004
ETAS-PPE-M2.95	ETAS-DROneDayMd2.95	TD	0.087	1.0	1.0	2.95	8.0	1932–2004
ETAS-SYN-PPE-M2.95	ETASSYN-DROneDayMd2.95	TD	0.087	1.0	1.0	2.95	8.0	1932–2004
ETAS-ADAPTIVE-M2	ETAS-HWMd2	0.066	0.385	2.0	1.0	2.0	8.0	1981–2012
ETAS-ADAPTIVE-M3	ETAS-HWMd3	0.074	0.475	2.0	1.0	3.0	8.0	1985–2012
STEP	STEP	TD	TD	TD	TD	3.95	8.0	1932–2006
STEPJAVA	STEPJAVA	TD	TD	TD	TD	3.95	8.0	1932–2006
HKJ-SMOOTHING-M2	HKJ	–	–	0.5+	0.95	2.0	8.0	1981–2005
KJ-SMOOTHING-V0	KJSSOneDayCalifornia	0.172	–	5.0	0.975	3.95	8.05	1932–2008
PPE-BACKGROUND-M2	ETAS-DROneDayPPEMd2	TD	0.057	1.0	1.0	2.0	8.0	1932–2006
PPE-BACKGROUND-M3	ETAS-DROneDayPPEMd3	TD	0.087	1.0	1.0	3.0	8.0	1932–2004
HW-KERNEL-M2	K3Md2	AS	AS	AS	AS	2.0	AS	1981–2012
HW-KERNEL-M3	K3Md3	AS	AS	AS	AS	3.0	AS	1985–2012
GSF-ISOTROPIC	GSF-ISO	NS	0.750	1.244	NS	2.5	NS	1981–2005
GSF-ANISOTROPIC	GSF-ANISO	NS	0.750	1.244	NS	2.5	NS	1981–2005
ETAS-HW-KERNEL-M2	ETAS-HW-K3-AVERAGE-Md2	AS	AS	AS	AS	2.0	8.0	1981–2012
ETAS-HW-KERNEL-M3	ETAS-HW-K3-AVERAGE-Md3	AS	AS	AS	AS	3.0	8.0	1985–2012
ONEDAY-BAYESIAN	OneDayBayesianBMA	TD	TD	TD	TD	3.95	8.05	2007–2013
ONEDAY-SEQ-BAYESIAN	OneDayBayesianSeqBMA	TD	TD	TD	TD	3.95	8.05	2007–2013
KJ-SMOOTHING-V1*	KJSSFiveYearCalifornia	0.172	–	5.0	0.975	3.95	8.05	1932–2008
DR-EEPAS*	JANUSOneDayEEPAS1F	TD	0.072	2.43	1.0	2.95	8.05	1932–2004
DR-PPE*	JANUSOneDayPPE	TD	0.072	2.43	1.0	2.95	8.05	1932–2004
DR-ETAS*	JANUSOneDayTV	TD	0.072	2.43	1.0	2.95	8.05	1932–2004
DR-ETAS-EEPAS-PPE*	JANUSOneDay	TD	0.072	2.43	1.0	2.95	8.05	1932–2004
STEP-EEPAS*	SE2OneDay	TD	TD	1.62	0.91	4.95	8.05	1984–2004

Supplementary Table 2. Summary of key characteristics and parameters of participating forecast models. BR: Background Rate [$M_w \geq$ 3.95 EQs/day]; AP: Aftershock Productivity; SD: Smoothing Distance [km]; BV: *b*- value; MM: Minimum Calibration Magnitude; CM: Corner Magnitude; CP: Calibration Period; –: Unavailable; TD: Time Dependent; AS: Adaptive Smoothing; NS: Nonparametric Smoothing. The asterisks highlight the subset of models that forecast $M_w \geq 4.95$ earthquakes.

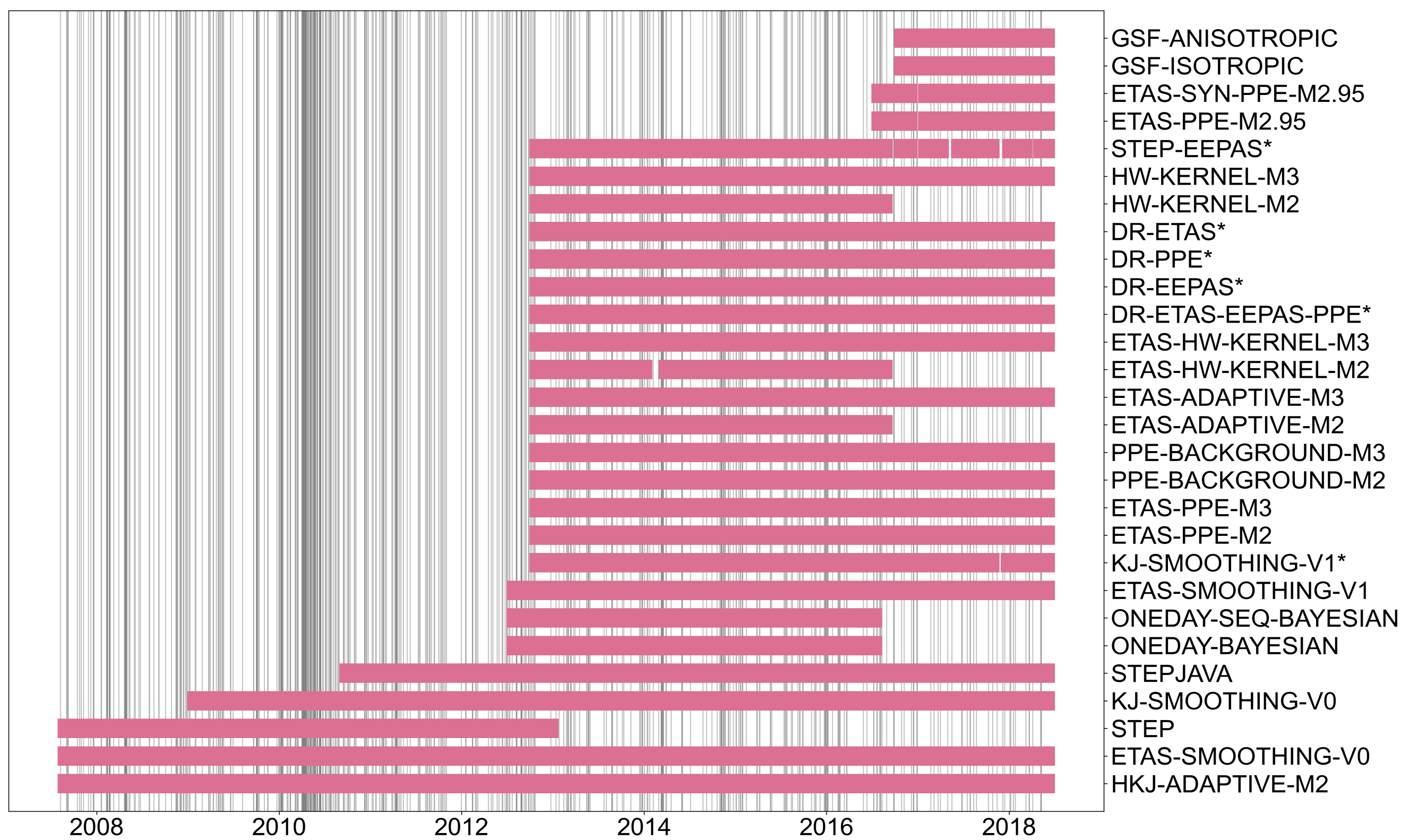

Supplementary Figure 1. Predictive pool of next-day $M_w \geq 3.95$ earthquake forecasting models for California. The availability of forecasts generated by these time-varying models and the time-invariant HKJ-ADAPTIVE-M2 benchmark model during the evaluation period, i.e., between August 1, 2007 and August 30, 2018, is shown in pink. Label asterisks denote a subset of 6 models providing $M_w \geq 4.95$ seismicity forecasts. Prospective earthquakes with moment magnitude $M_w \geq 3.95$ and depths ≤ 30 km occurring during this period are shown as vertical gray lines. The number of models increases with time due to new models being developed and submitted to CSEP for prospective testing. Some forecasts have been irretrievably lost due to system processing failures, especially since 2016. E.g., STEP was decommissioned due to software licensing issues. For more information on the exact dates of missing forecasts, see the accompanying data publication[13].

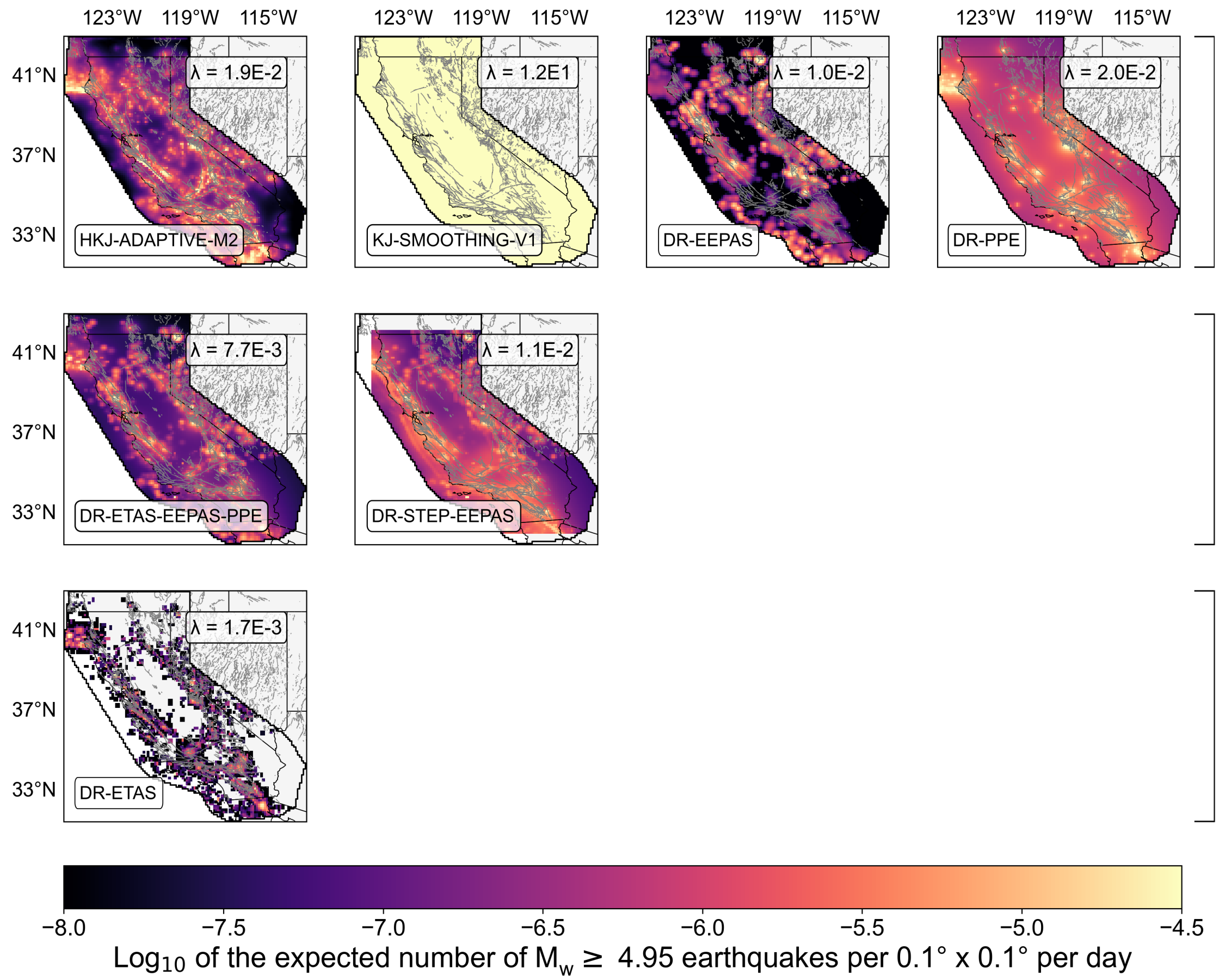

Supplementary Figure 2. Forecast maps generated by 6 $M_w \geq 4.95$ next-day seismicity models and one time-invariant model (HKJ-ADAPTIVE-M2) on August 30, 2018, at midnight, for California. This model subset includes a version of ETAS, three alternative models to ETAS and STEP, and two ensemble models. Expected rates of $M_w \geq 4.95$ seismicity are expressed per 0.1° x 0.1° cell per day, with bright colors denoting regions where seismic activity is comparatively high, dark colors indicating comparatively low rates, and white areas denoting regions where models are not defined. The annotated λ values indicate the number of $M_w \geq 4.95$ earthquakes predicted by the models during the aforementioned day. Only the HKJ-ADAPTIVE-M2 model provides daily earthquake rates that do not change over time. Fault traces (shown in gray) are obtained from the USGS Quaternary Fault and Fold Database (see the Data Availability section).

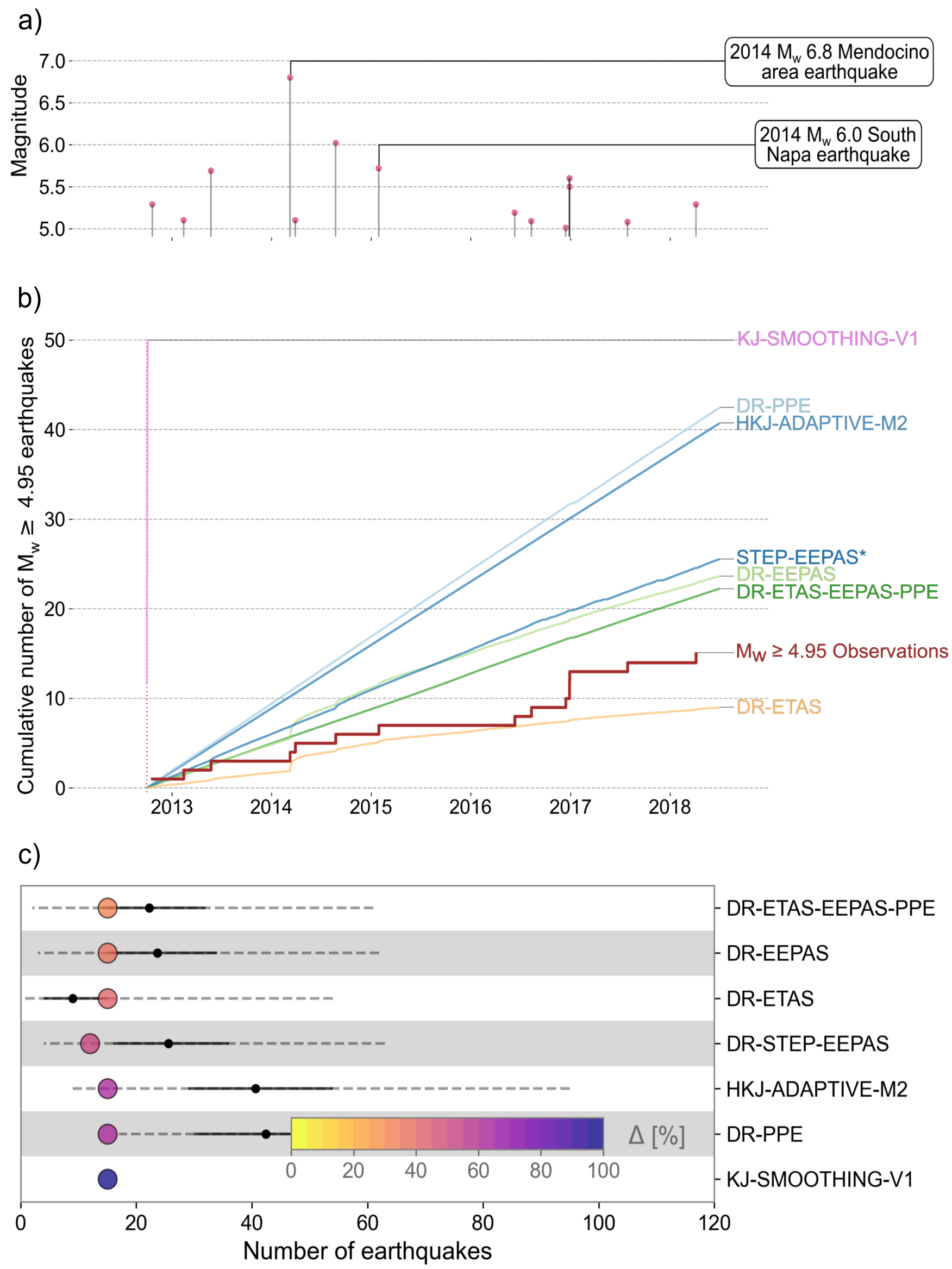

Supplementary Figure 3. a) Magnitude-time series of observed $M_w \geq 4.95$ earthquakes in California between October 2012 and August 2018. b) Cumulative distributions of observed and predicted target earthquakes during the prospective evaluation period. The vertical dashed pink indicates the date on which the models came into operation. The asterisk highlights the STEP-EEPAS ensemble model, which is defined in a region smaller than the CSEP-California testing region (see Fig. **??**) and should therefore not be directly compared to the number of earthquakes observed across the entire CSEP-California test region (brown curve). c) Prospective test results of the cumulative Poisson and Negative Binomial Distribution (NBD) number tests for next-day earthquake forecasting models for California. The circles represent the number of observed earthquakes ω and the colors denote their percentage discrepancies Δ with the number of earthquakes expected by the models(see the colorbar). Solid black bars and dashed grey bars depict the 95% predictive intervals of the Poisson and NBD number distributions of the models, respectively, using their expected number of earthquakes (black dots) as the mean of each distribution. The predictive intervals of the KJ-SMOOTHING-V1 model are too large to be shown.

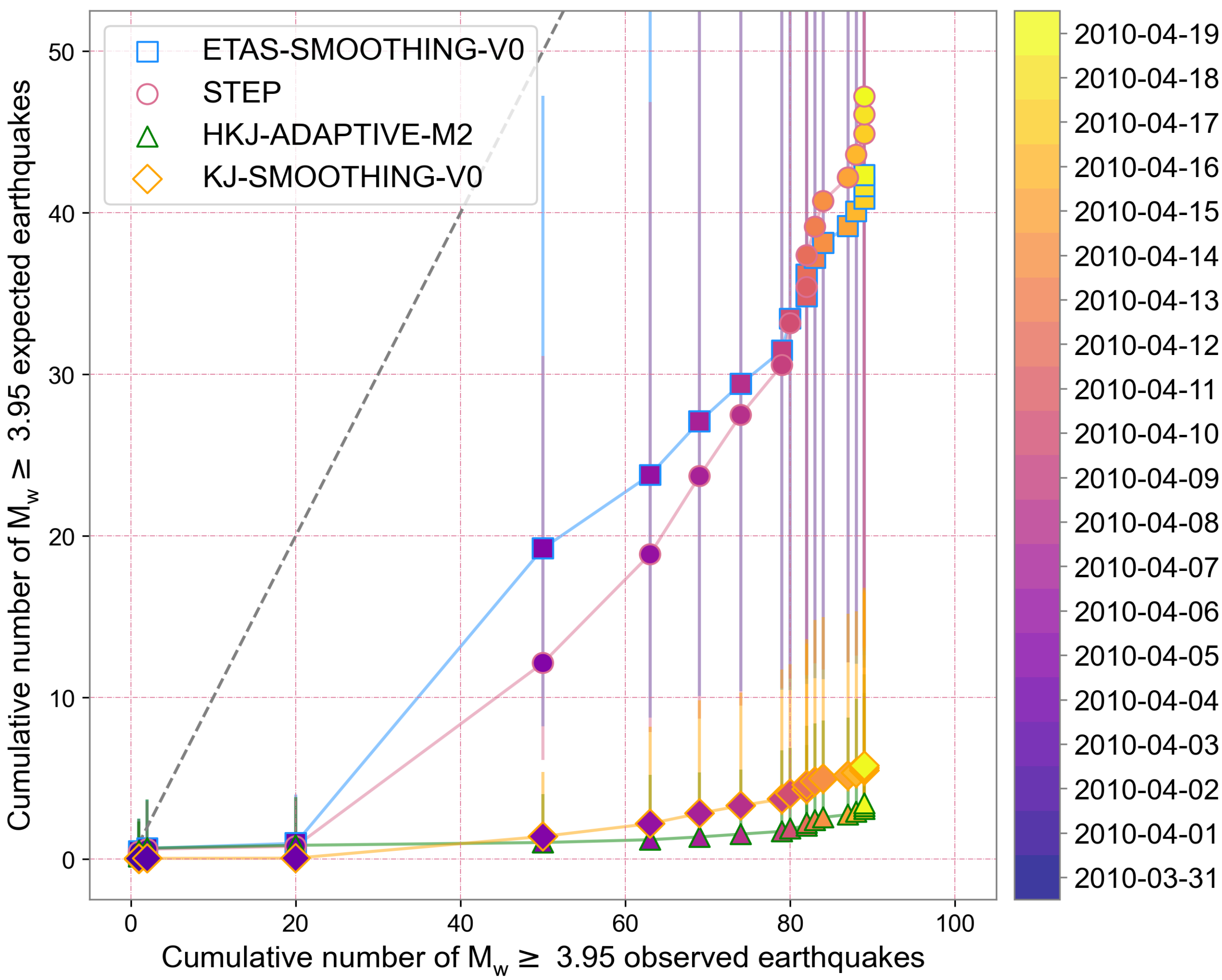

Supplementary Figure 4. Correspondence between the cumulative number of $M_w \geq 3.95$ earthquakes expected (y-axis) and observed (x--axis) during the first 15 days of the 2010 M_w 7.2 El Mayor–Cucapah earthquake sequence, whose main event occurred on April 4. The forecast models available at that time were ETAS-SMOOTHING, STEP, HKJ-ADAPTIVE-M2 and KJ-SMOOTHING-V0, each of which provided mean seismicity rates (y-coordinates of the symbols) and 95% confidence intervals (solid colored vertical lines). The dashed gray diagonal indicates a perfect one-to-one relationship between forecasts and observations.

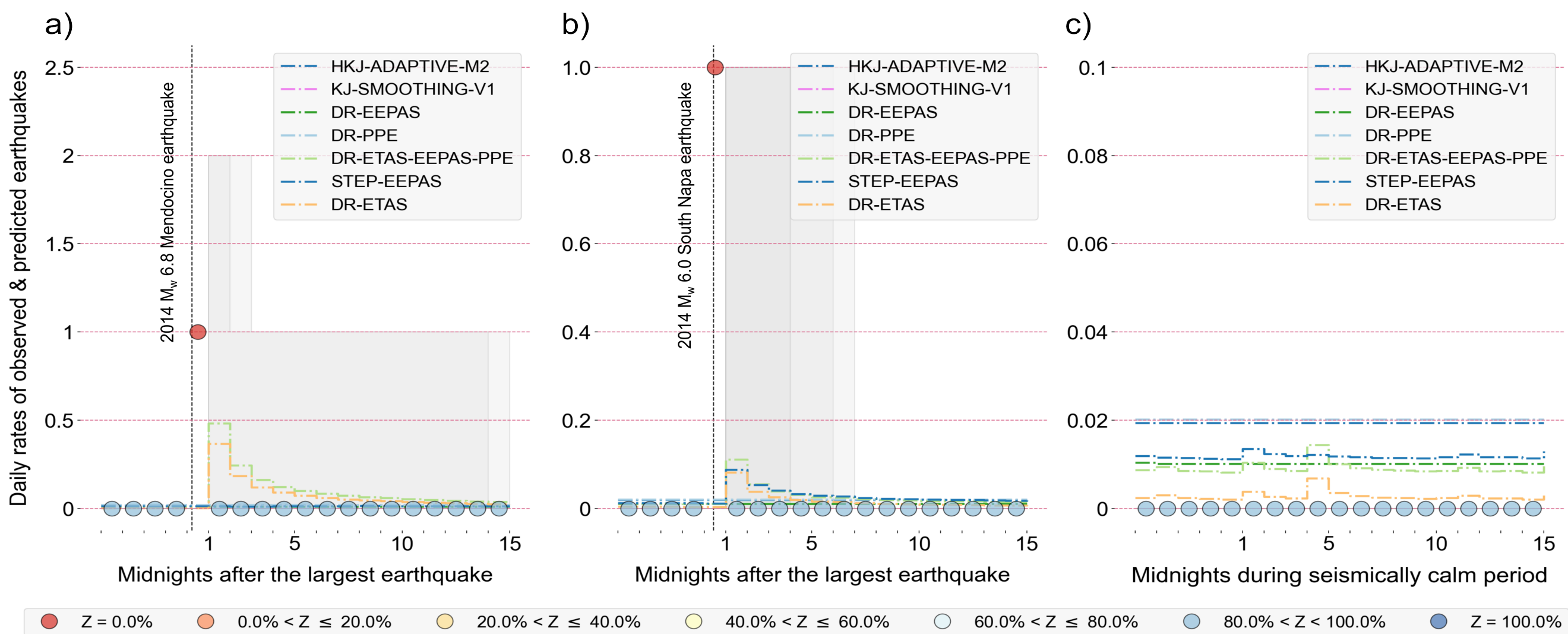

Supplementary Figure 5. Ability of next-day models to forecast the daily number of $M_w \geq 4.95$ observed earthquakes (circles) during the first weeks of a) the 2014 M_w 6.8 Mendocino and b) 2014 M_w 6.0 South Napa earthquake sequences, as well as c) during a randomly selected two-week period in January 2018 of relative seismic calm in California. The vertical dashed lines denote the occurrence of the largest events in relation to specific midnights at which next-day forecasts were issued. The colors of the circles represent the number Z of models (in percentage) that capture the observed daily counts within their 95% predictive intervals (shown in gray; see the colorbar). The grayer the shades, the greater the overlap between models' predictive intervals. The KJ-SMOOTHING-V1 model expects between 6 and 20 target earthquakes over the course of these days, so its predictive intervals are too wide to be shown. Daily expected earthquake rates in c) are so small that the assumed Poisson number distribution are skewed sharply to the right, making the predictive intervals to tend to zero (except for KJ-SMOOTHING-V1).

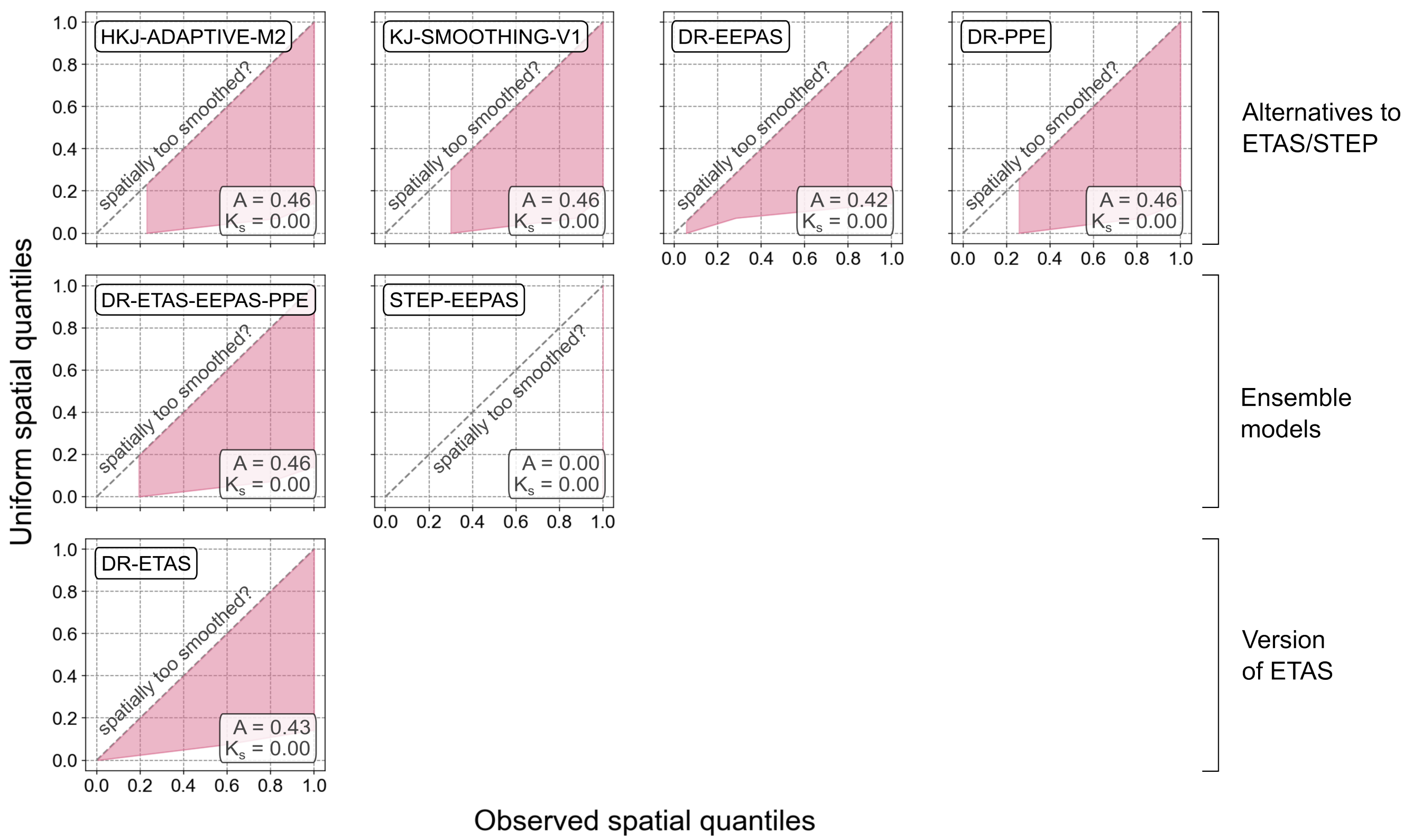

Supplementary Figure 6. Quantile-vs-quantile plots comparing the distribution of observed spatial quantiles (in ascending order) with spatial quantiles expected from a uniform distribution. If the models were the data generator, the simulated and observed spatial quantiles should maintain a one-to-one relationship, i.e., the solid pink curves (the observed quantile distributions) should lie along the dashed gray diagonals (the uniform quantile distributions). Deviations indicate inconsistencies between forecasts and observations (see the annotated explanations). Pink shades are the annotated areas (A) between the diagonals and the curves. The larger the area, the greater the discrepancies between forecasts and observations. Annotated K_s values are the *p*-values of non-parametric Kolmogorov–Smirnov tests, with $K_s < 0.05$ indicating that the models generated spatial forecasts that were statistically inconsistent with observations.

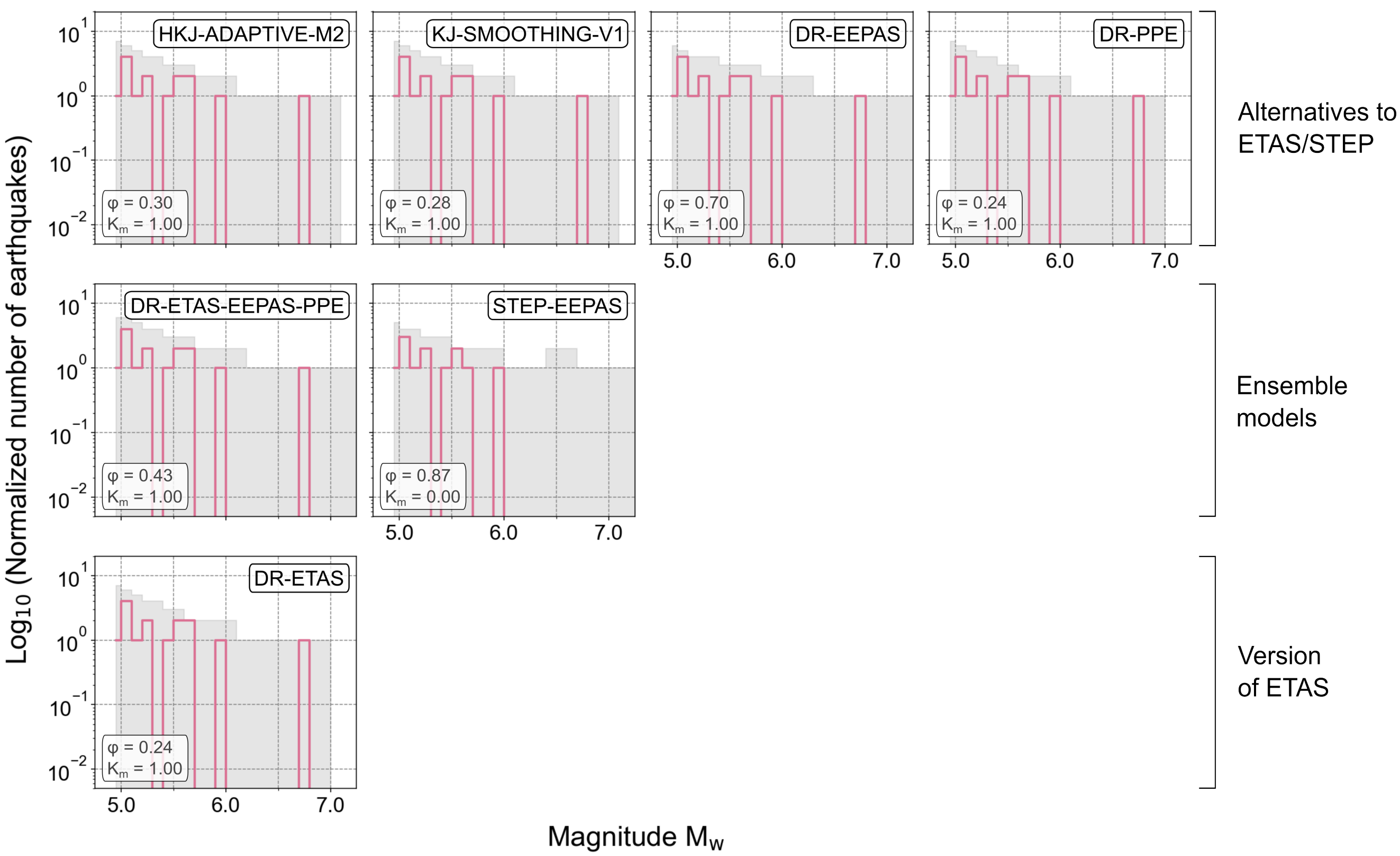

Supplementary Figure 7. Comparisons between the observed and expected frequency-magnitude distributions of $M_w \geq 4.95$ earthquakes in California. For each pink curve, the *x*-axis shows the earthquake magnitude range divided into 0.1 unit intervals, while the *y*-axis shows the number of earthquakes observed within each magnitude bin. The 95% model predictive intervals, shown as gray shading, are obtained by normalizing expected earthquake rates to the number of observed earthquakes and assuming a Poisson number distribution. Annotated φ and K_m values are *p*-values of the CSEP magnitude test[14] and two-sample Kolmogorov-Smirnov tests, respectively. Models obtaining *p*-values larger than 0.05 are considered statistically consistent with the magnitude.

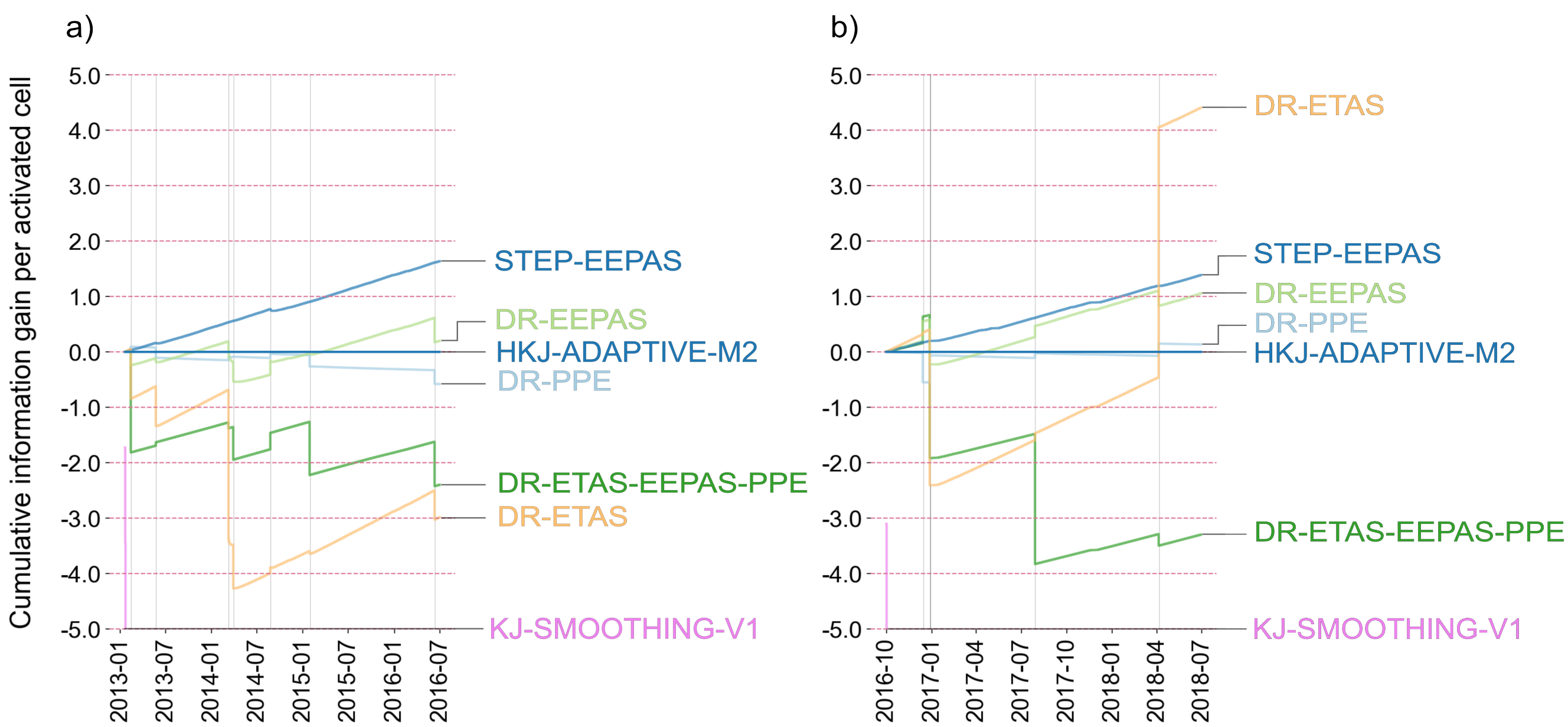

Supplementary Figure 8. Cumulative binary information gains per activated bin (IGPA) obtained by $M_w \geq 4.95$ time-varying seismicity models over the HKJ-ADAPTIVE-M2 time-invariant model between a) January 2013 and July 2016 [7 earthquakes in 7 cells] and b) October 2016 and August 2018 [6 earthquakes in 4 cells]. These gains fluctuate dramatically at the beginning of each evaluation period due to the relatively low number of space-magnitude activated bins. If the cumulative IGPA is positive, the competing model can be considered more informative than HKJ-ADAPTIVE-M2. Grey vertical lines indicate the occurrence of $M_w \geq 4.95$ earthquakes in each evaluation period.

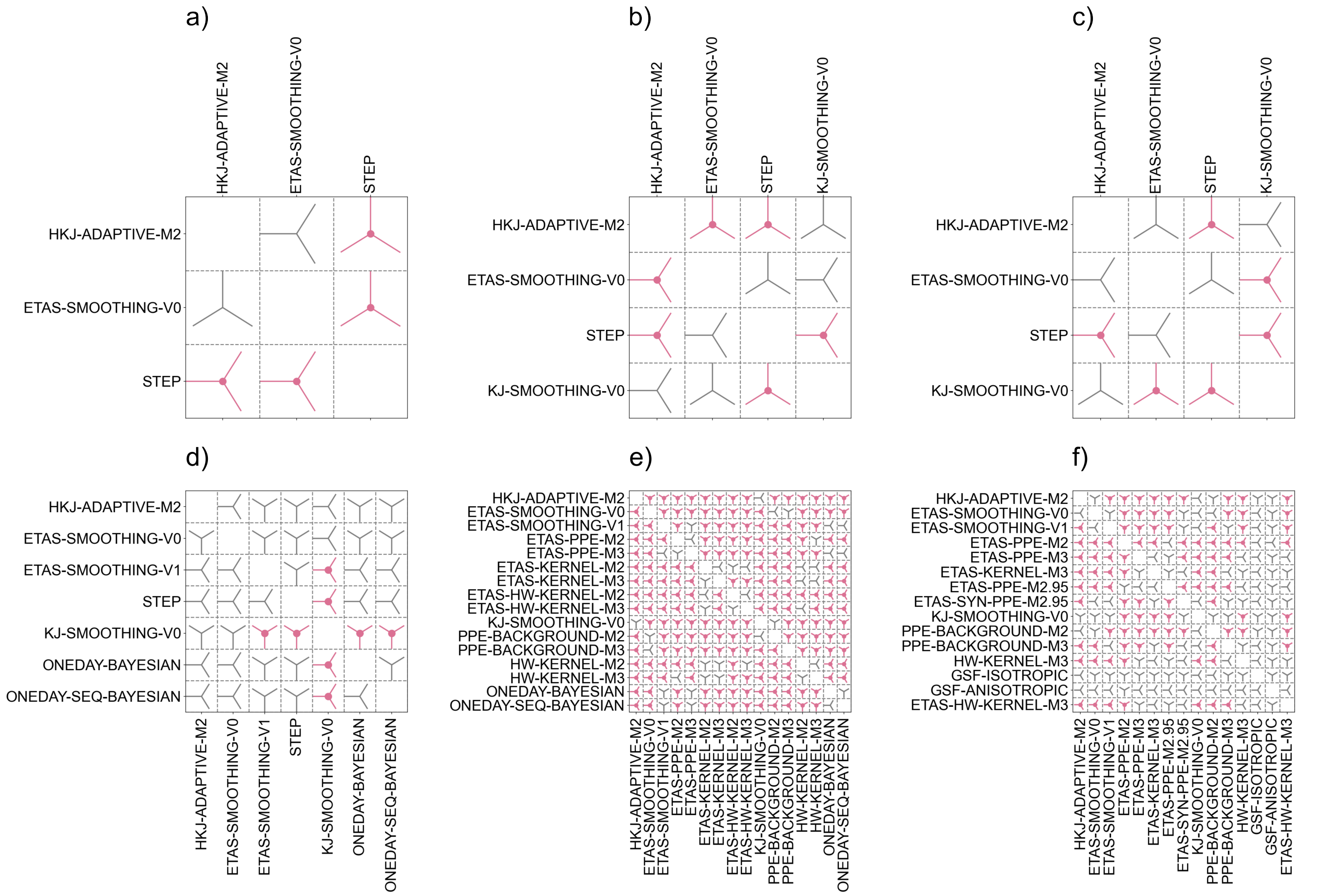

Supplementary Figure 9. Confusion matrices showing the significance of cumulative IGPAs obtained by competing $M_w \geq 3.95$ seismicity models (rows) over reference models (columns) between a) August 2007 and January 2009 [69 earthquakes; 47 activated cells], b) January 2009 and September 2010 [214 earthquakes; 89 cells], c) September 2010 and July 2012 [81 earthquakes; 58 cells], d) July 2012 and October 2012 [27 earthquakes; 15 cells], e) January 2013 and July 2016 [131 earthquakes; 81 cells], and f) October 2016 and August 2018 [38 earthquakes; 33 cells]. For each pairwise comparison, the aircraft symbol points to the most informative model. Pink aircraft symbols with midpoints indicate that the IGPA is statistically significant, i.e., the *p*-value of the comparative t-test is smaller than 0.05. Gray aircraft symbols denote the opposite. In the main text, we only discuss IGPAs obtained by next-day models over the time-invariant HKJ-ADAPTIVE-M2 model (first column of each panel). Note that all matrices are symmetric with respect to the diagonal.

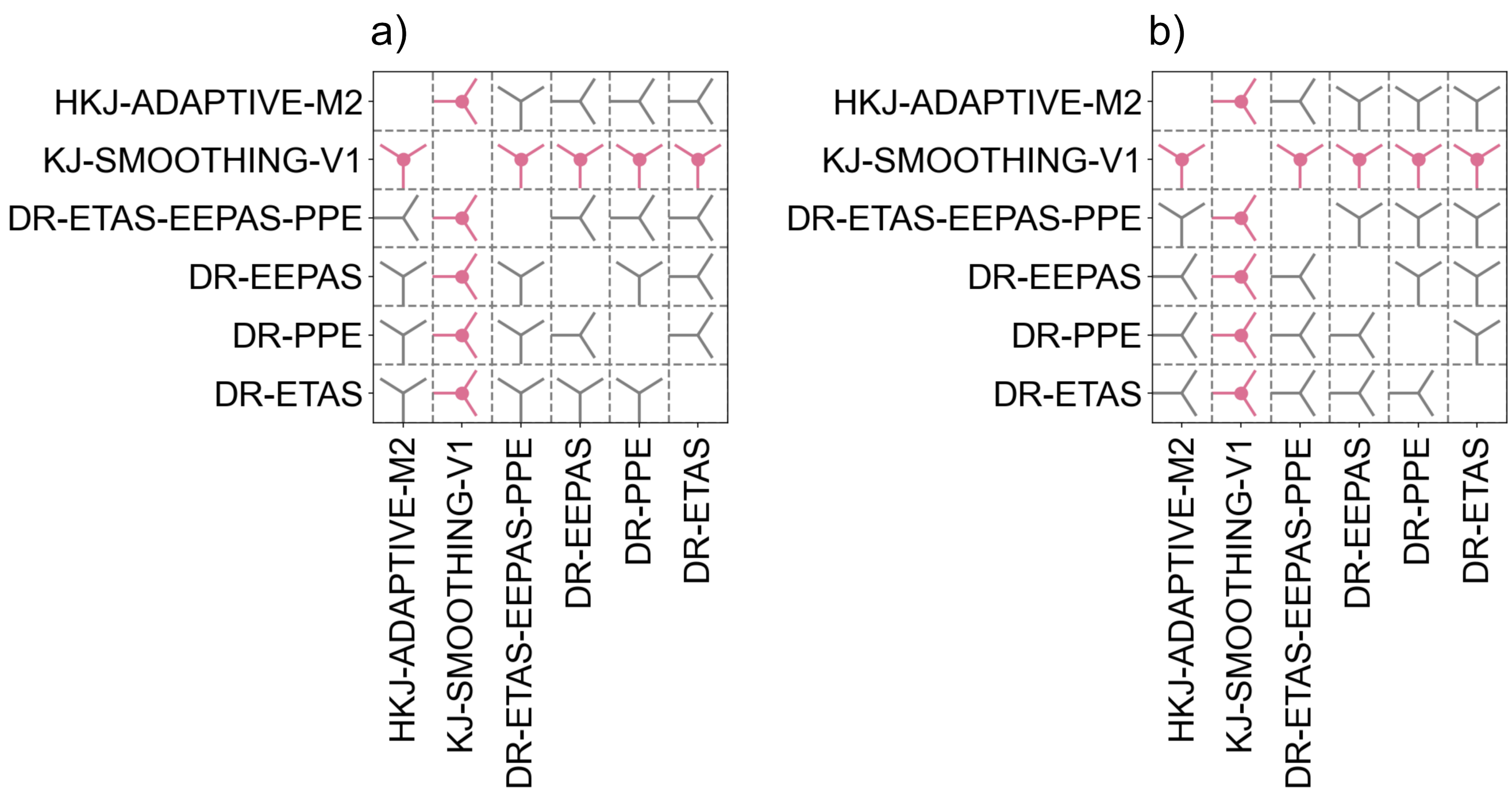

Supplementary Figure 10. Confusion matrices showing the significance of cumulative IGPAs obtained by competing $M_w \geq 4.95$ seismicity models (rows) over reference models (columns) between a) January 2013 and July 2016 [7 earthquakes; 7 cells] and b) October 2016 and August 2018 [6 earthquakes; 4 cells]. For each pairwise comparison, the aircraft symbol points to the most informative model. Pink aircraft symbols with midpoints indicate that the IGPA is statistically significant, while gray aircraft symbols indicate the opposite. Note that both matrices are symmetric with respect to the diagonal.

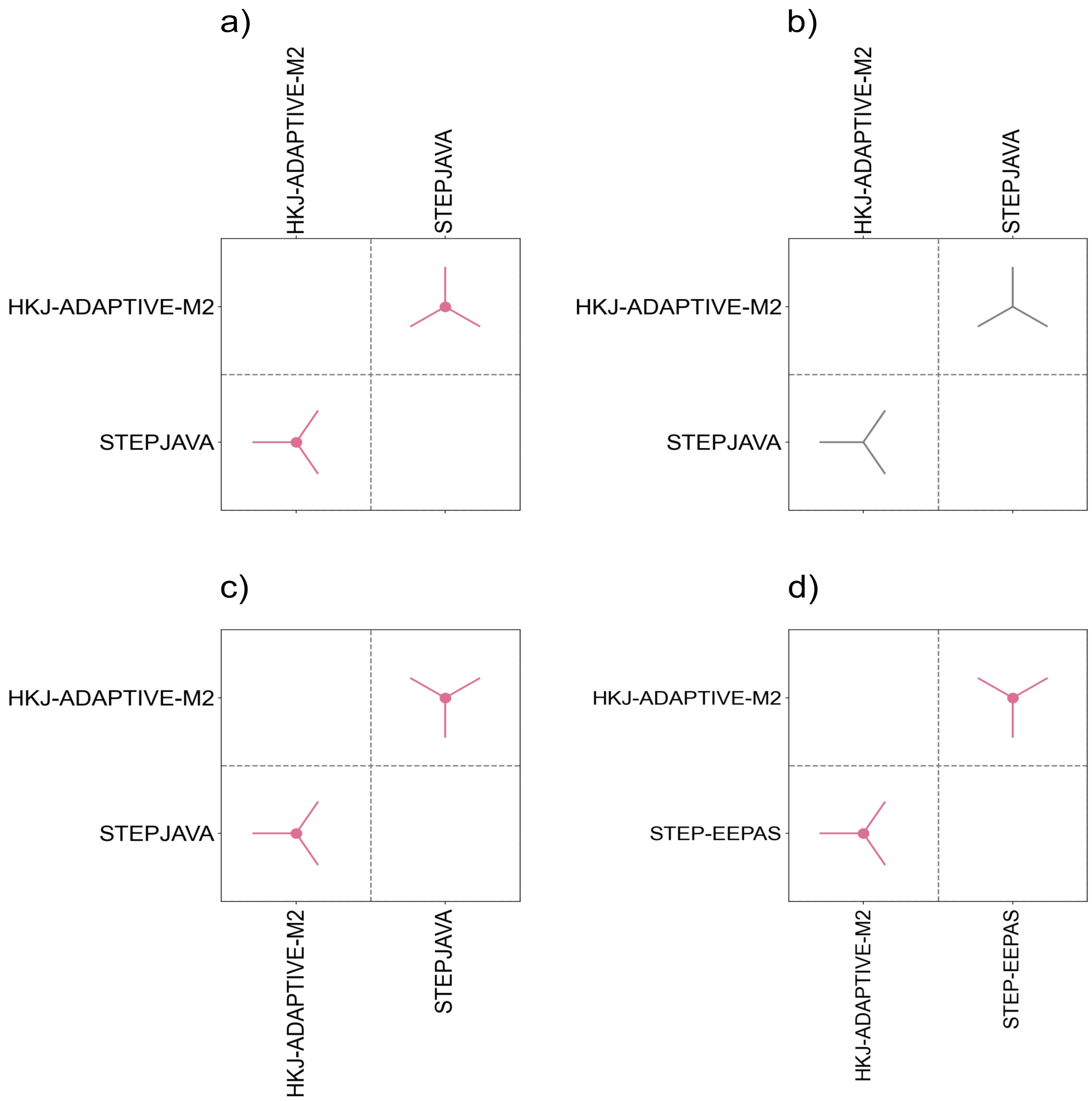

Supplementary Figure 11. Confusion matrices showing the significance of the IGPAs obtained by the STEPJAVA and STEP-EEPAS models over HKJ-ADAPTIVE-M2. We present these results separately, because STEPJAVA and STEP-EEPAS are defined within a smaller test region than the entire CSEP-California test region. In the case of the $M_w \geq 3.95$ STEPJAVA seismicity model, the significance of its cumulative IGPAs over HKJ-ADAPTIVE-M2 are estimated between a) September 2010 and July 2012 [72 earthquakes; 49 cells], b) July 2012 and October 2012 [24 earthquakes; 12 cells], and c) January 2013 and July 2016 [108 earthquakes; 65 cells]. In the main text, we only discuss IGPAs obtained by STEPJAVA over the time-invariant HKJ-ADAPTIVE-M2 model (first column of each panel). In the case of the $M_w \geq 4.95$ STEP-EEPAS seismicity model, such a statistical significance is estimated between d) October 2016 and August 2018 [5 earthquakes; 3 cells]. For each pairwise comparison, the aircraft symbol points to the most informative model. Pink aircraft symbols with midpoints indicate that the IGPA is statistically significant, while gray aircraft symbols indicate the opposite. Note that all matrices are symmetric with respect to the diagonal.

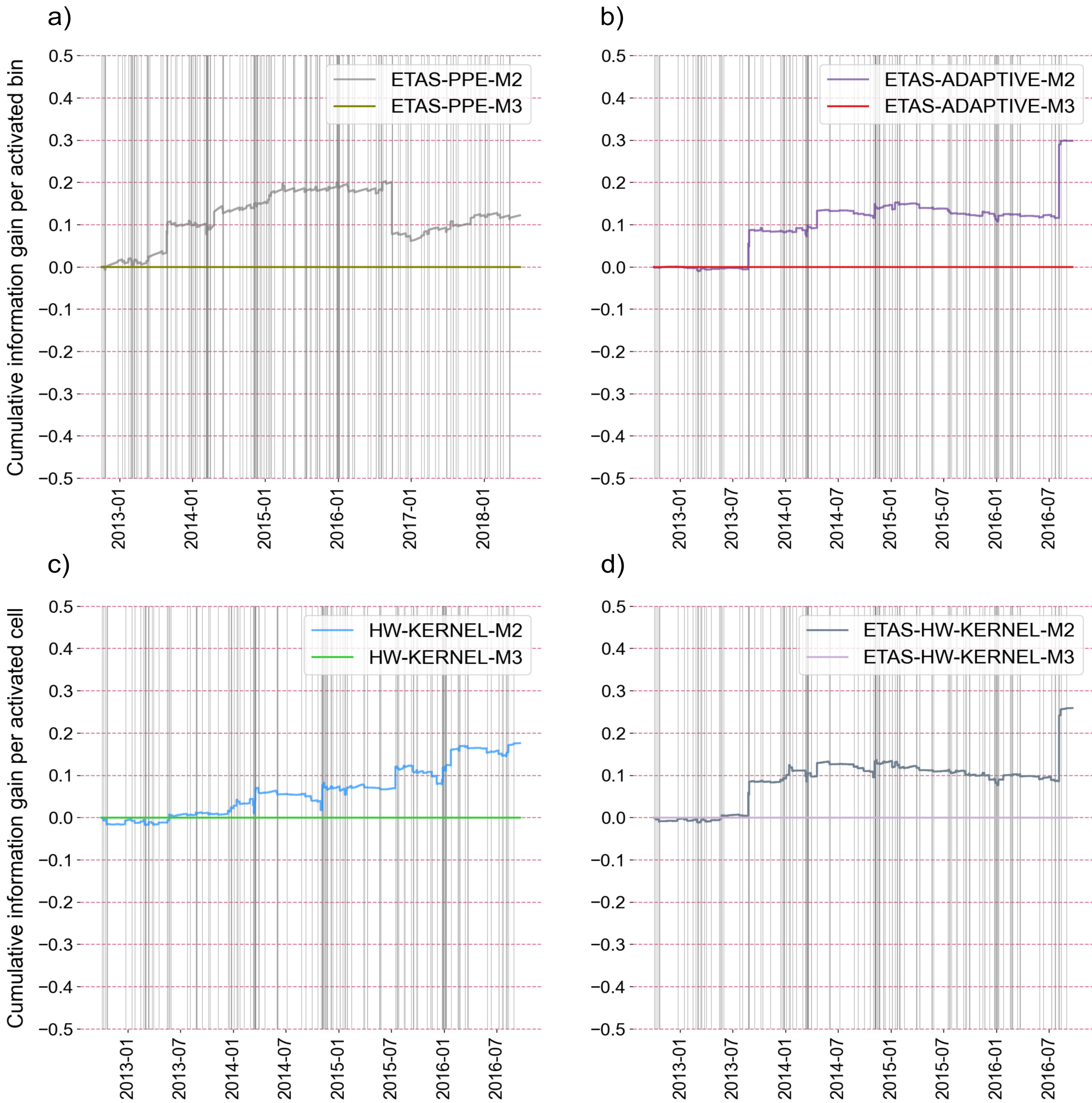

Supplementary Figure 12. Cumulative binary IGPAs obtained by time-varying $M_w \geq 3.95$ seismicity models calibrated on $M \geq 2.0$ seismicity over their analog models based on $M \geq 3.0$ earthquakes during a) October 2012 and July 2018 [188 earthquakes; 119 cells] and b–d) October 2012 and September 2016 [147 earthquakes; 92 cells]. Positive cumulative IGPAs indicate that the competing models are more informative than the reference models. However, these gains are statistically insignificant as the *p*-values of the underlying t-tests are smaller than 0.05. Gray vertical lines indicate the occurrence of $M_w \geq 4.95$ earthquakes during each evaluation period.

References

1. Zhuang, J. Next-day earthquake forecasts for the Japan region generated by the ETAS model. *Earth, planets space* **63**, 207–216, https://doi.org/10.5047/eps.2010.12.010 (2011).
2. Rhoades, D. Mixture models for improved earthquake forecasting with short-to-medium time horizons. *Bull. Seism. Society America* **103**, 2203–2215, https://doi.org/10.1785/0120120233 (2013).
3. Rhoades, D. & Evison, F. Long-range earthquake forecasting with every earthquake a precursor according to scale. *Pure applied geophysics* **161**, 47–72, https://doi.org/10.1007/s00024-003-2434-9 (2004).
4. Werner, M., Helmstetter, A., Jackson, D. & Kagan, Y. High-resolution long-term and short-term earthquake forecasts for California. *Bull. Seism. Society America* **101**, 1630–1648, https://doi.org/10.1785/0120090340 (2011).
5. Gerstenberger, M., Wiemer, S., Jones, L. & Reasenberg, P. Real-time forecasts of tomorrow's earthquakes in California. *Nature* **435**, 328–331, https://doi.org/10.1038/nature03622 (2005).
6. Frankel, A. D. *et al.* National seismic-hazard maps: documentation june 1996. Tech. Rep., US Geological Survey (1996). https://doi.org/10.3133/ofr96532.
7. Reasenberg, P. & Jones, L. Earthquake hazard after a mainshock in California. *Science* **243**, 1173–1176, https://doi.org/10.1126/science.243.4895.1173 (1989).
8. Helmstetter, A., Kagan, Y. & Jackson, D. High-resolution time-independent grid-based forecast for m>=5 earthquakes in California. *Seism. Research Letters* **78**, 78–86, https://doi.org/10.1785/gssrl.78.1.78 (2007).
9. Kagan, Y. & Jackson, D. Short-and long-term earthquake forecasts for California and Nevada. *Pure Applied Geophysics* **167**, 685–692, https://doi.org/10.1007/s00024-010-0073-5 (2010).
10. Helmstetter, A. & Werner, M. Adaptive smoothing of seismicity in time, space, and magnitude for time-dependent earthquake forecasts for California. *Bull. Seism. Society America* **104**, 809–822, https://doi.org/10.1785/0120130105 (2014).
11. Gordon, J., Fox, E. & Schoenberg, F. A nonparametric Hawkes model for forecasting California seismicity. *Bull. Seism. Society America* **111**, 2216–2234, https://doi.org/10.1785/0120200349 (2021).
12. Rhoades, D. & Gerstenberger, M. Mixture models for improved short-term earthquake forecasting. *Bull. Seism. Society America* **99**, 636–646, https://doi.org/10.1785/0120080063 (2009).
13. Serafini, F. *et al.* A benchmark database of ten years of prospective next-day earthquake forecasts in california from the collaboratory for the study of earthquake predictability. *Scientific Data* **12**, 1501, https://doi.org/10.1038/s41597-025-05766-3 (2025).
14. Zechar, J. *et al.* The Collaboratory for the Study of Earthquake Predictability perspective on computational earthquake science. *Concurr. Computation: Practice Experience* **22**, 1836–1847, https://doi.org/10.1002/cpe.1519 (2010).
