# Supplementary material for: Select earthquake forecasting models demonstrate consistency with prospective decadal observations in California
Source: Nat Commun. 2026 Aug 6;17:9444. doi: 10.1038/s41467-026-76243-7 (PMC13538450; doi:10.1038/s41467-026-76243-7)
Supplement: Supplementary file 2 [file 41467_2026_76243_MOESM2_ESM.pdf]

Description of the Additional Supplementary Files

supplementary Dataset 1:

Summary of key features, assumptions, and calibration datasets used to develop next-day M_w ≥ 3.95 earthquake forecasting models for California.

Supplementary Dataset 2:

Summary of key parameters used by M_w ≥ 3.95 earthquake forecasting models for California.
