# Supplementary material for: Select earthquake forecasting models demonstrate consistency with prospective decadal observations in California
Source: Nat Commun. 2026 Aug 6;17:9444. doi: 10.1038/s41467-026-76243-7 (PMC13538450; doi:10.1038/s41467-026-76243-7)
Supplement: Supplementary file 3 [file 41467_2026_76243_MOESM5_ESM.pdf]

nature portfolio

Peer Review File

Select earthquake forecasting models demonstrate consistency with decadal prospective observations in California

Corresponding Author: Dr José Bayona

This file contains all reviewer reports in order by version, followed by all author rebuttals in order by version.

Version 0:

Reviewer comments:

Reviewer #1

(Remarks to the Author)
This is an important study that fulfills a societal and scientific need. In the words of Dave Jackson, "Only prospective testing counts," about which they have admirably abided. The government agencies that produce (or are considering producing) operational earthquake forecasts should benefit greatly from this work. So, I believe the paper should be published in this journal.

With that said, the manuscript could be made much more readable. The abstract could be stronger and some of the writing is verbose. Some of the figures are extremely hard to decode, so the authors have resorted to the crutch of massive captions to explain them. Few readers will have the patience to read the captions in full. Better legending of the figures, and more intuitive presentation of the results, would ensure more readers, and also more persuaded readers. I have made suggestions to accomplish this in the annotated manuscript, and have annotated most of the figures.

(Remarks on code availability)

Reviewer #2

(Remarks to the Author)
This paper presents results for prospective CSEP testing of time-dependent (1-day) earthquake forecast models in California. The tests find that in general, most models do a good job of forecasting the numbers, locations, and magnitudes of the next day's earthquakes, both over the full test time period and during earthquake sequences. Most of the models also outperform a time-independent model, demonstrating the value of including time-dependence. This is a solid contribution to the testing of time-dependent earthquake forecasts, although the novelty is at times overstated (see similar CSEP testing for other regions by, e.g., Nanjo et al, 2012; Taroni et al., 2018; Rhoades et al, 2018).

(1) The longest-running models (ETAS, STEP, HKJ) start in 2007, implying that these are the RELM models. However, only the HKJ model is referenced to the corresponding RELM paper. ETAS is referenced to a Zhuang (2011) ETAS model for Japan, not to a California model, and also the cited paper came out years after this ETAS model apparently started running in prospective testing. Also, why aren't the rest of the RELM 24-hour models (e.g. Console et al., 2007) included in the testing? How were the other models selected for this study, and were any other 1-day models submitted to RELM/CSEP excluded from this study?

(2) It would be helpful if the models were referred to by names that clearly reflect their unique aspects, even if these aren't the "official" CSEP model names. For people not deep in the weeds of CSEP, the models' names are not always very useful in understanding what the models are (e.g. ETAS-DROneDayPPEMd2 is said to be a PPE model, but why does the name also contain ETAS, what do DR and Md2 mean, and OneDay isn't necessary because all the models are 1 day; JANUSOneDayEEPAS1F, what do JANUS and 1F mean; etc.) It would also be helpful to briefly explain each model when it's first mentioned (e.g. GSF-ISO14 and K3Md2 are almost completely unexplained, only that they are non-parametric). I know there's more detail in the companion data publication, but this paper needs to stand on its own.

(3) Table 1 would be more helpful with some brief explanation of the unique features of each model. It currently does not contain enough information to differentiate models (e.g. ETAS-DROneDayMd2 and ETAS-DROne-DayPPEMd2 have identical information). It would also be helpful if the begin and end times for each model were noted.

(4) The CSEP number test is known to be flawed because it assumes a Poisson distribution, which is inconsistent with the known clustering of earthquakes. Clustering leads to a wider distribution, with more bins with either very low or very high rate compared to the mean. The negative binomial distribution has been proposed to account for earthquake clustering, and produces a wider distribution, as expected. Both tests are applied here, with all the models (except KJSSOneDayCalifornia, please see my next point) consistent with the observed rate of earthquake when the negative binomial test is used. The paper rejects the negative binomial test, however, simply because all of the models (except KJSSOneDayCalifornia) passed this test. If you want to argue that the negative binomial tests are flawed, you need to include some actual evidence of this. Otherwise it seems more reasonable to conclude that all the models (except KJSSOneDayCalifornia) are consistent with the observed rate, given the realistic variability of earthquake rates due to clustering.

(5) It seems like something went very wrong overall with KJSSOneDayCalifornia, which produces extremely low forecast rates. It would improve the clarity of the results to discuss the problems of this model separately, instead of including this model in with the others throughout the paper (e.g. bringing down the performance of “older” models.)

(6) The percentage discrepancy score is also introduced to assess the forecast rates. The rationale for this score isn’t well-developed, nor is there any quantification of what scores indicate that the model is consistent with observations. The results are claimed to be “a remarkable level of predictive skill”. This claim needs to be backed up with either some quantitative assessment of what would be a “remarkable” percentage discrepancy score, or some evidence that prior forecast models would fail to meet the percentages found here.

(7) The percentage discrepancy score is also asymmetric between models that under- versus over-predict: models that underpredict can’t score larger than 100%, while models that overpredict have no upper bound on the score. For example, KJSSOneDayCalifornia performs terribly, forecasting ~50 events while 510 were observed. But, because this model underpredicts, its discrepancy score turns out better than some less-bad models that overpredict, e.g. the GSF models predicting ~80 events while 38 were observed.

(8) A few larger earthquake sequences are separated out to test how well the models forecast the rate of earthquakes in the sequence. Please explain how the aftershock regions for each sequence were selected.

(9) The question of why the models performed poorly for the first 2 days of the El Mayor-Cucapah sequence should be actually investigated, instead of just hypothesizing. Please check whether the catalogs provided for the models are indeed incomplete at the magnitude levels used by the models during the first few days. An incomplete catalog could affect both ETAS (not enough secondary triggering due to missing aftershocks) and STEP (underestimated productivity due to missing aftershocks.). The preferred explanation, that the productivity of the El Mayor earthquake was larger than the productivities of the models, needs to be supported, and should be checked by computing the productivity of that sequence using the same parameterization as the models. The Hardebeck et al. (2019) reference isn’t helpful here because they don’t compute a productivity for the El Mayor earthquake, nor do they compare their southern California Reasenberg-Jones productivity parameters with the productivity parameters of the CSEP models. I’ll note also that much of the El Mayor-Cucapah aftershock zone is outside the Southern California Seismic Network, so is likely to have more incompleteness problems in general than inside California. Because of this, I think this is not an ideal event to provide much insight into general forecast performance.

(10) The models were not all active for the full 11 years, and were active over different times within the study period. I wonder if it biases the CSEP test results at all to have different numbers of test events for each model. For example, it requires some number of observations to falsify a model with a given level of confidence, so models that have been operational longer may be more falsifiable than newer models. Additionally, different models will have covered different sequences, and each sequence has different behaviors that may be more or less difficult to model. For example, all of the models operating at the time of the El Mayor-Cucapah earthquake struggled with this sequence, which contains a large fraction of the observed $M \geq 3.95$ events. This suggests that simply being in operation at the time of this sequence may affect the overall test results for these models, again disproportionately affecting the older models.

(11) The test of the spatial distributions includes only seismically active days. Please explain why it doesn’t consider all days. The spatial tests should penalize models that forecast a high probability in a location with no earthquakes. The test as described appears to only include this penalty if an earthquake happened to occur somewhere else on that same day.

(12) One conclusion is that the models that are nonparametric or with adaptive smoothing perform best in the spatial tests. This seems like an important result that could guide future model development. It would be helpful then to give some specific recommendations for how to achieve the right level of smoothing.

(13) It’s reported that models that were fit to both long catalogs of $M \geq 3.95$ earthquakes and shorter catalogs of smaller earthquakes perform well. It’s hard to know what to make of this, and it seem potentially contradictory. Here again more specific recommendations for future model development would be helpful.

(14) At some places, the manuscript makes vague and possibly overstated claims, with little support.

a. “Decadal” in the title is a bit of an overstatement, as most models did not run for 10 years.
b. The abstract could use fewer empty phrases like “marking a significant milestone”, and more specifics about what was learned. Or, explain how this is a milestone, when there have been other similar CSEP comparisons of time-dependent models (e.g. Nanjo et al, 2012; Taroni et al., 2018; Rhoades et al, 2018).
c. The abstract implies that 27 models ran for a 11 year test period. This needs some caveats, because most of the models have been running for only about half that long.
d. L19-21: Note that there has been substantial work in probabilistic earthquake forecasting that significantly pre-dates CSEP. This includes both long-term PSHA (e.g. Frankel et al, 2000), and time-dependent earthquake forecasts (e.g. Ogata, 1988; Reasenberg & Jones, 1989). Please acknowledge some of this prior work, and/or rewrite this sentence so that it doesn’t overstate the role of CSEP in developing the field of probabilistic earthquake forecasting. CSEP has had a leading role in the push for prospective testing of forecasts, so it would be more appropriate to highlight that.
e. L27-28, L50: Please acknowledge here that most of the 27 models have not been operating for the full 11 years.
f. L87-90 & 373-374: Why is this a milestone achievement, there are already other CSEP studies that were automatically run with no parameter value updating. Is there some sort of bar for public use that is newly achieved?
g. L255: Is this remarkable? Similar types of models have performed well in other CSEP experiments, so it’s not very surprising, especially since the rest of the paragraph acknowledges that many of the models over or under-smooth.

Figures:

Figure 1. State what the white areas mean. Is this where the models are undefined?

Figure 3b. Note that for models that started after 2007, their “cumulative number” starts at the observed cumulative number at the time the model started. Therefore, the total cumulative number for each model can’t be directly compared.

Figure 4. The way this is plotted, it looks like the time of the largest event perfectly aligns with the time of a forecast. However, actually each event occurred part way through a forecast time period. I think that the forecast times are being rounded (or floored?) to integer days relative to the largest event. This plot would be more clear if the actual non-rounded relative times of the forecasts to the events were shown.

Figure 5. Like the main text, this figure capture could use some explanation of what observation is at the given quantile of what distribution.

Figure 6. I don’t see the gray polygons showing the aftershock zones. Is it just the region with colored grid points? Some panels also have cyan squares, are these the aftershock zones? Are the ζ values for ETAS-DROneDayMd2 and ETAS-DROneDayMd3 really 0?

Minor points & typos:

L33: STEP stands for Short-Term Earthquake Probability
L72: “variability of earthquakes ascribed” -> “variability of earthquake rate due to”
L82-87: Please remind the reader that each model may have a different number of events because each has unique begin and end dates.
L130: Briefly explain the spatial quantile tests, otherwise this is hard to follow.
L132, L211: Earlier (L27) it says there are 27 models, what happened to the other 6?
L145: Define ζ or don’t use it. Explain what these numbers quantify: quantile of what compared to what distribution.
L214: Strike “decadal”. None of these models ran for a full decade.
L272: Include a reference for the USGS finite fault model, e.g. Goldberg et al. (2022)
L307: “rate-and-state fraction” -> “rate-and-state friction”
L317: magnitude is missing
L346: “U.S. territories and commonwealths” -> “U.S. states and territories”
L347: The USGS produces forecasts only for international earthquakes with estimated fatalities >100, not for all Mw≥5.0 international earthquakes.
L384: This actually seems like a problem if the public is questioning the reliability of the forecasts. CSEP can assure people that the models being used have performed adequately in testing.
L437: redundant: “high proportion of low quantiles and a high proportion of low quantiles”
L457: I think the pink curve is the observed.
L460: The notation K_S keeps getting reused for different tests. I would be clearer if the p-value for each K-S test had a different notation, e.g. p_mag.
L474: “M_x=1” -> “M_1”, and “M_x=2” -> “M_2”.
L478: missing close parenthesis
Figure 4: “20% or fewer of them do not statistically match” -> “20% or fewer of them statistically match”

References:

Console, R., Murru, M., Catalli, F., and Falcone, G. (2007), Real time forecasts through an earthquake clustering model constrained by the rate-and-state constitutive law: Comparison with a purely stochastic ETAS model, Seismol. Res. Lett. 78, 49–56, doi:10.1785/gssrl.78.1.49.

Frankel, A. D., et al (2000). USGS national seismic hazard maps. Earthquake spectra, 16(1), 1-19.

Goldberg, D. E., Koch, P., Melgar, D., Riquelme, S., & Yeck, W. L. (2022). Beyond the teleseism: Introducing regional seismic and geodetic data into routine USGS finite-fault modeling. Seismological Society of America, 93(6), 3308-3323.

Nanjo, K. Z., Tsuruoka, H., Yokoi, S., Ogata, Y., Falcone, G., Hirata, N., ... & Zhuang, J. (2012). Predictability study on the aftershock sequence following the 2011 Tohoku-Oki, Japan, earthquake: First results. Geophysical Journal International, 191(2), 653-658.

Ogata, Y. (1988). Statistical models for earthquake occurrences and residual analysis for point processes. Journal of the American Statistical association, 83(401), 9-27.

Reasenberg, P. A., & Jones, L. M. (1989). Earthquake hazard after a mainshock in California. Science, 243(4895), 1173-1176.

Rhoades, D. A., Christophersen, A., Gerstenberger, M. C., Liukis, M., Silva, F., Marzocchi, W., ... & Jordan, T. H. (2018). Highlights from the first ten years of the New Zealand earthquake forecast testing center. Seismological Research Letters, 89(4), 1229-1237.

Taroni, M., Marzocchi, W., Schorlemmer, D., Werner, M. J., Wiemer, S., Zechar, J. D., ... & Euchner, F. (2018). Prospective CSEP evaluation of 1-day, 3-month, and 5-yr earthquake forecasts for Italy. Seismological Research Letters, 89(4), 1251-1261.

(Remarks on code availability)

Version 1:

Reviewer comments:

Reviewer #1

(Remarks to the Author)
I am satisfied that the authors have worked to strengthen and clarify the manuscript and figures in response to my review. I recommend publication without further revision.

(Remarks on code availability)

Reviewer #2

(Remarks to the Author)
The authors have done an excellent job of thoroughly responding to the review comments.

(Remarks on code availability)

Open Access This Peer Review File is licensed under a Creative Commons Attribution 4.0 International License, which permits use, sharing, adaptation, distribution and reproduction in any medium or format, as long as you give appropriate credit to the original author(s) and the source, provide a link to the Creative Commons license, and indicate if changes were made.
In cases where reviewers are anonymous, credit should be given to 'Anonymous Referee' and the source.
The images or other third party material in this Peer Review File are included in the article's Creative Commons license, unless indicated otherwise in a credit line to the material. If material is not included in the article's Creative Commons license and your intended use is not permitted by statutory regulation or exceeds the permitted use, you will need to obtain permission directly from the copyright holder.
To view a copy of this license, visit https://creativecommons.org/licenses/by/4.0/

We appreciate the constructive and helpful comments from both reviewers, which we have carefully addressed in the revised (annotated) manuscript and below. In the revised manuscript, sections removed from the original manuscript are highlighted in red, while the changes implemented are highlighted in blue. The reviewers' comments are reproduced verbatim below in bold, our replies in regular black font, and the specific changes requested are copied in italics.

REVIEWER 1

This is an important study that fulfils a societal and scientific need. In the words of Dave Jackson, "Only prospective testing counts," about which they have admirably abided. The government agencies that produce (or are considering producing) operational earthquake forecasts should benefit greatly from this work. So, I believe the paper should be published in this journal. With that said, the manuscript could be made much more readable. The abstract could be stronger and some of the writing is verbose. Some of the figures are extremely hard to decode, so the authors have resorted to the crutch of massive captions to explain them. Few readers will have the patience to read the captions in full. Better legending of the figures, and more intuitive presentation of the results, would ensure more readers, and also more persuaded readers. I have made suggestions to accomplish this in the annotated manuscript, and have annotated most of the figures.

We sincerely appreciate the constructive and helpful comments from Reviewer 1, particularly those related to presenting our results in ways that can more efficiently capture the attention of a readership as broad as that of Nature Communications. We have implemented most of their suggestions in the revised manuscript.

Please note that the comments below refer to in-text pdf annotations that we have copied here and addressed point by point.

What am I missing here? Why did the tests stop 7.5 years ago?

This is an unfortunate combination of the end of bespoke systems administration support for CSEP from SCEC (due to effective budget cuts) and the departure of a key CSEP software developer in 2016, which eventually resulted in a major change in operational CSEP strategy. This is explained also in the accompanying forecast database paper (Serafini et al., Nature Scientific Data, 2025). The old CSEP strategy involved testing centres with bespoke systems architecture (hardware), workflows and lots of maintenance and reprocessing as the models, model input/output and results grew very quickly over a decade. The systems administration demands and maintenance requirements grew alongside and eventually outgrew our resources. With the arrival of a new software developer in 2018, we changed our operations strategy entirely to community-developed, modular, open-source software (pyCSEP; Savran et al., 2022, SRL) and an application to run forecast experiments on any computer anywhere (floatCSEP; Iturrieta et al., 2026, JOSS). We have prioritized these developments over the analysis of previous experiments.

Personally, I dislike unfamiliar acronyms. You are saving photons at the expense of making it harder to read.

Following this suggestion and Nature Communications' author guidelines, we have removed any acronyms from the abstract:

" Earthquake forecasting systems are now operationalized by agencies in several countries, providing situational awareness to at-risk communities. To ensure that forecasts follow the best science available, the underlying models require rigorous testing against prospective data and benchmarking. Between August 2007 and August 2018, 27 forecasting models, including the types used for operational earthquake forecasting, were prospectively operated as part of a multi-institutional project by the Collaboratory for the Study of Earthquake Predictability, generating 51,625 next-day Mw ≥ 3.95 seismicity forecasts for California. Here, we comprehensively evaluate their predictive skills against 597 Mw ≥ 3.95 earthquakes using community-vetted statistical methods. We show that approximately 95%, two-thirds, and 95% of the models produced forecasts consistent with the number, locations, and magnitudes of observed earthquakes, respectively. Individually, select versions of well-established, operational-type models demonstrated sustained consistency with prospective observations. We provide detailed recommendations, software tools, and benchmarking data to support the development of operational earthquake forecasting systems worldwide. "

Prospectively operated is more important.

We agree and have included this description in the revised abstract of this work.

Awkwardly written

We have modified this paragraph to avoid confusion (see above).

How many M ≥ 6 shocks?

Our prospective evaluation database contains 4 $M_w \geq 6.0$ earthquakes: the 2010 M_w 6.5 Ferndale, 2012 M_w 7.2 El Mayor-Cucapah, 2014 M_w 6.8 Mendocino and 2014 M_w 6.0 South Napa earthquakes. However, given the relatively short abstract format of Nature Communications, we provide these details in the Introduction and the (updated) "How good were the models at forecasting the daily number of events observed during specific earthquake scenarios?" sections of the revised manuscript:

" During the 11 years in which the models were operating prospectively, the Comprehensive Earthquake Catalog (ComCat) of the Advanced National Seismic System (ANSS)[19] reported 597 $M_w \geq 3.95$ earthquakes with hypocentral depths ≤ 30 km in the CSEP-California test region[21], including the 2010 $M_w \geq 7.2$ El Mayor-Cucapah earthquake sequence[22], the 2012 $M_w \geq 3.95$ Brawley seismic swarm[23], and the 2014 $M_w \geq 6.0$ South Napa earthquake[24] " (lines 54-57).

"We [...] analyze (in)consistencies between observed and predicted daily counts during a) the M_w 6.5 Ferndale earthquake sequence, whose largest event struck off the coast of Northern California on January 10, 2010[32]; b) the M_w 7.2 El Mayor-Cucapah earthquake on April 4, 2010, which is the largest

earthquake to hit California since the 1992 M_w 7.3 Landers earthquake[22]; c) the Brawley seismic swarm of August 26, 2012, which has been claimed to be induced by fluid pumping into a nearby geothermal field[23]; d) the M_w 6.8 earthquake that occurred in the Mendocino area of Northern California on March 10, 2014; e) the M_w 6.0 South Napa earthquake of August 24, 2014, which, contrary to expectations based on the Båth's law[33], did not trigger any $M_w \geq 3.95$ earthquakes[24,34]; and f) a randomly-selected period of relative seismic calm in California, spanning from January 1 to 20, 2018. " (lines 115-122).

Evaluate everywhere in CA, or only in these ruptures zones?

We have conducted prospective evaluations of next-day seismicity models defined across the entire CSEP-California testing region (Schorlemmer and Gerstenberger, 2007), which includes all of California and a boundary zone of approximately one degree around the state (see the grey solid polygon in Fig. 2).

This is the kind of debilitating detail that belongs in the figures or tables. Stick to the principal results in the text.

We agree. Our goal is to make it easier for Nature Communications' multidisciplinary readers to understand the underlying science and implications of our findings. Therefore, we have removed most of these details from the main text, focusing on the most useful results and updating Figure 3 so that readers can examine percentage discrepancies between observations and predictions in greater detail. For example:

" For practitioners working with (symmetric) percentage differences between forecasts and observations, we further show that 9 (almost half) of the 21 time-varying $M_w \geq 3.95$ seismicity models provided earthquake counts that differed by 20% or less from the total number of observed earthquakes. Among these models are versions of STEP and ETAS, such as STEPJAVA, ETAS-SMOOTHING-V0, and STEP, which over periods of approximately 8, 11, and 6 years forecasted nearly 254, 678, and 398 $M_w \geq 3.95$ earthquakes, respectively, differing by 3%, 17% and 17% from the 246, 579, and 332 events observed during such periods, respectively. " (lines 92-99).

That's news to me: Amazing

The apparent unproductivity of this event was indeed so surprising that it prompted USGS scientists, such as Parsons et al. (2014), Llenos and Michael (2017) and Hardeberck et al. (2019), to investigate it further. Llenos and Michael (2017), in particular, discovered that the low aftershock productivity observed in the South Napa region is not entirely anomalous and, in fact, earthquake sequences in Northern California appear to generate, on average, fewer aftershocks than Southern California earthquakes.

While true, does this really apply to M ≥ 3.95 quakes? Don't over-reach.

We acknowledge that, for California and other seismically resilient regions, M4.0+ earthquakes are more likely to inflict damage and loss, so we have added this nuance in the revised version of the manuscript:

> *" Properly forecasting where earthquakes are most likely to occur can help vulnerable communities implement more targeted mitigation plans, as earthquakes, especially after major events, cluster not only temporally but also spatially. "* (lines 148-149).

Surely, M ≥ 3.95 is below the magnitude of completeness in 1932.

It has been estimated that, for California and Nevada, the Comprehensive Earthquake Catalog of the Advanced National Seismic System is complete for magnitudes M ≥ 4.0 since 1932 (Feltzer and Cao, 2007), which is why it has been used as an authoritative catalog for developing and testing CSEP M ≥ 4.0 earthquake forecasting models for California (Schorlemmer and Gerstenberger, 2007). So, while we cannot guarantee that modelers took into account potential catalog incompleteness issues, we can assure that the prospective evaluation catalog is complete for magnitudes M ≥ 3.95 since 2007.

That's remarkable.

We have highlighted this finding in the Discussion section of the revised manuscript:

> *" We also highlight the remarkable performance of HKJ-ADAPTIVE-M2 during the 2012 Brawley seismic swarm, as this model, like the time-varying models, were not explicitly designed to account for variations in background rates that arise from features not contained in seismicity catalogs, such as aseismic processes[47]. This swarm lasted a total of one month, although its peak activity occurred on August 22, producing 9 M_w ≥ 3.95 shocks[23]. In response, over 80% of the models adequately forecasted the (lack of) observed earthquakes during the 15 days following these events. In fact, during the period from July to October 2012, HKJ-ADAPTIVE-M2 was found to be statistically as informative as most of the next-day models (Figs. 8d, and S9d), providing support for the use of these models, as a first approximation, to describe the temporal evolution of swarm-type seismicity. "* (lines 316-326).

Fig. 5 does not make this evident.

As suggested by the reviewer, we have updated Fig. 5 to facilitate the interpretation of observed and expected spatial quantiles distributions (see the new labels). The position and shape of the observed quantile distributions (pink curves) relative to the predicted distributions (gray diagonals) provide useful information about the level of spatial precision and accuracy offered by the models. Specifically, if the pink curves lie above the gray diagonals, this indicates that the models provided inaccurate or overly localized spatial forecasts compared to observations, whereas, if the pink curves lie above the diagonals, this indicates that the models generated overly smoothed (imprecise) spatial forecasts (see the figure caption in the revised manuscript).

We have also included further information on how to interpret the pink areas between the curves and the diagonals.

“ [...] we perform CSEP binary spatial tests to assess, for each model and for each of the days between August 2007 and August 2018 on which $M_w \geq 3.95$ earthquakes were recorded in California, the log-likelihoods (referred to here as jbill values) of observing either no seismicity or at least one target earthquake in each of the forecasts' spatial grid cells (see the Methods section). This test is summarized by the observed spatial quantile ζ, which is the fraction of simulated catalogs of target activated cells with spatial log-likelihoods smaller than the jbill observed on a seismically active day. If $\zeta > 0.05$, then the spatial distributions of observed and predicted earthquakes can be considered statistically indistinguishable.

By sorting for each model all the observed spatial quantiles in ascending order (pink curves in Fig. 5) and comparing these distributions with spatial quantiles drawn from uniform distributions (gray dashed diagonals), we show that 15 (~71%) of the 21 time-varying $M_w \geq 3.95$ seismicity models generated daily earthquake forecasts statistically consistent with the spatial distribution of observed epicenters (see the pink areas [A] between the curves and the diagonals; the smaller the area, the greater the agreement between forecasts and observations). ” (lines 150-163)

“ The shape and position of the pink curves relative to the diagonals in Fig.5 provide additional information on the level of spatial precision that the models can offer. In particular, if the pink curves lie below the diagonals, this indicates that models generated spatially overly smoothed forecasts compared to observations, whereas, if the pink curves lie above the diagonals, this denotes that models provided spatial forecasts that are too localized or inaccurate [...] ” (lines 169-173)

That’s important.

We agree that this is a very important finding. In the Discussion section and in Table S1 of the revised manuscript, we have provided details about the assumptions made by some of the most consistent models with the spatial distribution of observed earthquakes:

“ Moving into the spatial domain of the models, we find that 15 of the 21 evaluated models generated earthquake forecasts consistent with the spatial distribution of observed epicenters (see Fig. 5). This is a remarkable achievement considering that none of the different spatial parameters/ methods was updated throughout the evaluation period. Broadly speaking, models that incorporated nonparametric components and "intermediate" smoothing procedures, such as adaptive smoothing, produced the most consistent spatial forecasts. E.g., the HW-KERNEL-M models, which employ*

a power-law kernel with variable bandwidth, along with the ETAS-ADAPTIVE-M models, which assume a power-law kernel for M < 5.5 triggering earthquakes and an anisotropic one for M ≥ 5.5 events, were among the most consistent with observations. In contrast, models such as ETAS-SMOOTHING-V0 and STEP, which predominantly use fixed isotropic smoothing functions, exhibited the poorest spatial performances, producing oversmoothed and overlocalized seismicity forecasts, respectively. The spatial limitations of ETAS-SMOOTHING-V0, nonetheless, appear to have been addressed by its successor model, ETAS-SMOOTHING-V1, which uses a comparatively smaller kernel bandwidth (see Fig. 1), resulting in improved spatial forecasting (see Figs. 8e and S9e). "* (lines 327-343)

This is an important finding, because it is contrary to what is used in the NSHMP and UCERF3, which have strongly characteristic frequency-magnitude curves.

We agree that this is indeed a very interesting observation; however, we refrain from discussing its implications for seismic hazard assessments or future UCERF3 developments, as no M ≥ 6.0 earthquakes occurred in California during the 6 years in which STEPJAVA was operational. However, in the revised manuscript , we comment on the similarities between the frequency-magnitude distribution predicted by STEPJAVA and a characteristic-type distribution

---

" The results for STEPJAVA are most likely due to its predicted distribution deviating from a typical Gutenberg-Richter distribution towards higher values within the 6.0 - 7.0 magnitude range (similar to a characteristic-type distribution) and the fact that no M_w ≥ earthquakes occurred in the STEPJAVA region during the time the model was operational. " (lines 359-362).

I think you should name GEAR1, as you name all the others. GEAR1 has now been tested against 1500 M ≥ 6, 150 M ≥ 7, and 8 ≥ 8 prospective shocks. Those magnitudes are meaningful with respect to your comments about societal relevance.

We appreciate the suggestion and have explicitly mentioned GEAR1 as an example of global ensemble models that outperform their individual model components in prospective evaluations:

" [..], on a global scale, contrasting prospective test results have also been reported, with ensemble models such as the Global Earthquake Activity Rate (GEAR1) model[56] outperforming its two individual model components based separately on smoothed seismicity and interseismic strain rates[57,58]. " (lines 392-395)

This section could be more concise

For the revised manuscript, we have strived to convey the key messages as concisely and clearly as possible.

Nice.

Our goal is that the results, data, and software resources provided in this work will be of practical use for the establishment, development, and improvement of operational earthquake forecasting systems worldwide.

Very hard to read these postage-stamp maps. If you are hoping that readers will zoom in on the maps, the figure should be provided at much higher resolution.

We have provided forecast maps with a much higher resolution.

It would be helpful to have a second scale for the number of M ≥ 3.95 quakes per day for all of California, since 10^-6 per day per cell (a return period of 2740 yr!) is so unintuitive.

The number of $M_w \geq 3.95$ earthquakes per day in California, λ, is the sum of the expected number of earthquakes per cell across all cells. Therefore, the unique expected earthquake rate per day per model does not require a second scale. However, we have included these expected values in the revised Fig. 1.

This figure would be much more readable if you used disks rather than squares for quakes. The labels also interfere with the seismicity, the jagged buffer zone is distracting (the least important element of the figure is the most prominent), and the location map is not needed. If there is not a strong reason for the variable magnitude scale differences, keep it at 1 M unit.

All of the reviewer's suggestions have been implemented in the revised figure 2: earthquake epicenters are now represented as circles, the labels no longer interfere with the seismicity, the location map has been removed, and the new figure legend now reports magnitude scale differences in 1M units.

Each model has a different start date. Because models are buried atop one another, this makes it hard for the reader to compare model performance by eye. Could you indicate the start date with the appropriately colored dot?

In the revised figure 3b, we have added dashed pink vertical lines to indicate the different dates on which various model subsets became operational. Above each line, we have also included (in square brackets) the numbers of new models that were installed.

How about ordering these from best performers to worst? (Be kind to your readers)

We welcome the suggestion and have implemented it in the new version of figure 3b.

This figure is unnecessarily hard to decode. The circles are the observations, while the color of the circles gives the percentage of models consistent with those observations. This feels to me like a strange mixture of model and data. Then you have expected decays for a subset of models as dot-dash lines, with the grey areas the model prediction ranges. You are counting on a lot of patience of your readers and a close reading of the long caption.

We have improved the figure caption to make it easier to interpret. In short, if the daily number of observed $M_w \geq 3.95$ earthquakes (circles) falls within the 95% predictive intervals of the models

(shown in gray), then observations and forecasts are statistically consistent. The color of the circles indicate the percentage of models (Z) that statistically capture the daily observations.

You are depending too much on readers meticulously slogging through your massive caption, rather than helpfully legending your figure. Why not use the blank space (STEP) to provide interpretation guidelines. Above the diagonal, put, "spatially too localized", below the diagonal, put "spatially too smoothed". Along the diagonal, "perfect fit".

We thank the reviewer for the useful suggestion. In the updated Fig. 5, we have included labels describing how to interpret the (in)consistencies between models and observations. Furthermore, in the revised manuscript, we have also included a brief description of how to interpret the annotated areas (A):

> *" [...] the smaller the area, the greater the agreement between forecasts and observations [...] "* (lines 162-163)

It's hard for the reader to gain insights from these tiny maps. One ends up just looking at the statistics. I think the idea is that turquoise quakes should correspond to yellow (high) forecast aftershock rates. If so, I think all these maps should be zoomed in so one can actually see if there is a correlation of that kind. If I have it wrong, then maybe a table would be better than these mini-maps.

We have created new earthquake and aftershock forecast maps confined within a (smaller) aftershock zone equivalent to a circular region with radius equal to three times the average length of each main event based on the scaling relationships of Wells and Coppersmith (1994). The new maps are also provided at a higher resolution so readers can analyze them in greater detail. The reviewer is correct in interpreting that the epicenters of observed earthquakes should fall within/close to the bright-colored forecast cells to indicate consistency. However, to account for forecast uncertainties, the expected rates in cells surrounding the observations should also have similar values (or colors). Otherwise, the spatial distribution of expected earthquakes may be too localized compared to observations. We have provided further information in the figure caption to facilitate understanding of these maps:

> *" Annotated 'jbill' values are binary log-likelihoods of observing the data under the models. Greater jbill values indicate greater consistency with observations. If the annotated spatial quantile ζ is greater than 0.05, the spatial forecasts are statistically consistent with the spatial distribution of observed seismicity. "*

So all models fail Napa except HKJ. But should Napa be therefore omitted?

We did not assess (in)consistencies between the spatial distribution of the 2014 M_w 6.0 South Napa earthquake sequence and spatial forecasts generated by the models, because this event did not trigger any M ≥ 3.95 seismicity (see Fig. 4e). However, in terms of expected rates, all models included 0 aftershocks within the 95% range of the Poisson forecasts. We have deleted any information regarding this earthquake from the caption of figure 6 to avoid any confusion.

REVIEWER 2

This paper presents results for prospective CSEP testing of time-dependent (1-day) earthquake forecast models in California. The tests find that in general, most models do a good job of forecasting the numbers, locations, and magnitudes of the next day's earthquakes, both over the full test time period and during earthquake sequences. Most of the models also outperform a time-independent model, demonstrating the value of including time-dependence. This is a solid contribution to the testing of time-dependent earthquake forecasts, although the novelty is at times overstated (see similar CSEP testing for other regions by, e.g., Nanjo et al, 2012; Taroni et al., 2018; Rhoades et al, 2018).

We greatly appreciate the reviewers' comprehensive and constructive comments, particularly those concerning the novelty of this work. To our knowledge, there is no other curated and fully prospective database of next-day M ≥ 3.95 earthquake forecasts in the world with such high spatiotemporal resolution and such a long duration. This provides the unique opportunity to rigorously and without bias assess (in)consistencies and predictive skills of seismicity models with yearslong out-of-sample observations. Previous CSEP forecasting experiments have been conducted over shorter time windows, comprising either less prospective data or fewer seismicity models / hypotheses about seismogenesis. E.g., the CSEP forecasting experiment in Japan (Nanjo et al., 2012) was based on approximately 700 prospective M5.0+ earthquakes recorded between March 8 and 28, 2011, but only involved 5 different seismicity models. In contrast, the CSEP-Italy forecast experiment took place over a 5-year period (August 2009–August 2014), but only involved 4 seismicity models and 97 M ≥ 3.95 prospective earthquakes.

The fact that 27 different models, including versions of the well-established ETAS, STEP and PPE models, non-parametric models, and ensemble models, were prospectively operated over extended periods on CSEP servers without any human intervention and without updates to their parameters is what, in our opinion, makes this forecast experiment unprecedented. This shift was only made possible through the long-term collaboration of nine CSEP research groups based in Italy, the United States, New Zealand, Japan, and the United Kingdom. Even more astonishing is the fact that select versions of ETAS and STEP, which are the main types of clustering models used for operational earthquake forecasting (OEF) in some countries, demonstrated sustained consistency with prospective observations. We consider that this remarkable achievement in the wider field of statical seismology, providing compelling evidence for the use of these types of models in public OEF.

(1) The longest-running models (ETAS, STEP, HKJ) start in 2007, implying that these are the RELM models. However, only the HKJ model is referenced to the corresponding RELM paper. ETAS is referenced to a Zhuang (2011) ETAS model for Japan, not to a California model, and also the cited paper came out years after this ETAS model apparently started running in prospective testing. Also, why aren't the rest of the RELM 24-hour models (e.g. Console et al., 2007) included in the testing? How were the other models selected for this study, and were any other 1-day models submitted to RELM/CSEP excluded from this study?

Some of the evaluated models, such as STEP and (the now called) PPE-BACKGROUND-M2 model, are indeed part of the RELM experiment, organized by CSEP (Field, 2007). However, we did not include the ETAS-type model by Console et al. (2007), because its code was not formally installed on CSEP servers and, consequently, its forecasts were not implemented. Similarly, we did not evaluate the 24-hour smoothed seismicity and "hidden Markov" models of Ebel et al. (2007), also described in Field (2007), because their seismicity forecasts were not generated in

practice. Thus, we would like to clarify that we did not select or exclude any models from this experiment; we simply evaluated the entire next-day earthquake forecast database available, which contains 51,625 forecasts.

Regarding the (now called) ETAS-SMOOTHING-V0 model, we provide the reference of its sibling version, the ETAS model for Japan (Zhuang, 2011), since both models are based on similar assumptions and because there is no formal scientific article describing this model. We contacted the creator of this model (and of ETAS-SMOOTHING-V1), Prof. Jiangcang Zhuang (Institute of Statistical Mathematics), to raise this concern, and he suggested that we provide the reference for the Japanese ETAS model.

(2) It would be helpful if the models were referred to by names that clearly reflect their unique aspects, even if these aren't the "official" CSEP model names. For people not deep in the weeds of CSEP, the models' names are not always very useful in understanding what the models are (e.g. ETAS-DROneDayPPEMd2 is said to be a PPE model, but why does the name also contain ETAS, what do DR and Md2 mean, and OneDay isn't necessary because all the models are 1 day; JANUSOneDayEEPAS1F, what do JANUS and 1F mean; etc.) It would also be helpful to briefly explain each model when it's first mentioned (e.g. GSF-ISO14 and K3Md2 are almost completely unexplained, only that they are non-parametric). I know there's more detail in the companion data publication, but this paper needs to stand on its own.

We thank the reviewer for their useful suggestion. We agree that the official names of the models do not provide much information about their distinctive features/assumptions, so we have assigned them new names that better summarize their unique characteristics (see the revised manuscript). For example, the former ETAS-DROneDayPPEMd* models, are now referred to as the PPE-BACKGROUND-M* models, while JANUSOneDayEEPAS1F is now the DR-EEPAS model. In the supplementary material for this article, we have also included a new Table S1 that provides additional information about the datasets, assumptions, and parameters used by all these models.

(3) Table 1 would be more helpful with some brief explanation of the unique features of each model. It currently does not contain enough information to differentiate models (e.g. ETAS-DROneDayMd2 and ETAS-DROne-DayPPEMd2 have identical information). It would also be helpful if the begin and end times for each model were noted.

The new Table S1 provides detailed information on the unique features of each model, complementing the (now) Table S2, which in the original manuscript was referred to as Table 1.

(4) The CSEP number test is known to be flawed because it assumes a Poisson distribution, which is inconsistent with the known clustering of earthquakes. Clustering leads to a wider distribution, with more bins with either very low or very high rate compared to the mean. The negative binomial distribution has been proposed to account for earthquake clustering, and produces a wider distribution, as expected. Both tests are applied here, with all the models (except KJSSOneDayCalifornia, please see my next point) consistent with the observed rate of earthquake when the negative binomial test is used. The paper rejects the negative binomial test, however, simply because all of the models (except KJSSOneDayCalifornia) passed this test. If you want to argue that the negative binomial tests are flawed, you need to include some actual evidence of this. Otherwise it seems more reasonable to conclude

that all the models (except KJSSOneDayCalifornia) are consistent with the observed rate, given the realistic variability of earthquake rates due to clustering.

We also thank the reviewer for raising this point. We in no way intended to suggest that the negative binomial distribution (NBD) number test is flawed because of the large variances it provides; we simply felt that its predictive intervals may be too wide for certain forecast users, particularly those working with percentage discrepancies between observations and predictions. In fact, we use this test because it allows us to estimate, based on historical data, the variances that the models do not provide. Therefore, we agree in concluding that, based on the results of the NBD number test, 20 of the 21 evaluated models were statistically consistent with the cumulative number of prospective observations (see the abstract above and the following descriptions in the revised manuscript):

“ [...] we show that, with the exception of KJ-SMOOTHING-V0, all models statistically matched the observations (see how the circles fall within the models' 95% predictive intervals, shown as gray dashed bars, in Fig. 3c). ” (lines 84-86)

“ Prospective evaluations reveal that the 21 time-varying $M_w \geq 3.95$ seismicity models, as a whole, were most effective at forecasting the number and magnitude distributions of observed earthquakes, followed by their epicentral locations. [...]. Regarding the number components, we show that, based on the CSEP Negative Binomial Distribution number test[30], 20 models statistically captured the cumulative number of observed earthquakes (see Fig. 3c). ” (lines 261-268)

“ Therefore, we used multiple statistical metrics that minimize the resulting bias, finding that i) 95% of the models statistically captured the cumulative number of observed prospective earthquakes [...] ” (lines 487-488)

---

(5) It seems like something went very wrong overall with KJSSOneDayCalifornia, which produces extremely low forecast rates. It would improve the clarity of the results to discuss the problems of this model separately, instead of including this model in with the others throughout the paper (e.g. bringing down the performance of “older” models.)

The (now called) KJ-SMOOTHING-V* models had indeed difficulty adequately forecasting the observations. We believe that technical implications in the codes generating next-day forecasts could explain these large inconsistencies (e.g., wrong calibration, bugs, (un)commented lines; see below), and therefore we advocate for future evaluations of revised versions of forecasts generated by these models. Off the record, we also believe that with a bit more smoothing the currently spatially overlocalized forecasts provided by these models would be more consistent with observations (see Fig. 6).

“ In the particular case of KJ-SMOOTHING-V0 (and its sibling model KJ-SMOOTHING-V1), we also advocate for technical reviews of the codes that generate their seismicity forecasts, as both models presented notable difficulties in statistically capturing the number of observed earthquakes. While KJ-SMOOTHING-V0 significantly underestimated the 510 $M_w \geq 3.95$ earthquakes observed over a period of almost 10 years (Fig. 3), KJ-SMOOTHING-V1 significantly overestimated the number of observations, providing a long-term rate of approximately 1.6E-3 $M_w \geq 4.95$ earthquakes per day per km^2 (estimated from forecast files) that differed substantially from the rate of approximately 3.5E-7 $M_w \geq 4.95$ earthquakes per day per km^2 reported for the model[45] (see Fig. S3). Revised versions of these models would undoubtedly honor the unparalleled contributions, legacy, and impact of Prof. Yan Kagan and Prof. Dave Jackson (KJ in KJ-SMOOTHING-V) within and beyond the field of seismology[46]. ”* (lines 301-309)

(6) The percentage discrepancy score is also introduced to assess the forecast rates. The rationale for this score isn’t well-developed, nor is there any quantification of what scores indicate that the model is consistent with observations. The results are claimed to be “a remarkable level of predictive skill”. This claim needs to be backed up with either some quantitative assessment of what would be a “remarkable” percentage discrepancy score, or some evidence that prior forecast models would fail to meet the percentages found here.

In the revised manuscript, we make explicit that the CSEP (Poisson) and Negative Binomial Distribution number tests are the only metrics we use to assess number (in)consistencies between models and observations:

“ [...] we assess number (in)consistencies between forecasts and observations during the different periods in which the underlying models were operational. Prospective results of the CSEP number test[28] show that only ETAS-PPE-M2.95 and STEPJAVA forecasts were statistically consistent with the observed data (see the proximity between the circles [observations] and the black solid bars [models' predictive intervals] in Fig. 3c). This (in)consistency test, however, relies heavily on the assumption that the number of earthquakes can be modeled using a Poisson distribution, which depends on a single parameter and is not particularly skewed, resulting in predictive intervals that do not fully capture the spatiotemporal variability of earthquake rates due to their strong clustering nature[29]. ” (lines 74-81)

Acknowledging this limitation, we also perform the CSEP Negative Binomial Distribution number test, whose underlying probability distribution exhibits a larger variance than the Poisson distribution and can therefore be used to better account for earthquake-rate variability[30]. In this case, we show that, with the exception of KJ-SMOOTHING-V0, all models statistically matched the observations (see how the circles fall within the models' 95% predictive intervals, shown as gray dashed bars, in Fig. 3c). ” (lines 82-86)

(7) The percentage discrepancy score is also asymmetric between models that under- versus over-predict: models that underpredict can't score larger than 100%, while models that overpredict have no upper bound on the score. For example, KJSSOneDayCalifornia performs terribly, forecasting ~50 events while 510 were observed. But, because this model underpredicts, its discrepancy score turns out better than some less-bad models that overpredict, e.g. the GSF models predicting ~80 events while 38 were observed.

We reiterate that these percentage discrepancies are not intended to function as scores or metrics in this model evaluation. However, we agree that these percentage discrepancies, as they stand, are asymmetric between models that under- and overpredict earthquakes. Thus, we have calculated new symmetric percentage discrepancies between forecasts and observations, as:

$\Delta = |N_{fore} - N_{obs}| / \max(N_{fore}, N_{obs}) * 100$

By estimating these symmetric percentage deviations, we can now report that 9 of the 21 $M_w \geq 3.95$ seismicity models, provided earthquake counts that differed by less than 20% with observations:

> *" For practitioners working with (symmetric) percentage differences between forecasts and observations, we further show that 9 (almost half) of the 21 time-varying $M_w \geq 3.95$ seismicity models provided earthquake counts that differed by 20% or less from the total number of observed earthquakes. Among these models are versions of STEP and ETAS, such as STEPJAVA, ETAS-SMOOTHING-V0, and STEP, which over periods of approximately 8, 6, and 11 years forecasted nearly 254, 398, and 678 $M_w \geq 3.95$ earthquakes, respectively, differing by 3%, 17% and 17% from the 246, 332 , and 579 events observed during such periods, respectively. "* (lines 92-99)

---

(8) A few larger earthquake sequences are separated out to test how well the models forecast the rate of earthquakes in the sequence. Please explain how the aftershock regions for each sequence were selected.

We used the scaling relationship of Wells and Coppersmith (1994) to determine the average fault length (L) of each analyzed main event and, based on this, we assumed that their aftershock zones are circular regions centered on their epicentral coordinates with radii equal to 3L. In the earlier version of the manuscript, we used radii equal to 5L, obtaining similar spatial test results, suggesting that these smaller aftershock zones contain all the aftershocks observed along the strike angle of the main ruptures. We have added this information in the caption of Fig. 6:

> *" Observed earthquakes are shown as turquoise squares and expected seismicity within the aftershock zones of the larger events, defined as circular regions with radii equal to three times their average fault lengths[70], are color-coded. "*

---

(9) The question of why the models performed poorly for the first 2 days of the El Mayor-Cucapah sequence should be actually investigated, instead of just hypothesizing. Please

check whether the catalogs provided for the models are indeed incomplete at the magnitude levels used by the models during the first few days. An incomplete catalog could affect both ETAS (not enough secondary triggering due to missing aftershocks) and STEP (underestimated productivity due to missing aftershocks.). The preferred explanation, that the productivity of the El Mayor earthquake was larger than the productivities of the models, needs to be supported, and should be checked by computing the productivity of that sequence using the same parameterization as the models. The Hardebeck et al. (2019) reference isn't helpful here because they don't compute a productivity for the El Mayor earthquake, nor do they compare their southern California Reasenberg-Jones productivity parameters with the productivity parameters of the CSEP models. I'll note also that much of the El Mayor-Cucapah aftershock zone is outside the Southern California Seismic Network, so is likely to have more incompleteness problems in general than inside California. Because of this, I think this is not an ideal event to provide much insight into general forecast performance.

We thank the reviewer for their valuable comments. Unfortunately, we have no access to the catalog that was provided to the models during the first days of the 2010 M_w 7.2 El Mayor-Cucapah earthquake sequence, which was likely delayed by at least 30 days to allow network operators/analysts time to reprocess some data. However, we have calculated short-term aftershock incompleteness (STAI) during the first two days of the sequence, following the methods of Helmstetter et al. (2006) for Southern California. We estimate that, for a mainshock of magnitude 7.2, the temporary magnitudes of completeness during the first and second days of the sequence are approximately 3.2 and 2.5, respectively, which are lower than the threshold magnitude of 3.95 used by the available models (Table S2), indicating that STAI issues do not fully explain the models' underestimations.

As mentioned in the text, we can also rule out that discrepancies between models and observations are primarily due to the use of generic aftershock productivity parameters, as

> *" [...] STEP, despite including a model component that estimates sequence-specific earthquake productivity as time progresses and data accumulate, also had difficulty forecasting triggered seismicity. "* (lines 284-289)

This leads to the conclusion that the sequence was indeed more productive than expected. To support this conclusion, we have plotted cumulative $M_w \geq 3.95$ earthquake counts observed and predicted during the first two weeks of the sequence (see the new Fig. S4). We note that the slopes of the curves for each model during the first two days deviate significantly from the one-to-one relationship, confirming our hypothesis.

We have also cited the work of Hardebeck et al. (2019) to allude only to

> *" [...] the diversity of productivity patterns observed more locally across the [CSEP-California testing] region[34,36]."* (lines 289-290)

Furthermore, we have narrowed the aftershock zone of the El Mayor-Cucapah to a circular area with a radius three times its average rupture length (see point 8 above and the updated Fig. 6), a large part of which lies within U.S. territory. We agree that seismic detectability is likely lower in Northern Mexico; however, this is beyond our control, as earthquakes know no borders. Despite these (geopolitical) limitations, we argue that this sequence is extremely useful for assessing the predictive skill of the models available, as it was very productive and its largest event is, to date, the largest earthquake to hit California since 1992 (Hauksson et al., 2011). For comparison, the 2010 M_w 7.2 El Mayor-Cucapah triggered 86 $M_w \geq 3.95$ events within the testing region during the first two weeks of the sequence, while the 2010 M_w 6.5 Ferndale, 2014 M_w 6.8 Mendocino, and 2014 M_w 6.0 South Napa earthquakes triggered only 8, 9, and 0 $M_w \geq 3.95$ earthquakes during the same period, respectively (Fig. 4).

(10) The models were not all active for the full 11 years, and were active over different times within the study period. I wonder if it biases the CSEP test results at all to have different numbers of test events for each model. For example, it requires some number of observations to falsify a model with a given level of confidence, so models that have been operational longer may be more falsifiable than newer models. Additionally, different models will have covered different sequences, and each sequence has different behaviors that may be more or less difficult to model. For example, all of the models operating at the time of the El Mayor-Cucapah earthquake struggled with this sequence, which contains a large fraction of the observed M ≥ 3.95 events. This suggests that simply being in operation at the time of this sequence may affect the overall test results for these models, again disproportionately affecting the older models.

We agree with the reviewer that models, particularly clustering models like these, are likely to fail when being assessed against more prospective data. In the revised manuscript, we explicitly mention that prospective test results based on longer time periods are more indicative of actual predictive skill and, in fact, consistency between forecasts and observations over longer periods are more impressive.

“ [...] (in)consistency test results based on more prospective data are more indicative of their actual predictive skill. ” (lines 107-108)

Regarding comparisons between models, the lack of data uniformity in model availability over time inevitably led us to conduct comparative analyses during "discretized" periods in which different model subsets were available (Fig. 8). We acknowledge that this lack of uniformity could bias the results (and interpretations) of the models, particularly those of the older ones:

“ Since the forecast database was implemented gradually over time (Fig. S1), we perform comparative analyses in six non-overlapping periods in which different model subsets were available, which could bias comparisons between models, particularly with older ones. ” (lines 210-212)

---

(11) The test of the spatial distributions includes only seismically active days. Please explain why it doesn’t consider all days. The spatial tests should penalize models that forecast a

high probability in a location with no earthquakes. The test as described appears to only include this penalty if an earthquake happened to occur somewhere else on that same day.

The CSEP binary spatial test is a conditional test designed to minimize the sensitivity of spatial log-likelihood scores to earthquake clustering (Bayona et al., 2022). This (in)consistency test, in particular, depends on simulating the number of observed activated cells per forecast window (one day in this case), which is why it is not "defined" during days without observed earthquakes. During seismically active days, the test does penalize models in cells where no seismicity is observed (see the second term in Eqs. 5 and 6).

(12) One conclusion is that the models that are nonparametric or with adaptive smoothing perform best in the spatial tests. This seems like an important result that could guide future model development. It would be helpful then to give some specific recommendations for how to achieve the right level of smoothing.

We agree that this is an important finding. In the revised manuscript, we have provided detailed information on the assumptions underlying these types of models, as well as detailed recommendations that could guide the development of operational earthquake forecasting models. For example:

> *" Broadly speaking, models that incorporated nonparametric components and "intermediate" smoothing procedures, such as adaptive smoothing, produced the most consistent spatial forecasts. E.g., the HW-KERNEL-M* models[16] which employ a power-law kernel with variable bandwidth, along with the ETAS-ADAPTIVE-M* models, which assume a power-law kernel for M < 5.5 triggering earthquakes and an anisotropic one for M ≥ 5.5 events, were among the most consistent with observations. In contrast, models such as ETAS-SMOOTHING-V0 and STEP, which predominantly use fixed isotropic smoothing functions, exhibited the poorest spatial performances, producing oversmoothed and overlocalized seismicity forecasts, respectively. The spatial limitations of ETAS-SMOOTHING-V0, nonetheless, appear to have been addressed by its successor model, ETAS-SMOOTHING-V1, which uses a comparatively smaller kernel bandwidth (see Fig. 1, resulting in improved spatial forecasting (see Figs. 8e and S9e). "* (lines 330-343)

> *" [...] based on these number and spatial test results and those presented here for California, we argue that future improvements to the OEF models in Italy could be achieved by: i) developing and prospectively testing new versions of STEP that use larger smoothing distances and/or assign greater weights to their anisotropic triggering kernel components; ii) developing and validating new flavors of ETAS that assume adaptive spatial kernels [...] "* (lines 417-421)

We have also included a new Table S1 summarizing the main features of each evaluated model.

(13) It's reported that models that were fit to both long catalogs of M≥3.95 earthquakes and shorter catalogs of smaller earthquakes perform well. It's hard to know what to make of this,

and it seems potentially contradictory. Here again more specific recommendations for future model development would be helpful.

> *" Regarding the number components, we show that, based on the CSEP Negative Binomial Distribution number test[30], 20 models statistically captured the cumulative number of observed earthquakes (see Fig. 3c). Some of the most consistent models, such as STEP(JAVA) and ETAS-SMOOTHING-V0, were calibrated on historical $M_w \geq 3.95$ seismicity catalogs dating back to 1932 and 1898, respectively, while other models, such as ETAS-HW-KERNEL-M*, ETAS-ADAPTIVE-M*, and K3-KERNEL-M*, used a more recent instrumental catalog containing $M_w \geq 2.0$ earthquakes (see Table S2). In the particular cases of ETAS-PPE-M2(.95) and ETAS-PPE-M3, the models also used a time-varying (PPE) background component[14] (see Table S1). Taken together, these results suggest that integrating historical earthquake records with higher-resolution seismicity catalogs and assuming time-varying background components are key elements to consider when developing seismicity models consistent with the total number of observed earthquakes in California. "* (lines 265-275).

---

(14) At some places, the manuscript makes vague and possibly overstated claims, with little support.

a. "Decadal" in the title is a bit of an overstatement, as most models did not run for 10 years.

While we understand the point raised by the reviewer, the title is accurate as select models are indeed consistent with decadal prospective observations in California. The now-called ETAS-SMOOTHING-V0 model, which was producing forecasts for 11 years, was statistically consistent with the number and magnitude distributions of observed $M_w \geq 3.95$ earthquakes (Figs. 3 and 7). Similarly, STEP and STEPJAVA, which are versions of the same model implemented in MatLab and Java, respectively, jointly demonstrated sustained consistency with the total number of earthquakes observed over more than a decade in California (Fig. 3). Throughout the revised version, however, we have made it explicit that not all models were available throughout the evaluation period.

b. The abstract could use fewer empty phrases like "marking a significant milestone", and more specifics about what was learned. Or, explain how this is a milestone, when there have been other similar CSEP comparisons of time-dependent models (e.g. Nanjo et al, 2012; Taroni et al., 2018; Rhoades et al, 2018).

Following this suggestion and the Nature Communications' guidelines for authors, we have removed the phrase and focused only on the main results of this study (see the revised abstract above).

c. The abstract implies that 27 models ran for a 11 year test period. This needs some caveats, because most of the models have been running for only about half that long.

Following the short abstract format required for Nature Communications, we make this information explicit throughout the revised manuscript. For example:

" This extensive set of increasingly complex earthquake forecasting models was gradually put into operation [...] " (line 32).

" We remind the reader that models were confronted against different numbers of earthquakes ω, because each has unique operationalization begin and end dates (Fig. S1). " (lines 105-107)

d. L19-21: Note that there has been substantial work in probabilistic earthquake forecasting that significantly pre-dates CSEP. This includes both long-term PSHA (e.g. Frankel et al, 2000), and time-dependent earthquake forecasts (e.g. Ogata, 1988; Reasenberg & Jones, 1989). Please acknowledge some of this prior work, and/or rewrite this sentence so that it doesn't overstate the role of CSEP in developing the field of probabilistic earthquake forecasting. CSEP has had a leading role in the push for prospective testing of forecasts, so it would be more appropriate to highlight that.

We have added references to the work of Ogata (1988) and Reasenberg and Jones (1989) to acknowledge their pioneering efforts in developing time-varying earthquake forecasting models. Furthermore, we have rephrased the sentence to more accurately reflect the role of CSEP in the field of earthquake forecasting:

" Thus, the lack of successful deterministic predictive models of earthquake occurrence has led seismologists, including members of the Collaboratory for the Study of Earthquake Predictability (CSEP)[4], to devote scientific efforts to forecasting earthquakes probabilistically[,5,6]. " (lines 19-21)

e. L27-28, L50: Please acknowledge here that most of the 27 models have not been operating for the full 11 years.

Following this suggestion, we have acknowledged that not all the models were prospectively generating earthquake forecasts during the entire 11-year evaluation period (see point c above and the updated figure 1).

f. L87-90 & 373-374: Why is this a milestone achievement, there are already other CSEP studies that were automatically run with no parameter value updating. Is there some sort of bar for public use that is newly achieved?

Previous CSEP next-day earthquake forecasting experiments involved far fewer models, less prospective data, and shorter evaluation periods, making these consistent number test results a milestone achievement for the statistical seismology community. As described in the main text, the operational earthquake forecasting (OEF) models for Italy, showed overall good agreement with observations, except for the 2012 and 2016-2017 earthquake sequences in central Italy, where empirical correction factors were applied in order to mitigate short-term catalog incompleteness issues (lines 400-412). Similarly, the OEF ETAS model for New Zealand significantly overestimated the number of observed earthquakes, partially due to magnitude recalibration problems (lines 423-430).

Here, we demonstrate that, with exception of the specific days on which the largest earthquakes occurred, over 80% of the available models produced statistically consistent forecasts with observations during the first two weeks of each sequence. In the case of the 2012 $M_w \geq 3.95$ Brawley seismic swarm, the models also captured the (lack of) observations, indicating that these types of models could be used, as a first approximation, to describe the spatiotemporal evolution of this type of seismicity. In all, these prospective test results provide confidence in the use of time-varying clustering models in public OEF.

g. L255: Is this remarkable? Similar types of models have performed well in other CSEP experiments, so it's not very surprising, especially since the rest of the paragraph acknowledges that many of the models are over or under-smooth.

We also consider this as remarkable because only a few (similar) next-day models have been shown to produce spatial forecasts consistent with the spatial distribution of observed earthquakes over extended periods. For example, in Italy, STEP has been found to produce overlocalized spatial forecasts compared with observations, whereas in this experiment, 15 different models based on nonparametric assumptions, adaptive smoothing, and anisotropic kernels have shown statistical consistency with the observed data. In the revised manuscript, we provide detailed information on the assumptions underlying some of the most spatially consistent models and compare these features with those of the models that provide the most inconsistent spatial forecasts (see point [12] above).

Figures:

Figure 1. State what the white areas mean. Is this where the models are undefined?

The white areas represent locations where the models are not defined. We have added this description to the caption of the updated Fig. 1:

> *" Earthquake rates are expressed per 0.1° x 0.1° cell per day, with bright colors denoting regions where seismic activity is comparatively high, dark colors indicating comparatively low rates, and white areas denoting regions where the models are not defined. "*

Figure 3b. Note that for models that started after 2007, their "cumulative number" starts at the observed cumulative number at the time the model started. Therefore, the total cumulative number for each model can't be directly compared.

The updated Fig. 3c includes the number of $M_w \geq 3.95$ earthquakes (ω) observed during the different periods in which the models were prospectively producing seismicity forecasts. Furthermore, we have explicitly described in the main text that each model was evaluated against a distinctive number of prospective earthquakes, because each has unique start and end dates (see point c above). The updated Fig. 3b also includes new vertical dashed lines indicating the dates on which different model subsets came into operation (see the specific numbers of new models installed above each line).

Figure 4. The way this is plotted, it looks like the time of the largest event perfectly aligns with the time of a forecast. However, actually each event occurred part way through a

forecast time period. I think that the forecast times are being rounded (or floored?) to integer days relative to the largest event. This plot would be more clear if the actual non-rounded relative times of the forecasts to the events were shown.

We have implemented the reviewer's suggestion. The updated figure 4 shows the time of day, relative to midnight, when the largest events occurred and how the models reacted to the increase in seismicity over the following 15 days.

Figure 5. Like the main text, this figure capture could use some explanation of what observation is at the given quantile of what distribution.

“ [...] we perform CSEP binary spatial tests to assess, for each model and for each of the days between August 2007 and August 2018 on which $M_w \geq 3.95$ earthquakes were recorded in California, the log-likelihoods (referred to here as jbill values) of observing either no seismicity or at least one target earthquake in each of the forecasts' spatial grid cells (see the Methods section). This test is summarized by the observed spatial quantile ζ, which is the fraction of simulated catalogs of target activated cells with spatial log-likelihoods smaller than the jbill observed on a seismically active day. If ζ > 0.05, then the spatial distributions of observed and predicted earthquakes can be considered statistically indistinguishable.

By sorting for each model all the observed spatial quantiles in ascending order (pink curves in Fig. 5) and comparing these distributions with spatial quantiles drawn from uniform distributions (gray dashed diagonals), we show that 15 (~71%) of the 21 time-varying $M_w \geq 3.95$ seismicity models generated daily earthquake forecasts statistically consistent with the spatial distribution of observed epicenters (see the pink areas [A] between the curves and the diagonals; the smaller the area, the greater the agreement between forecasts and observations). ” (lines 150-163)

“ The shape and position of the pink curves relative to the diagonals in Fig.5 provide additional information on the level of spatial precision that the models can offer. In particular, if the pink curves lie below the diagonals, this indicates that models generated spatially overly smoothed forecasts compared to observations, whereas, if the pink curves lie above the diagonals, this denotes that models provided spatial forecasts that are too localized or inaccurate [...] ” (lines 169-173)

Figure 6. I don't see the gray polygons showing the aftershock zones. Is it just the region with colored grid points? Some panels also have cyan squares, are these the aftershock zones? Are the ζ values for ETAS-DROneDayMd2 and ETAS-DROneDayMd3 really 0?

We have deleted the gray polygons to show the aftershock zones of the analyzed main earthquakes as colored grid points. The cyan squares indicate the locations of observed earthquakes, with sizes proportional to magnitudes. The (now called) ETAS-PPE-M* models obtained ζ scores of 0.0, because the models expected relatively low rates in those dark orange

cells where some of the triggered earthquakes occurred (note that the color scale in Fig. 6 is logarithmic), thereby obtaining comparatively low log-likelihood spatial scores (or jbill values). These jbill values were estimated to be smaller than the distribution of spatial scores obtained from simulating activated cells from the forecasts, leading to ζ=0.0 and indicating inconsistency with observations. In other words, the forecasts were too localized compared to observations. In fact, in the particular case of ETAS-PPE-M3, we quantify that the model provides spatially overconfident forecasts with statistical significance (Fig. 5), which can also be seen in Fig. 6 (note the close proximity between very low-probability dark cells and higher probability warmed-colored cells).

Minor points & typos:

L33: STEP stands for Short-Term Earthquake Probability

Corrected (lines 33-34).

L72: "variability of earthquakes ascribed" -> "variability of earthquake rate due to"

Rephrased to "*variability of earthquake rates due to*" (lines 80-81).

L82-87: Please remind the reader that each model may have a different number of events because each has unique begin and end dates.

" We remind the reader that models were confronted against different numbers of earthquakes ω, because each has unique operationalization start and end dates (Fig. S1)." (lines 105-107).

L130: Briefly explain the spatial quantile tests, otherwise this is hard to follow.

We refer the reviewer to their comment on Fig. 5.

L132, L211: Earlier (L27) it says there are 27 models, what happened to the other 6?

The CSEP community developed and automated 27 next-day seismicity models for California; 21 models forecast $M_w \geq 3.95$ seismicity while 6 models forecast $M_w \geq 4.95$ earthquakes.

> *" We focus on a model group of 21 $M_w \geq 3.95$ seismicity models because abundant data are available for testing and because OEF systems forecast earthquakes at approximately this magnitude threshold. Nonetheless, prospective test results for a subset of 6 $M_w \geq 4.95$ earthquake forecasting models can also be found in the supplementary material for the article. "* (lines 64-67).

L145: Define ζ or don't use it. Explain what these numbers quantify: quantile of what compared to what distribution.

The observed spatial quantile ζ has been defined in the revised manuscript (see comments above).

L214: Strike "decadal". None of these models ran for a full decade.

The word has been removed: *" [...] the most consistent with prospective observations."* (line 265).

L272: Include a reference for the USGS finite fault model, e.g. Goldberg et al. (2022)

Reference has been included (line 353).

L307: "rate-and-state fraction" -> "rate-and-state friction"

Corrected (lines 399-400).

L317: magnitude is missing

Magnitudes have been included: *" [...] with the exceptions of the 2012 M_w 6.1 Emilia and 2016-2017 Amatrice-Norcia M_w 6.6 earthquake sequences. "* (line 411).

L346: "U.S. territories and commonwealths" -> "U.S. states and territories"

Rephased to *" U.S. states and territories "* (lines 442-443).

L347: The USGS produces forecasts only for international earthquakes with estimated fatalities >100, not for all Mw ≥ 5.0 international earthquakes.

We thank the reviewer for the clarification and have added this information: *"[...] a manual system for particularly complex domestic sequences and international earthquakes with estimated fatalities exceeding 100."* (lines 343-444).

L384: This actually seems like a problem if the public is questioning the reliability of the forecasts. CSEP can assure people that the models being used have performed adequately in testing.

The reliability of a model can be defined in terms of its calibration, meaning that its predictive probabilities align well with the observed likelihood of real-world outcomes. For example, if a perfectly calibrated model predicts an event with 95% confidence, that event should occur exactly 95% of the time in the long run. To quantify and visualize model reliability, one could generate calibration plots, such as those shown in Fig. 5 (see Brehmer et al., 2025, for other examples). Clearly, further prospective testing will be required to identify the most reliable forecasting models; however, our test results could be useful in discerning which are the best candidates and how models could be improved.

L437: redundant: "high proportion of low quantiles and a high proportion of low quantiles"

We have removed the redundant information:

> *[...] if the observed quantile distributions (pink curves in Fig. 5) lie above the one-to-one relationship (i.e., the uniform distribution shown in gray), this indicates a high proportion of low quantiles compared with a uniform distribution, suggesting overly localized or inaccurate spatial forecasts. Conversely, if the general trend of each curve lies below the one-to-one line, this indicates a low proportion of low quantiles compared with a uniform distribution, suggesting overly dispersed spatial forecasts [...] "* (lines 548-553)

L457: I think the pink curve is the observed.

The reviewer is right. We have corrected the sentence:

> *"If the observed incremental frequency-magnitude distributions (pink curves) fall within these intervals, models and observations can be considered statistically indistinguishable."* (lines 570-572)

L460: The notation K_S keeps getting reused for different tests. I would be clearer if the p-value for each K-S test had a different notation, e.g. p_mag.

In the revised manuscript, we now use K_s and K_m when referring to the *p*-values of the Kolmogorov-Smirnov tests applied to the space and magnitude components of the models, respectively (see Figs. 5 and 7 and Eq. 9). For example:

> *" [...] we complement this analysis with nonparametric Kolmogorov-Smirnov tests (see the Methods section). We find that, with the exception of STEPJAVA, all time-varying models provided magnitude forecasts statistically consistent with observations, obtaining K_m values (p-values) greater than 0.05. "* (lines 197-199)
>
> *" [...] we perform nonparametric Kolmogorov-Smirnov tests to determine whether we can reject the null hypothesis that each observed spatial quantile distribution is drawn from a uniform quantile distribution. In this case, if the p-value of the test, K_s, is smaller than 0.05, we can conclude that a significant difference between both distributions exists. "* (lines 556-559)

L474: "M_x=1" -> "M_1", and "M_x=2" -> "M_2".

Corrected: *" [...] two competing models Mx (with x = 1, 2) [...] "* (line 587).

L478: missing close parenthesis

Parenthesis is closed now (line 592).

Figure 4: "20% or fewer of them do not statistically match" -> "20% or fewer of them statistically match"

In the caption of the updated figure 4, we have generalized this description to: *" The colors of the circles represent the percentage (Z) of available models that capture the observed daily counts (see the colorbar). "*

REFERENCES

Bayona, J.A., Savran, W.H., Rhoades, D.A. and Werner, M.J., 2022. Prospective evaluation of multiplicative hybrid earthquake forecasting models in California. *Geophysical Journal International*, *229*(3), pp.1736-1753.

Bird, P., 2018. Ranking some global forecasts with the Kagan information score. *Seismological Research Letters*, *89*(4), pp.1272-1276.

Brehmer, J.R., Kraus, K., Gneiting, T., Herrmann, M. and Marzocchi, W., 2025. Enhancing the statistical evaluation of earthquake forecasts—An application to Italy. *Seismological Research Letters*, *96*(3), pp.1966-1988.

Felzer, K.R., 2007. Appendix I: calculating California seismicity rates. *US Geol. Surv. Open-File Rept. 2007-1437I*.

Field, E.H., 2007. Overview of the working group for the development of regional earthquake likelihood models (RELM). *Seismological Research Letters*, *78*(1), pp.7-16.

Hardebeck, J.L., Llenos, A.L., Michael, A.J., Page, M.T. and Van Der Elst, N., 2019. Updated California aftershock parameters. *Seismological Research Letters*, *90*(1), pp.262-270.

Hauksson, E., Stock, J., Hutton, K., Yang, W., Vidal-Villegas, J.A. and Kanamori, H., 2011. The 2010 M w 7.2 el mayor-cucapah earthquake sequence, Baja California, Mexico and southernmost California, USA: active seismotectonics along the Mexican pacific margin. *Pure and Applied Geophysics*, *168*(8), pp.1255-1277.

Helmstetter, A., Kagan, Y.Y. and Jackson, D.D., 2006. Comparison of short-term and time-independent earthquake forecast models for southern California. *Bulletin of the Seismological Society of America*, *96*(1), pp.90-106.

Iturrieta, P., Savran, W.H., Herrmann, M., Bayona, J.A., Gerstenberger, M.C., Graham, K., Maechling, P.J., Marzocchi, W., Mizrahi, L., Schorlemmer, D. and Serafini, F., 2026. floatCSEP: An application to deploy and conduct reproducible prospective earthquake forecasting experiments. *Journal of Open Source Software*, *11*(118), p.9408.

Kagan, Y.Y. and Jackson, D.D., 2010. Short-and long-term earthquake forecasts for California and Nevada. *Pure and Applied Geophysics*, *167*(6), pp.685-692.

Llenos, A.L. and Michael, A.J., 2017. Forecasting the (un) productivity of the 2014 M 6.0 South Napa aftershock sequence. *Seismological Research Letters*, *88*(5), pp.1241-1251.

Neo, J.C., Huang, Y., Yao, D. and Wei, S., 2021. Is the aftershock zone area a good proxy for the mainshock rupture area?. *Bulletin of the Seismological Society of America*, *111*(1), pp.424-438.

Parsons, T., Segou, M., Sevilgen, V., Milner, K., Field, E., Toda, S. and Stein, R.S., 2014. Stress-based aftershock forecasts made within 24 h postmain shock: Expected north San Francisco Bay area seismicity changes after the 2014 M= 6.0 West Napa earthquake. *Geophysical Research Letters*, *41*(24), pp.8792-8799.

Savran, W.H., Bayona, J.A., Iturrieta, P., Asim, K.M., Bao, H., Bayliss, K., Herrmann, M., Schorlemmer, D., Maechling, P.J. and Werner, M.J., 2022. pyCSEP: A Python toolkit for earthquake forecast developers. *Seismological Society of America*, *93*(5), pp.2858-2870.

Schorlemmer, D. and Gerstenberger, M.C., 2007. RELM testing center. *Seismological Research Letters*, *78*(1), pp.30-36.

Serafini, F., Bayona, J.A., Silva, F., Savran, W., Stockman, S., Maechling, P.J. and Werner, M.J., 2025. A benchmark database of ten years of prospective next-day earthquake forecasts in California from the Collaboratory for the Study of Earthquake Predictability. *Scientific Data*, *12*(1), p.1501.

Strader, A., Werner, M., Bayona, J., Maechling, P., Silva, F., Liukis, M. and Schorlemmer, D., 2018. Prospective evaluation of global earthquake forecast models: 2 yrs of observations provide preliminary support for merging smoothed seismicity with geodetic strain rates. *Seismological Research Letters*, *89*(4), pp.1262-1271.

Wells, D.L. and Coppersmith, K.J., 1994. New empirical relationships among magnitude, rupture length, rupture width, rupture area, and surface displacement. *Bulletin of the seismological Society of America*, *84*(4), pp.974-1002.
